# Lewis acid-catalyzed redox-neutral amination of 2-(3-pyrroline-1-yl)benzaldehydes via intramolecular [1,5]-hydride shift/isomerization reaction

**DOI:** 10.3762/bjoc.10.306

**Published:** 2014-12-05

**Authors:** Chun-Huan Jiang, Xiantao Lei, Le Zhen, Hong-Jin Du, Xiaoan Wen, Qing-Long Xu, Hongbin Sun

**Affiliations:** 1 State Key Laboratory of Natural Medicines and Jiangsu Key Laboratory of Drug Discovery for Metabolic Disease, China Pharmaceutical University, 24 Tongjia Xiang, Nanjing 210009, China

**Keywords:** catalysis, C–H activation, hydride-shift, Lewis acid, redox reaction

## Abstract

Lewis acid-catalyzed redox-neutral amination of 2-(3-pyrroline-1-yl)benzaldehydes via intramolcular [1,5]-hydride shift/isomerization reaction has been realized, using the inherent reducing power of 3-pyrrolines. A series of *N*-arylpyrrole containing amines are obtained in high yields.

## Introduction

The direct and selective functionalization of the inactive C(sp^3^)–H bond constitute an economically attractive strategy for organic syntheses [1–10]. Until now, a number of transition metals can be used for the activation of C–H bonds [11–18]. Among the reported transformations, intramolecular redox processes based on direct functionalization of C(sp^3^)–H bonds linking with α heteroatoms are useful for the synthesis of structurally diverse amines and ether derivatives [19–30]. On the other hand, compounds containing the *N*-arylpyrrole moiety serve as important building blocks for the synthesis of various complex molecules and exhibit a larger number of biological effects [31–33].

**Scheme 1:**
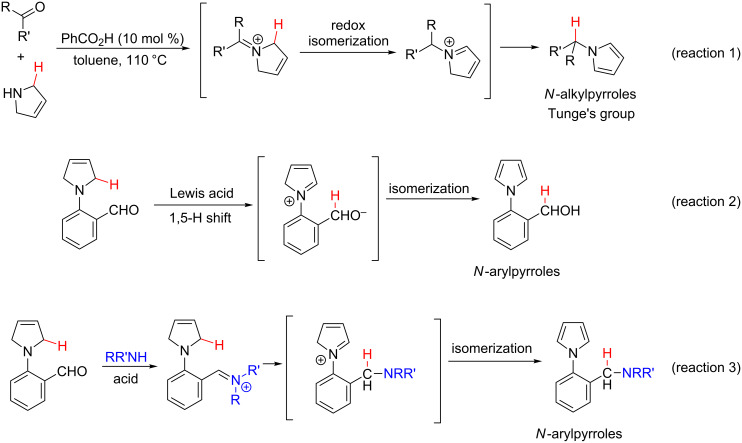
Synthesis of N-substituted pyrroles via redox-neutral reaction.

In 2009, Tunge's group disclosed that *N*-alkylpyrroles could be formed via a redox isomerization reaction (Scheme 1, reaction 1) [34–36]. Moreover, we recently realized a Lewis acid-catalyzed intramolecular redox reaction using an aldehyde group as the H-shift acceptor to afford (2-(1*H*-pyrrol-1-yl)phenyl)methanol (Scheme 1, reaction 2) [37]. As part of our interest in expanding the inherent reducing power of 3-pyrrolines, we report herein the Lewis acid-catalyzed redox-neutral amination of 2-(3-pyrroline-1-yl)benzaldehydes using the iminium group as the H-shift acceptor (Scheme 1, reaction 3). Notably, this reaction should meet the requirement that the iminium formation reaction should be faster than the aldehyde redox reaction.

## Results and Discussion

In our initial investigation, aldehyde **1a** and dibenzylamine (**2a**) were chosen as the model reaction substrates. In the presence of 10 mol % PhCOOH, the reaction of **1a** with **2a** in DCE at rt for 24 h gave the trisubstituted amine **3a** in 50% yield (entry 1, Table 1). Encouraged by this result, we screened readily available Brønsted and Lewis acids (Table 1). Except the Lewis acid AlCl_3_, other strong Brønsted acids and common Lewis acids could be used as the catalyst in this reaction, affording the desired products in excellent yields (entries 2–8, Table 1). Considering that ZnCl_2_ is cheaper and easy to handle, it was chosen as the catalyst for further optimization reactions. Furthermore, various solvents such as DCE, CH_2_Cl_2_, CHCl_3_, toluene, CH_3_CN and THF were examined. All the solvents afforded the desired product in satisfactory yields (entries 8–13, Table 1). Subsequently, the loading of dibenzylamine (**2a**) and the catalyst was examined. The results show that decreasing the amount of **2a** to 1.2 equiv and ZnCl_2_ to 5 mol % did not affect the yield (entry 16, Table 1).

**Table 1:** Optimization of the redox-neutral amination reaction.^a^

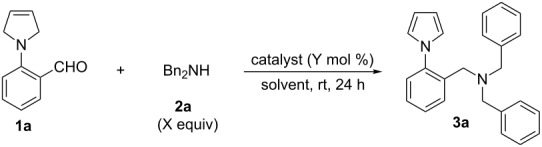					

entry	catalyst	X	Y	solvent	yield (%)^b^

1	PhCOOH	1.5	10	DCE	50
2	CF_3_COOH	1.5	10	DCE	87
3	*p*-TsOH**^.^**H_2_O	1.5	10	DCE	90
4	Sc(OTf)_3_	1.5	10	DCE	94
5	Cu(OTf)_2_	1.5	10	DCE	94
6	Zn(OTf)_2_	1.5	10	DCE	97
7	AlCl_3_	1.5	10	DCE	76
8	ZnCl_2_	1.5	10	DCE	95
9	ZnCl_2_	1.5	10	CH_2_Cl_2_	97
10	ZnCl_2_	1.5	10	CHCl_3_	95
11	ZnCl_2_	1.5	10	toluene	94
12	ZnCl_2_	1.5	10	CH_3_CN	96
13	ZnCl_2_	1.5	10	THF	71
14	ZnCl_2_	1.2	10	CH_2_Cl_2_	97
15	ZnCl_2_	1.0	10	CH_2_Cl_2_	93
16	ZnCl_2_	1.2	5	CH_2_Cl_2_	95
17	ZnCl_2_	1.2	2	CH_2_Cl_2_	91

^a^**1a** (0.5 mmol), **2a** (X equiv), catalyst (Y mol %), solvent (5 mL), room temperature, 24 h. ^b^Isolated yield.

Finally, we established the optimized reaction conditions using ZnCl_2_ (5 mol %) as the catalyst and CH_2_Cl_2_ as the solvent, and running the reaction at room temperature or under reflux.

**Scheme 2:**
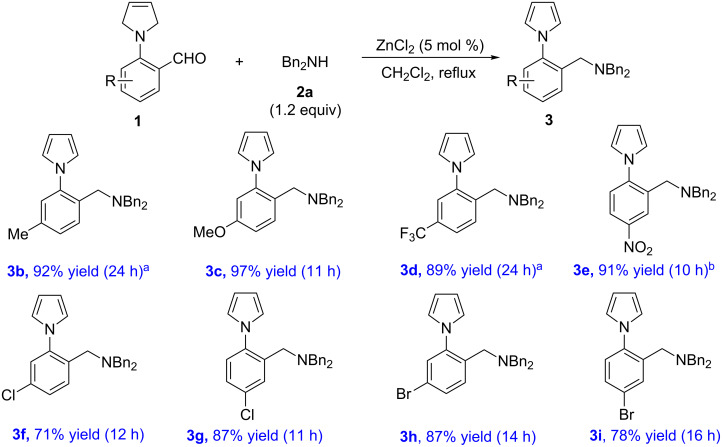
Substrate scope of aryl aldehydes **1**. Reagents and conditions: **1** (0.3 mmol), **2a** (1.2 equiv), ZnCl_2_ (5 mol %), CH_2_Cl_2_ (3.0 mL). ^a^Room temperature. ^b^DCE, reflux.

Under the optimized conditions, the results of the amination reaction of **2a** with various 2-(3-pyrroline-1-yl)benzaldehydes **1** are shown in Scheme 2. The reactions proceeded smoothly to give the corresponding *N*-arylpyrrole amines **3** in good to excellent yields (71–97% yields). Notably, the substitution of the benzene ring had little effect on the reaction since both electron-donating (**3b**, **3c**) and electron-withdrawing groups (**3d–i**) were tolerated in the reaction.

**Scheme 3:**
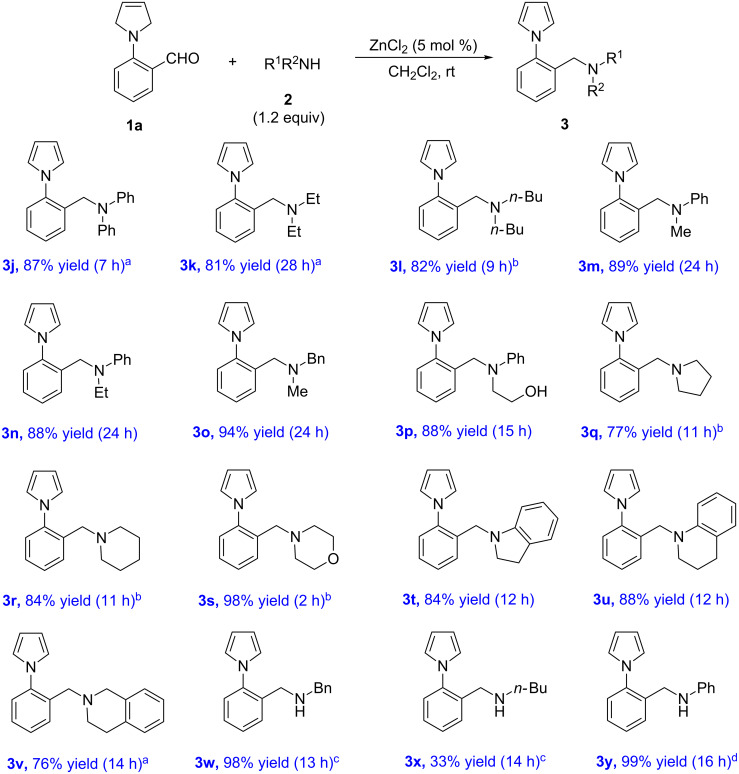
Substrate scope of amines **2**. Reagents and conditions: **1a** (0.5 mmol), **2** (1.2 equiv), ZnCl_2_ (5 mol %), CH_2_Cl_2_ (5.0 mL), rt. ^a^Reflux. ^b^DCE, reflux. ^c^Zn(OTf)_2_ (5 mol %), *n*-butylamine (5.0 equiv), DCE, reflux. ^d^*p*-TsOH**·**H_2_O (5 mol %), PhNH_2_ (5.0 equiv), CH_2_Cl_2_, rt.

Next, the scope of amines **2** was explored. The results are summarized in Scheme 3. Reaction of secondary amines possessing aryl–aryl, alkyl–alkyl and aryl–alkyl moieties yield the corresponding *N*-arylpyrrole amines **3j**–**p** in high yields (81–94% yields). Various cyclic secondary amines were also good substrates for this reaction, affording the desired products (**3q**, **3r**, **3s**) in good to high yields (77–98% yields) with DCE as the solvent under reflux conditions. The reaction with indoline, tetrahydroquinoline, and tetrahydroisoquinoline could also be realized to give products **3t**, **3u**, and **3v** in good yields (76–88% yields), respectively. Finally, primary amines were examined. The reaction with excess benzylamine (5.0 equiv) in the presence of Zn(OTf)_2_ as the catalyst afforded the desired product **3w** in 98% yield. However, when *n*-BuNH_2_ was used as the substrate, the yield was reduced to 33% even under high temperature. Notably, according to the ^1^H NMR spectrum of the crude product, the reaction with phenylamine using Zn(OTf)_2_ as the catalyst afforded only the corresponding imine product, indicating that the [1,5]-hydride shift/isomerization reaction did not occur. To our delight, this reaction proceeded smoothly at room temperature to give the desired *N*-arylpyrrole amine **3y** in high yield (99% yield) when *p*-TsOH·H_2_O was used as the catalyst instead of Zn(OTf)_2_.

## Conclusion

In conclusion, Lewis acid-catalyzed redox-neutral amination of 2-(3-pyrroline-1-yl)benzaldehydes via intramolcular [1,5]-hydride shift/isomerization reaction has been realized. Various types of amines and 2-(3-pyrroline-1-yl)benzaldehydes are well tolerated in this reaction, affording the corresponding *N*-arylpyrrolamines in good to high yields. Further studies on synthetic applications of [1,5]-hydride shift/isomerization reactions that utilize the inherent reducing power of 3-pyrrolines are underway in our laboratory.

## Experimental

**General procedure for the preparation of *N*-arylpyrroles 3**: A mixture of benzaldehyde **1** (0.3–0.5 mmol), amine **2** (1.2 equiv) and ZnCl_2_ (5 mol %) were stirred in dichloromethane or DCE (5.0 mL) at room temperature or reflux and monitored by TLC. After completion of the reaction (about 24 h), the solvent was removed by evaporation and the residue was purified by flash column chromatography on silica gel to give *N*-arylpyrrole **3**.

## Supporting Information

File 1 Experimental details, analytical data, and copies of the ^1^H and ^13^C NMR spectra of the final products.
