# Supplementary material for: Lewis acid-catalyzed redox-neutral amination of 2-(3-pyrroline-1-yl)benzaldehydes via intramolecular [1,5]-hydride shift/isomerization reaction
Source: Beilstein J Org Chem. 2014 Dec 5;10:2892–6. doi: 10.3762/bjoc.10.306 (PMC4273273; doi:10.3762/bjoc.10.306)
Supplement: File 1 — Experimental details, analytical data, and copies of the 1H and 13C NMR spectra of the final products. [file Beilstein_J_Org_Chem-10-2892-s001.pdf]

Supporting Information

for

Lewis acid-catalyzed redox-neutral amination of 2-(3-pyrroline-1-yl)benzaldehydes via intramolecular [1,5]-hydride shift/isomerization reaction

Chun-Huan Jiang, Xiantao Lei, Le Zhen, Hong-Jin Du, Xiaoan Wen, Qing-Long Xu* and Hongbin Sun*

Address: State Key Laboratory of Natural Medicines and Jiangsu Key Laboratory of Drug Discovery for Metabolic Disease, China Pharmaceutical University, 24 Tongjia Xiang, Nanjing 210009, China

Email: Qing-Long Xu - xuqing307@126.com, Hongbin Sun - hbsun2000@yahoo.com

*Corresponding author

Experimental details, analytical data, and copies of the ^{1}H and ^{13}C NMR spectra of the final products

Table of Contents

General information……………………………………….....................................S2

General procedure for the preparation of compound **1**..S2

General procedure for the preparation of *N*-arylpyrroles..S3

References...S11

Copies of NMR spectra...S12

General information

^{1}H NMR (300 MHz) spectra were recorded on a Bruker Avance 300 spectrometer in $CDCl_3$ [using $(CH_3)_4Si$ (for ^{1}H, δ = 0.00) as internal standard]. ^{13}C NMR (75 MHz) spectra on a Bruker Avance 300 spectrometer in $CDCl_3$ [using $CDCl_3$ (for ^{13}C, δ = 77.00) as internal standard]. The following abbreviations were used to describe peak splitting patterns when appropriate: s = singlet, d = doublet, t = triplet, q = quartet, m = multiplet, dd = doublet of doublets, dt = doublet of triplets, ddd = doublet of doublet of doublets, td = triplet of doublets. High and low resolution mass spectra were obtained with a ESI mass spectrometer and a TOF mass analyzer. Melting points are uncorrected.

Commercially available reagents and solvents were used without further purification. Flash column chromatography was performed using silica gel (200–300 mesh, Qingdao Ocean Chemical Company, China). Reactions were monitored by TLC on silica gel 60 F254 plates (Qingdao Ocean Chemical Company, China); visualization was effected by ultraviolet light (254 nm).

General procedure for the preparation of compound 1[1]

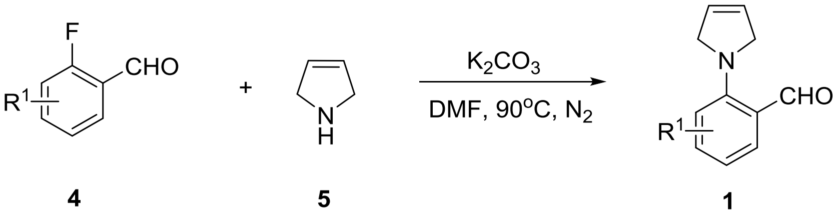

To a solution of 2-fluorobenzaldehyde **4a** (1.8 g, 14.5 mmol, 1.0 equiv) in DMF was added K_2CO_3 (2.0 g, 14.5 mmol, 1.0 equiv) and 3-pyrroline **5** (1.0 g, 14.5 mmol, 1.0 equiv) at rt, then the mixture was heated to 90 ^{o}C and stirred for 7 h. The mixture was filtered and the filtrate was concentrated and the residue was purified by column chromatography (PE/EA 25:1) to give the title compound 1 (1.55 g, 62% yield).

General procedure for the preparation of *N*-arylpyrroles

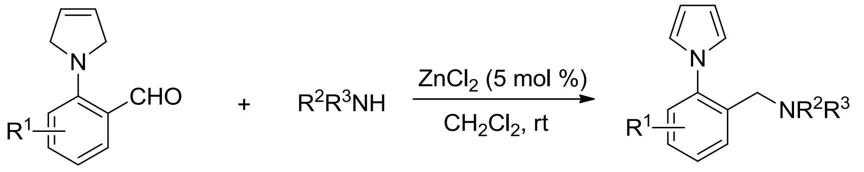

1 **2** (1.2 equiv.) **3**

A mixture of benzaldehyde **1** (0.3–0.5 mmol), amine **2** (1.2 equiv) and $ZnCl_2$ (5 mol %) were stirred in dichloromethane or DCE (5.0 mL) at room temperature or reflux and monitored by TLC. After the completion of the reaction (about 24 h), the organic solvent was removed by evaporation and the residue was purified by flash column chromatography on silica gel to give *N*-arylpyrrole **3**.

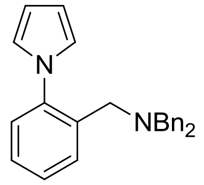

3a

3a, 93% yield, light yellow solid (R_f = 0.85, petroleum ether/ethyl acetate, 50/3), m.p. 69.1–71.8 °C.
^{1}H-NMR (300 MHz, $CDCl_3$) δ 7.87 (d, *J* = 7.6 Hz, 1H), 7.48-7.04 (m, 13H), 6.73 (t, *J* = 2.0 Hz, 2H), 6.30 (t, *J* = 2.0 Hz, 2H), 3.43 (s, 4H), 3.40 (s, 2H).
^{13}C-NMR (75 MHz, $CDCl_3$) δ 140.6, 139.4, 135.8, 130.0, 128.7, 128.2, 127.8, 127.2, 126.9, 126.7, 122.5, 108.8, 58.1, 52.5.
MS (ESI): *m/z* 353.2 $[M+H]^+$; **HRMS** (ESI): Exact mass calcd. for $C_{25}H_{25}N_2$ $[M+H]^+$ 353.2018, Found: 353.2028.

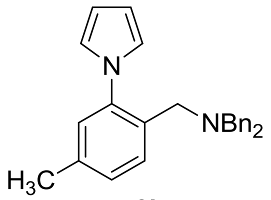

3b

3b, 92% yield, light yellow oil (R_f = 0.85, petroleum ether/ethyl acetate, 50/3).
^{1}H NMR (300 MHz, $CDCl_3$) δ 7.72 (d, *J* = 7.9 Hz, 1H), 7.38-7.23 (m, 8H), 7.19 (t, *J* = 6.9 Hz, 3H), 7.02 (s, 1H), 6.73 (t, *J* = 1.9 Hz, 2H), 6.29 (t, *J* = 1.9 Hz, 2H), 3.42 (s, 4H), 3.37 (s, 2H), 2.32 (s, 3H).
^{13}C NMR (75 MHz, $CDCl_3$) δ 140.4, 139.5, 137.2, 132.4, 129.8, 128.7, 128.5, 128.2, 127.2, 126.8, 122.5, 108.7, 58.0, 52.4, 20.8.
MS (ESI): *m/z* 389.2 $[M+Na]^+$; **HRMS** (ESI): Exact mass calcd. for $C_{26}H_{26}N_2Na$ $[M+Na]^+$ 389.1994, Found: 389.1985.

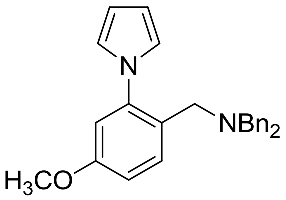

3c

3c, 97% yield, light yellow oil (R_f = 0.85, petroleum ether/ethyl acetate, 50/3).

^{1}H NMR (300 MHz, $CDCl_3$) δ 7.71 (d, *J* = 8.6 Hz, 1H), 7.45-7.10 (m, 10H), 6.94 (dd, *J* = 8.6, 2.3 Hz, 1H), 6.76 (s, 3H), 6.30 (s, 2H), 3.76 (s, 3H), 3.42 (s, 4H), 3.34 (s, 2H).

^{13}C NMR (75 MHz, $CDCl_3$) δ 158.6, 141.4, 139.5, 131.1, 128.7, 128.2, 127.4, 126.8, 122.5, 114.1, 111.6, 108.8, 57.9, 55.4, 52.2.

MS (ESI): *m/z* 405.2 $[M+Na]^+$; **HRMS** (ESI): Exact mass calcd. for $C_{26}H_{26}N_2ONa$ $[M+Na]^+$ 405.1943, Found: 405.1938.

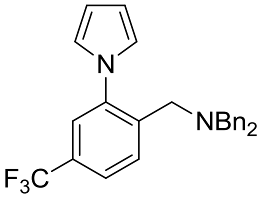

3d

3d, 89% yield, light yellow oil (R_f = 0.85, petroleum ether/ethyl acetate, 50/3),.

^{1}H NMR (300 MHz, $CDCl_3$) δ 8.02 (d, *J* = 8.1 Hz, 1H), 7.62 (d, *J* = 8.2 Hz, 1H), 7.47 (s, 1H), 7.37-7.10 (m, 10H), 6.72 (t, *J* = 2.0 Hz, 2H), 6.33 (t, *J* = 2.0 Hz, 2H), 3.45 (s, 6H).

^{13}C NMR (75 MHz, $CDCl_3$) δ 140.7, 140.1, 138.9, 130.5, 129.8 (q, *J* = 32.9 Hz), 128.7, 128.4, 127.1, 124.4 (q, *J* = 3.6 Hz), 123.7 (q, *J* = 270.4 Hz), 123.6 (q, *J* = 3.7 Hz), 122.4, 109.6, 58.3, 52.4.

MS (ESI): *m/z* 443.2 $[M+Na]^+$; **HRMS** (ESI): Exact mass calcd. for $C_{26}H_{23}F_3N_2Na$ $[M+Na]^+$ 443.1711, Found: 443.1721.

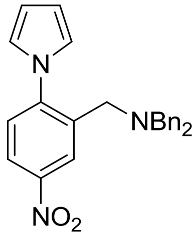

3e

3e, 91% yield, light yellow oil (R_f = 0.85, petroleum ether/ethyl acetate, 50/3).

^{1}H NMR (300 MHz, $CDCl_3$) δ 8.78 (d, *J* = 2.1 Hz, 1H), 8.06 (dd, *J* = 8.6, 2.3 Hz, 1H), 7.41-7.11 (m, 11H), 6.76 (s, 2H), 6.36 (s, 2H), 3.55 (s, 2H), 3.49 (s, 4H).

^{13}C NMR (75 MHz, $CDCl_3$) δ 146.6, 145.3, 138.5, 136.8, 128.8, 128.4, 127.2, 126.8, 125.8, 122.5, 122.2, 110.3, 58.5, 52.6.

MS (ESI): *m/z* 420.2 $[M+Na]^+$; **HRMS** (ESI): Exact mass calcd. for $C_{25}H_{23}N_3O_2Na$ $[M+Na]^+$ 420.1688, Found: 420.1676.

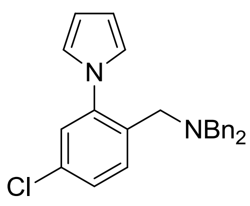

3f

3f, 71% yield, light yellow oil (R_f = 0.85, petroleum ether/ethyl acetate, 50/3).
^{1}H NMR (300 MHz, $CDCl_3$) δ 7.84 (d, *J* = 1.8 Hz, 1H), 7.36-7.24 (m, 8H), 7.23-7.14 (m, 3H), 7.09 (d, *J* = 8.4 Hz, 1H), 6.66 (s, 2H), 6.29 (s, 2H), 3.42 (s, 4H), 3.35 (s, 2H).
^{13}C NMR (75 MHz, $CDCl_3$) δ 139.0, 138.9, 137.8, 133.6, 129.7, 128.7, 128.3, 127.9, 127.4, 127.1, 122.4, 109.2, 58.2, 52.3.
MS (ESI): *m/z* 387.2 $[M+H]^+$; **HRMS** (ESI): Exact mass calcd. for $C_{25}H_{24}ClN_2$ $[M+H]^+$ 387.1628, Found: 387.1641.

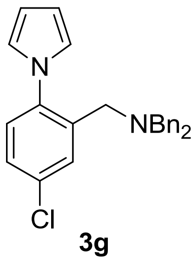

3g

3g, 87% yield, light yellow oil (R_f = 0.85, petroleum ether/ethyl acetate, 50/3).
^{1}H NMR (300 MHz, $CDCl_3$) δ 7.79 (d, *J* = 8.4 Hz, 1H), 7.43-7.09 (m, 12H), 6.81-6.61 (m, 2H), 6.36-6.17 (m, 2H), 3.42 (s, 4H), 3.36 (s, 2H).
^{13}C NMR (75 MHz, $CDCl_3$) δ 141.4, 139.1, 134.2, 132.5, 131.2, 128.7, 128.3, 127.9, 127.0, 126.6, 122.3, 109.4, 58.1, 52.1.
MS (ESI): *m/z* 387.2 $[M+H]^+$; **HRMS** (ESI): Exact mass calcd. for $C_{25}H_{24}ClN_2$ $[M+H]^+$ 387.1628, Found: 387.1635.

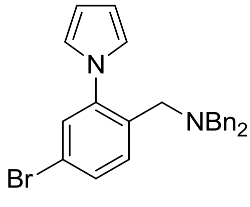

3h

3h, 87% yield, light yellow oil (R*f* = 0.85, petroleum ether/ethyl acetate, 50/3).
^{1}H NMR (300 MHz, $CDCl_3$) δ 7.72 (d, *J* = 8.4 Hz, 1H), 7.46 (dd, *J* = 8.3, 1.9 Hz, 1H), 7.38-7.09 (m, 11H), 6.82-6.55 (m, 2H), 6.42-6.08 (m, 2H), 3.41 (s, 4H), 3.34 (s, 2H).
^{13}C NMR (75 MHz, $CDCl_3$) δ 141.6, 139.0, 134.8, 131.4, 130.8, 129.5, 128.6, 128.3, 127.0, 122.3, 120.2, 109.4, 58.1, 52.1.
MS (ESI): *m/z* 431.1 $[M+H]^+$; **HRMS** (ESI): Exact mass calcd. for $C_{25}H_{24}BrN_2$ $[M+H]^+$ 431.1123, Found: 431.1131.

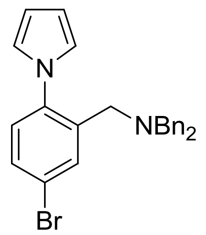

3i

3i, 78% yield, light yellow solid (R_f = 0.85, petroleum ether/ethyl acetate, 50/3), m.p. 99.8-101.5 ^{o}C

^{1}H NMR (300 MHz, $CDCl_3$) δ 7.99 (d, *J* = 2.2 Hz, 1H), 7.48-7.12 (m, 11H), 7.04 (d, *J* = 8.4 Hz, 1H), 6.74-6.56 (m, 2H), 6.38-6.10 (m, 2H), 3.43 (s, 4H), 3.36 (s, 2H).

^{13}C NMR (75 MHz, $CDCl_3$) δ 139.5, 138.9, 138.0, 132.8, 130.3, 128.7, 128.3, 128.1, 127.1, 122.3, 121.6, 109.3, 58.2, 52.3.

MS (ESI): *m/z* 431.1 $[M+H]^+$; **HRMS** (ESI): Exact mass calcd. for $C_{25}H_{24}BrN_2$ $[M+H]^+$ 431.1123, Found: 431.1128.

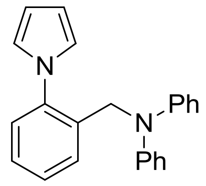

3j

3j, 87% yield, light yellow oil (R_f = 0.87, petroleum ether/ethyl acetate, 50/3).

^{1}H NMR (300 MHz, $CDCl_3$) δ 7.57 (d, *J* = 4.3 Hz, 1H), 7.37-7.12 (m, 7H), 7.04-6.84 (m, 6H), 6.78 (s, 2H), 6.33 (s, 2H), 4.80 (s, 2H).

^{13}C NMR (75 MHz, $CDCl_3$) δ 147.8, 139.5, 135.1, 129.2, 128.1, 127.8, 127.5, 127.1, 122.1, 121.5, 120.6, 109.3, 52.2.

MS (ESI): *m/z* 347.2 $[M+Na]^+$; **HRMS** (ESI): Exact mass calcd. for $C_{23}H_{20}N_2Na$ $[M+Na]^+$ 347.1524, Found: 347.1531.

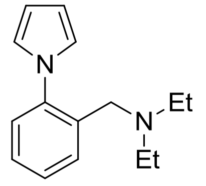

3k

3k, 81% yield, light yellow oil (R_f = 0.35, petroleum ether/ethyl acetate, 50/3).

^{1}H NMR (300 MHz, $CDCl_3$) δ 7.66 (d, *J* = 7.6 Hz, 1H), 7.45-7.12 (m, 3H), 6.84 (t, *J* = 1.8 Hz, 2H), 6.29 (t, *J* = 1.8 Hz, 2H), 3.39 (s, 2H), 2.44 (q, *J* = 7.1 Hz, 4H), 0.94 (t, *J* = 7.1 Hz, 6H).

^{13}C NMR (75 MHz, $CDCl_3$) δ 140.6, 136.1, 130.5, 127.4, 127.1, 126.6, 122.7, 108.6, 52.5, 46.8, 11.7.

MS (ESI): *m/z* 229.2 $[M+H]^+$; **HRMS** (ESI): Exact mass calcd. for $C_{15}H_{21}N_2$ $[M+H]^+$ 229.1705, Found: 229.1699

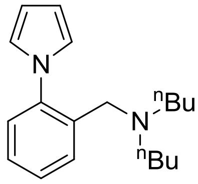

3l

3l, 82% yield, light yellow oil (R_f = 0.50, petroleum ether/ethyl acetate, 50/3).
^{1}H NMR (300 MHz, $CDCl_3$) δ 7.66 (d, *J* = 7.4 Hz, 1H), 7.42-7.11 (m, 3H), 6.82 (t, *J* = 1.8 Hz, 2H), 6.29 (t, *J* = 1.8 Hz, 2H), 3.37 (s, 2H), 2.32 (t, *J* = 7.1 Hz, 4H), 1.47-1.10 (m, 8H), 0.85 (t, *J* = 7.1 Hz, 6H).
^{13}C NMR (75 MHz, $CDCl_3$) δ 140.5, 136.3, 130.5, 127.4, 127.0, 126.5, 122.6, 108.6, 53.6, 29.1, 20.6, 14.0.
HRMS MS (ESI): *m/z* 285.2 $[M+H]^+$; **HRMS** (ESI): Exact mass calcd. for $C_{19}H_{29}N_2$ $[M+H]^+$ 285.2331, Found: 285.2339.

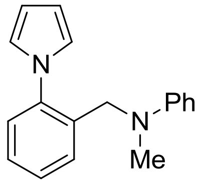

3m

3m, 89% yield, light yellow oil (R_f = 0.75, petroleum ether/ethyl acetate, 50/3).
^{1}H NMR (300 MHz, $CDCl_3$) δ 7.30 (s, 4H), 7.23-7.11 (m, 2H), 6.81 (t, *J* = 2.0 Hz, 2H), 6.72 -6.63 (m, 3H), 6.33 (t, *J* = 2.0 Hz, 2H), 4.35 (s, 2H), 2.94 (s, 3H).
^{13}C NMR (75 MHz, $CDCl_3$) δ 149.5, 139.8, 134.9, 129.1, 128.0, 127.6, 127.5, 127.0, 122.1, 116.6, 112.1, 109.2, 52.6, 38.5.
MS (ESI): *m/z* 285.1 $[M+Na]^+$; **HRMS** (ESI): Exact mass calcd. for $C_{18}H_{18}N_2Na$ $[M+Na]^+$ 285.1368, Found: 285.1376.

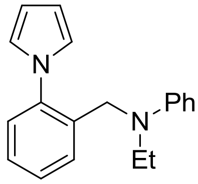

3n

3n, 88% yield, light yellow oil (R_f = 0.75, petroleum ether/ethyl acetate, 50/3).
^{1}H NMR (300 MHz, $CDCl_3$) δ 7.43-7.24 (m, 4H), 7.16 (t, *J* = 8.0 Hz, 2H), 6.83 (t, *J* = 1.9 Hz, 2H), 6.68-6.59 (m, 3H), 6.34 (t, *J* = 1.9 Hz, 2H), 4.33 (s, 2H), 3.40 (q, *J* = 7.0 Hz, 2H), 1.13 (t, *J* = 7.0 Hz, 3H).
^{13}C NMR (75 MHz, $CDCl_3$) δ 148.2, 139.8, 135.2, 129.2, 128.0, 127.6, 127.4, 127.0, 122.1, 116.2, 112.0, 109.2, 50.0, 45.2, 12.1.
MS (ESI): *m/z* 299.2 $[M+Na]^+$; **HRMS** (ESI): Exact mass calcd. for $C_{19}H_{20}N_2Na$ $[M+Na]^+$ 299.1524, Found: 299.1531.

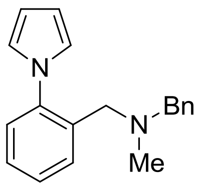

3o

3o, 94% yield, light yellow oil (R_f = 0.70, petroleum ether/ethyl acetate, 50/3).
^{1}H NMR (300 MHz, $CDCl_3$) δ 7.67 (d, *J* = 7.3 Hz, 1H), 7.46-7.13 (m, 8H), 6.88 (t, *J* = 2.1 Hz, 2H), 6.31 (t, *J* = 2.0 Hz, 2H), 3.44 (s, 2H), 3.37 (s, 2H), 2.07 (s, 3H).
^{13}C NMR (75 MHz, $CDCl_3$) δ 140. 8, 139.3, 135.0, 130.6, 128.8, 128.2, 127.5, 127.4, 126. 9, 126.6, 122. 7, 108.7, 61.8, 56.8, 41.9.
MS (ESI): *m/z* 299.2 $[M+Na]^+$; **HRMS** (ESI): Exact mass calcd. for $C_{19}H_{20}N_2Na$ $[M+Na]^+$ 299.1524, Found: 299.1520.

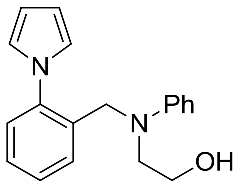

3p

3p, 88% yield, light yellow oil (R_f = 0.40, petroleum ether/ethyl acetate, 50/3).
^{1}H NMR (300 MHz, $CDCl_3$) δ 7.29-7.26 (m, 4H), 7.19-7.13 (m, 2H), 6.81 ((t, *J* = 2.1 Hz, 2H), 6.72-6.86 (m, 3H), 6.34 (t, *J* = 2.1 Hz, 2H), 4.41 (s, 2H), 3.73 (t, *J* = 5.7 Hz, 2H), 3.51 (t, *J* = 5.8 Hz, 2H).
^{13}C NMR (75 MHz, $CDCl_3$) δ 148.4, 139.9, 134.6, 129.3, 128.1, 127.6, 127.5, 127.2, 122.1, 117.2, 112.8, 109.3, 60.1, 53.4, 51.1.
MS (ESI): *m/z* 315.1 $[M+Na]^+$; **HRMS** (ESI): Exact mass calcd. for $C_{19}H_{20}N_2ONa$ $[M+Na]^+$ 315.1473, Found: 315.1467.

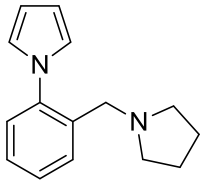

3q

3p, 77% yield, light yellow oil (R_f = 0.45, petroleum ether/ethyl acetate, 50/3).
^{1}H NMR (300 MHz, $CDCl_3$) δ 7.55 (d, *J* = 6.7 Hz, 1H), 7.38-7.15 (m, 3H), 6.94 (d, *J* = 0.9 Hz, 2H), 6.30 (d, *J* = 0.9 Hz, 2H), 3.45 (s, 2H), 2.45 (s, 4H), 1.74 (s, 4H).
^{13}C NMR (75 MHz, $CDCl_3$) δ 140.4, 135.0, 130.6, 127.4, 127.2, 126.4, 122.8, 108.7, 55.2, 53.9, 23.5.
MS (ESI): *m/z* 227.2 $[M+H]^+$; **HRMS** (ESI): Exact mass calcd. for $C_{15}H_{19}N_2$ $[M+H]^+$ 227.1548, Found: 227.1553.

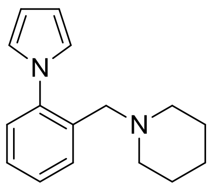

3r

3r, 84% yield, light yellow oil (R_f = 0.45, petroleum ether/ethyl acetate, 50/3).

^{1}H NMR (500 MHz, $CDCl_3$) δ 7.54 (d, *J* = 7.1 Hz, 1H), 7.39-7.13 (m, 3H), 6.93 (t, *J* = 2.0 Hz, 2H), 6.297 (d, *J* = 2.0 Hz, 2H), 3.26 (s, 2H), 2.31 (s, 4H), 1.55-1.51 (m, 4H), 1.40 (d, *J* = 4.4 Hz, 2H).

^{13}C NMR (75 MHz, $CDCl_3$) δ 141.0, 134.4, 130.9, 127.4, 127.1, 126.5, 122.8, 108.6, 58.4, 54.3, 26.1, 24.3.

MS (ESI): *m/z* 241.2 $[M+H]^+$; **HRMS** (ESI): Exact mass calcd. for $C_{16}H_{21}N_2$ $[M+H]^+$ 241.1705, Found: 241.1713.

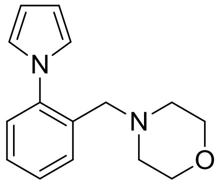

3s

3s, 98% yield, light yellow oil (R_f = 0.40, petroleum ether/ethyl acetate, 50/3).

^{1}H NMR (300 MHz, $CDCl_3$) δ 7.50 (dd, *J* = 4.9, 4.0 Hz, 1H), 7.30 (dt, *J* = 10.2, 3.4 Hz, 3H), 6.94-6.93 (m, 2H), 6.30-6.28 (m, 2H), 3.66 (t, *J* = 4.5 Hz, 4H), 3.30 (s, 2H), 2.38 (t, *J* = 4.4 Hz, 4H).

^{13}C NMR (75 MHz, $CDCl_3$) δ 141.1, 133.2, 131.0, 127.8, 127.0, 126.7, 122.7, 108.7, 67.0, 58.1, 53.2.

MS (ESI): *m/z* 265.1 $[M+Na]^+$; **HRMS** (ESI): Exact mass calcd. for $C_{15}H_{18}N_2ONa$ $[M+Na]^+$ 265.1317, Found: 265.1314.

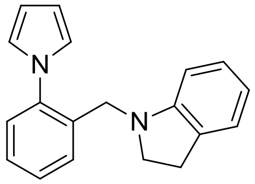

3t

3t, 84% yield, light yellow oil (R_f = 0.85, petroleum ether/ethyl acetate, 50/3).

^{1}H NMR (300 MHz, $CDCl_3$) δ 7.55 (dd, *J* = 5.8, 2.9 Hz, 1H), 7.37-7.24 (m, 3H), 7.06 (d, *J* = 7.2 Hz, 1H), 7.00 (t, *J* = 7.7 Hz, 1H), 6.85 (t, *J* = 2.1 Hz, 2H), 6.65 (t, *J* = 7.3 Hz, 1H), 6.35 (d, *J* = 7.8 Hz, 1H), 6.29 (t, *J* = 2.1 Hz, 2H), 4.05 (s, 2H), 3.24 (t, *J* = 8.3 Hz, 2H), 2.92 (t, *J* = 8.2 Hz, 2H).

^{13}C NMR (75 MHz, $CDCl_3$) δ 152.2, 140.3, 134.1, 129.9, 129.6, 127.8, 127.7, 127.2, 126.9, 124.4, 122.4, 117.9, 109.1, 107.1, 53.8, 49.5, 28.5.

MS (ESI): *m/z* 297.1 $[M+Na]^+$; **HRMS** (ESI): Exact mass calcd. for $C_{19}H_{18}N_2Na$ $[M+Na]^+$ 297.1368, Found: 297.1374.

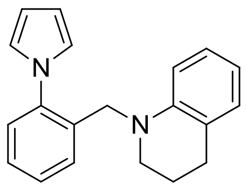

3u

3u, 88% yield, light yellow oil (R_f = 0.90, petroleum ether/ethyl acetate, 50/3).
^{1}H NMR (300 MHz, $CDCl_3$) δ 7.43-7.34 (m, 1H), 7.29 (m, 3H), 6.94 (t, *J* = 7.1 Hz, 2H), 6.82 (t, *J* = 2.1 Hz, 2H), 6.56 (t, *J* = 7.3 Hz, 1H), 6.36 (s, 1H), 6.33 (t, *J* = 2.0 Hz, 2H), 4.29 (s, 3H), 3.26 (t, *J* = 5.7 Hz, 3H), 2.78 (t, *J* = 6.2 Hz, 3H), 2.05-1.90 (m, 3H).
^{13}C NMR (75 MHz, $CDCl_3$) δ 145.4, 139.9, 134.7, 129.0, 128.0, 127.4, 127.1, 127.06, 122.2, 122.1, 116.0, 110.7, 109.2, 51.4, 49. 9, 28.1, 22.3.
MS (ESI): *m/z* 311.2 $[M+Na]^+$; **HRMS** (ESI): Exact mass calcd. for $C_{20}H_{20}N_2$ $[M+Na]^+$ 311.1524, Found: 311.1534.

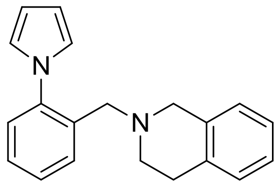

3v

3v, 76% yield, light yellow oil (R_f = 0.70, petroleum ether/ethyl acetate, 50/3).
^{1}H NMR (300 MHz, $CDCl_3$) δ 7.71–7.53 (m, 1H), 7.38-7.21 (m, 3H), 7.13-7.03 (m, 3H), 6.97-6.94 (m, 3H), 6.31-6.29 (m, 2H), 3.58 (s, 2H), 3.48 (s, 2H), 2.87 (t, *J* = 5.7 Hz, 2H), 2.68 (t, *J* = 5.8 Hz, 2H).
^{13}C NMR (75 MHz, $CDCl_3$) δ 141.0, 134.9, 134.4, 134.0, 130.8, 128.6, 127.7, 127.3, 126.7, 126.5, 126.0, 125.5, 122.8, 108.8, 57.6, 55.8, 50.4, 29.4.
MS (ESI): *m/z* 311.2 $[M+Na]^+$; **HRMS** (ESI): Exact mass calcd. for $C_{20}H_{20}N_2Na$ $[M+Na]^+$ 311.1524, Found: 311.1516.

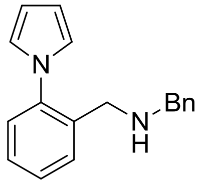

3w

3w, 98% yield, light yellow oil (R_f = 0.35, petroleum ether/ethyl acetate, 50/3).
^{1}H NMR (300 MHz, $CDCl_3$) δ 7.53-7.41 (m, 1H), 7.36-7.09 (m, 8H), 6.85 (t, *J* = 1.9 Hz, 2H), 6.29 (t, *J* = 1.9 Hz, 2H), 3.68 (s, 2H), 3.65 (s, 2H).
^{13}C NMR (75 MHz, $CDCl_3$) δ 140.3, 140.0, 135.7, 130.0, 128.2, 128.0, 127.7, 127.5, 126.8, 126.6, 122.2, 109.0, 53.3, 49.2.
MS (ESI): *m/z* 285.1 $[M+Na]^+$; **HRMS** (ESI): Exact mass calcd. for $C_{18}H_{18}N_2Na$ $[M+Na]^+$ 285.1368, Found: 285.1376.

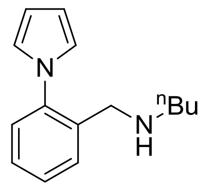

3x

3x, 33% yield, light yellow oil (R_f = 0.35, petroleum ether/ethyl acetate, 50/3).
^{1}H NMR (300 MHz, d_6-DMSO) δ 7.59-7.44 (m, 1H), 7.38-7.19 (m, 3H), 6.93-6.77 (m, 2H), 6.39-6.23 (m, 2H), 3.66 (s, 2H), 2.49 (t, *J* = 6.9 Hz, 2H), 1.45-1.33 (m, 2H), 1.27 (dt, *J* = 13.8, 6.7 Hz, 2H), 0.87 (t, *J* = 7.1 Hz, 3H).
^{13}C NMR (75 MHz, $CDCl_3$) δ 140.2, 136.1, 129.9, 127.6, 126.7, 122.3, 109.0, 49.8, 49.2, 32.1, 20.4, 13.9.
MS (ESI): *m/z* 251.2 $[M+Na]^+$; **HRMS** (ESI): Exact mass calcd. for $C_{15}H_{20}N_2Na$ $[M+Na]^+$ 251.1524, Found: 251.1530.

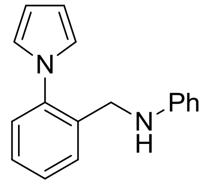

3y

3y, 99% yield, light yellow solid (R_f = 0.60, petroleum ether/ethyl acetate, 50/3), m.p. 81.5-84.0 °C.
^{1}H NMR (500 MHz, $CDCl_3$) δ 7.52-7.42 (m, 1H), 7.36-7.22 (m, 3H), 7.10 (t, *J* = 7.8 Hz, 2H), 6.84-6.83 (m, 2H), 6.66 (t, *J* = 7.2 Hz, 1H), 6.49 (d, *J* = 8.1 Hz, 2H), 6.33-6.31 (m, 2H), 4.18 (s, 2H), 3.74 (s, 1H).
^{13}C NMR (75 MHz, $CDCl_3$) δ 147.6, 140.1, 134.8, 129.4, 129.1, 128.0, 127.8, 126.8, 122.1, 117.6, 112.93, 109.4, 44.3.
MS (ESI): *m/z* 249.1 $[M+H]^+$; **HRMS** (ESI): Exact mass calcd. for $C_{17}H_{17}N_2$ $[M+H]^+$ 249.1392, Found: 249.1385.

References:

[1] Han, Y.-Y.; Han, W.-Y.; Hou, X.; Zhang, X.-M.; Yuan, W.-C. *Org. Lett.* **2012**, *14*, 4054.

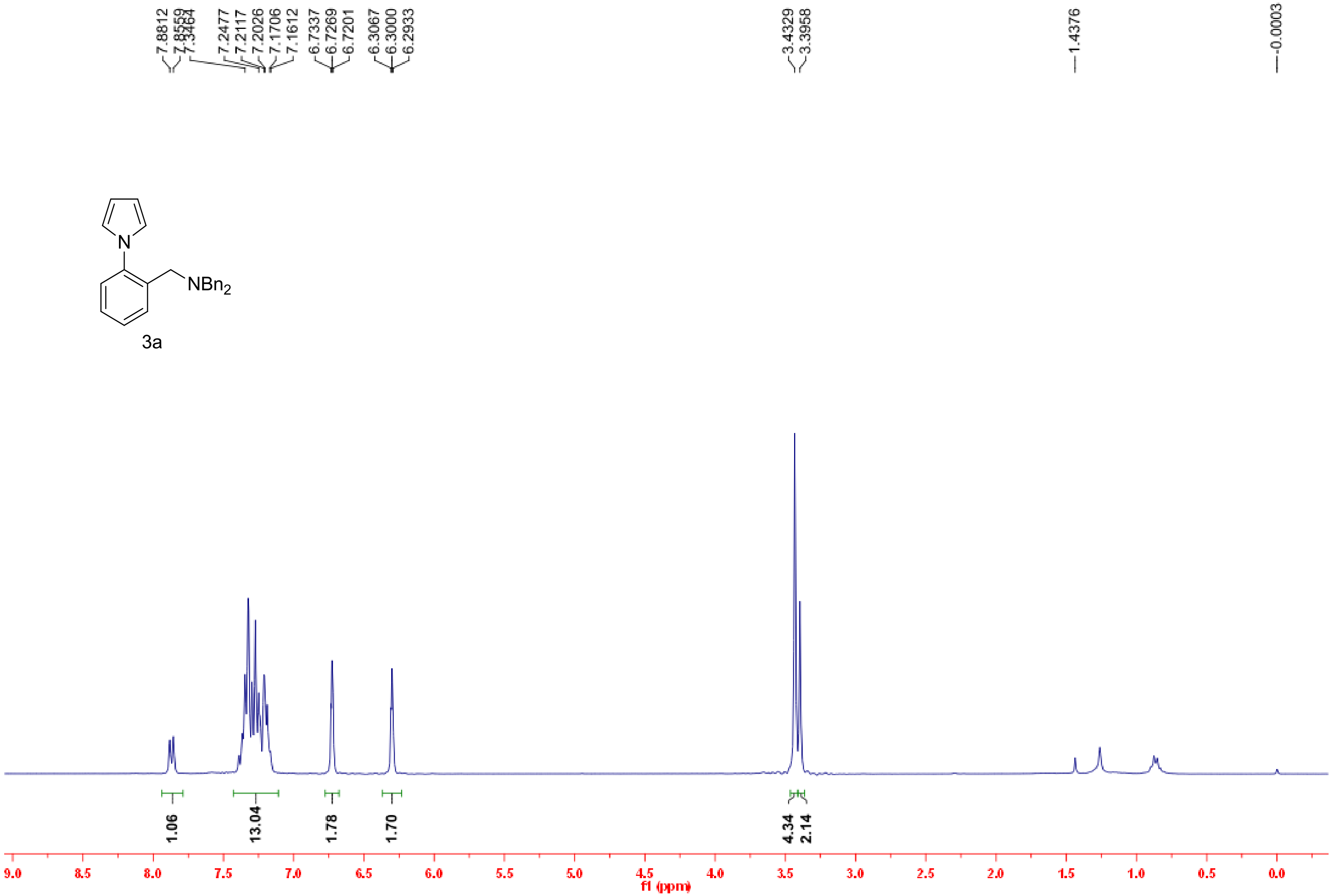
N
NBn2
3a
7.8812
7.8559
7.3464
7.2477
7.2117
7.2026
7.1706
7.1612
6.7337
6.7269
6.7201
6.3067
6.3000
6.2933
3.4329
3.3958
1.4376
-0.0003
1.06
13.04
1.78
1.70
4.34
2.14
9.0
8.5
8.0
7.5
7.0
6.5
6.0
5.5
5.0
4.5
4.0
3.5
3.0
2.5
2.0
1.5
1.0
0.5
0.0
f1 (ppm)

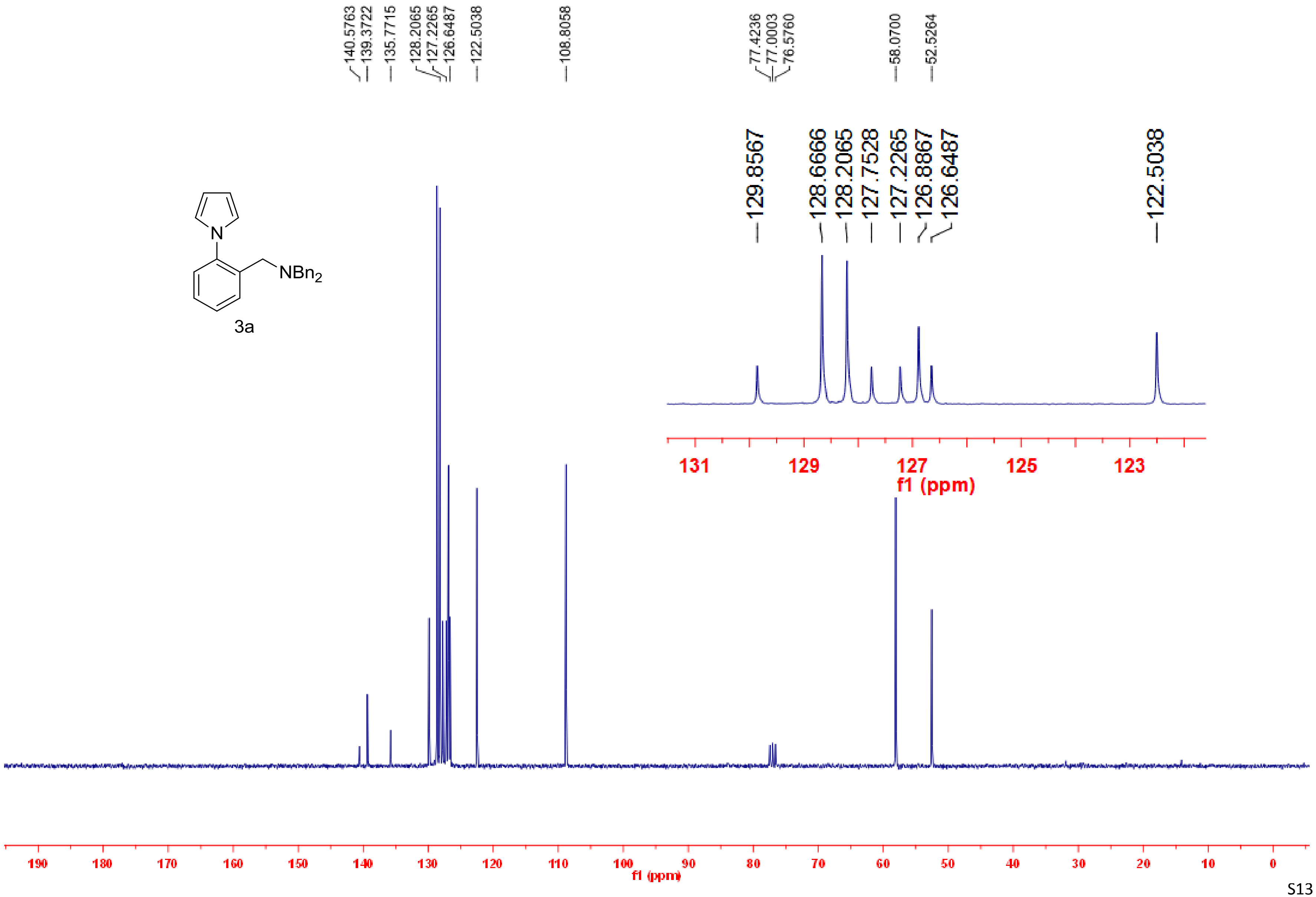
140.5763
139.3722
135.7715
128.2065
127.2265
126.6487
122.5038
108.8058
77.4236
77.0003
76.5760
58.0700
52.5264
129.8567
128.6666
128.2065
127.7528
127.2265
126.8867
126.6487
122.5038
NBn2
3a
f1 (ppm)

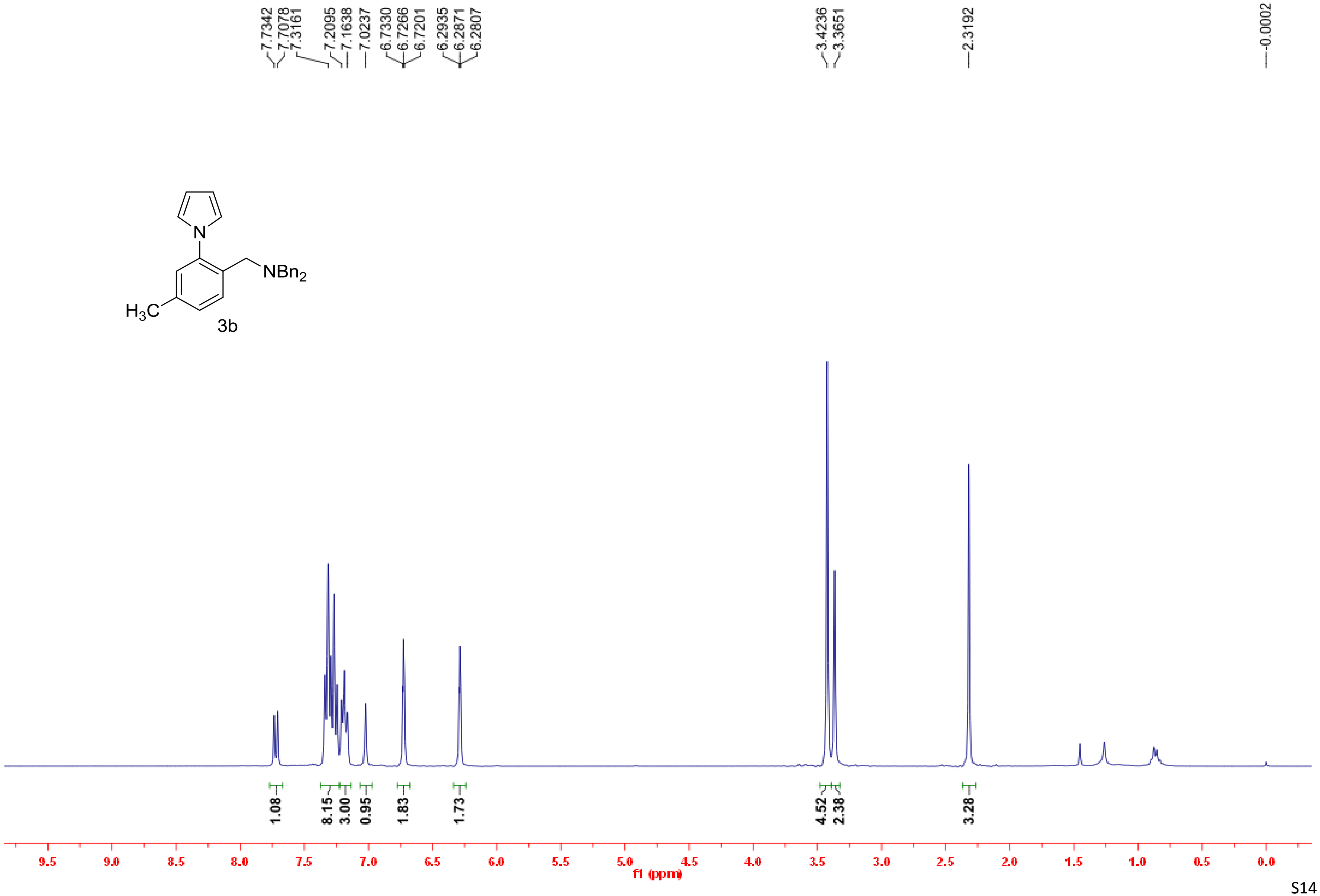
N
NBn$_2$
H$_3$C
3b
7.7342
7.7078
7.3161
7.2095
7.1638
7.0237
6.7330
6.7266
6.7201
6.2935
6.2871
6.2807
3.4236
3.3651
2.3192
0.0002
1.08
8.15
3.00
0.95
1.83
1.73
4.52
2.38
3.28
9.5
9.0
8.5
8.0
7.5
7.0
6.5
6.0
5.5
5.0
4.5
4.0
3.5
3.0
2.5
2.0
1.5
1.0
0.5
0.0
f1 (ppm)

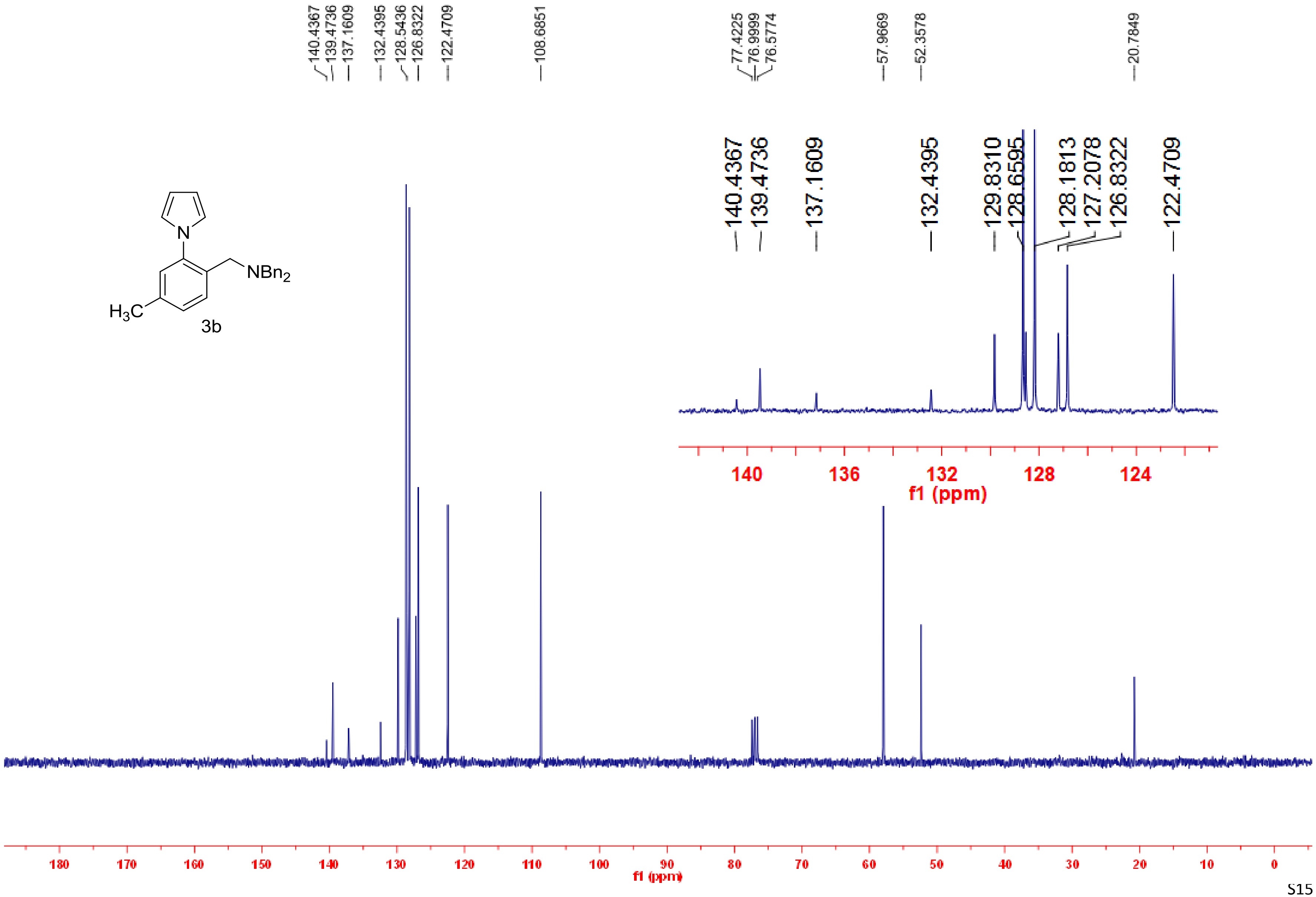
140.4367
139.4736
137.1609
132.4395
128.5436
126.8322
122.4709
108.6851
77.4225
76.9999
76.5774
57.9669
52.3578
20.7849
NBn2
H3C
N
3b
140.4367
139.4736
137.1609
132.4395
129.8310
128.6595
128.1813
127.2078
126.8322
122.4709
140
136
132
128
124
f1 (ppm)
180
170
160
150
140
130
120
110
100
90
80
70
60
50
40
30
20
10
0
f1 (ppm)

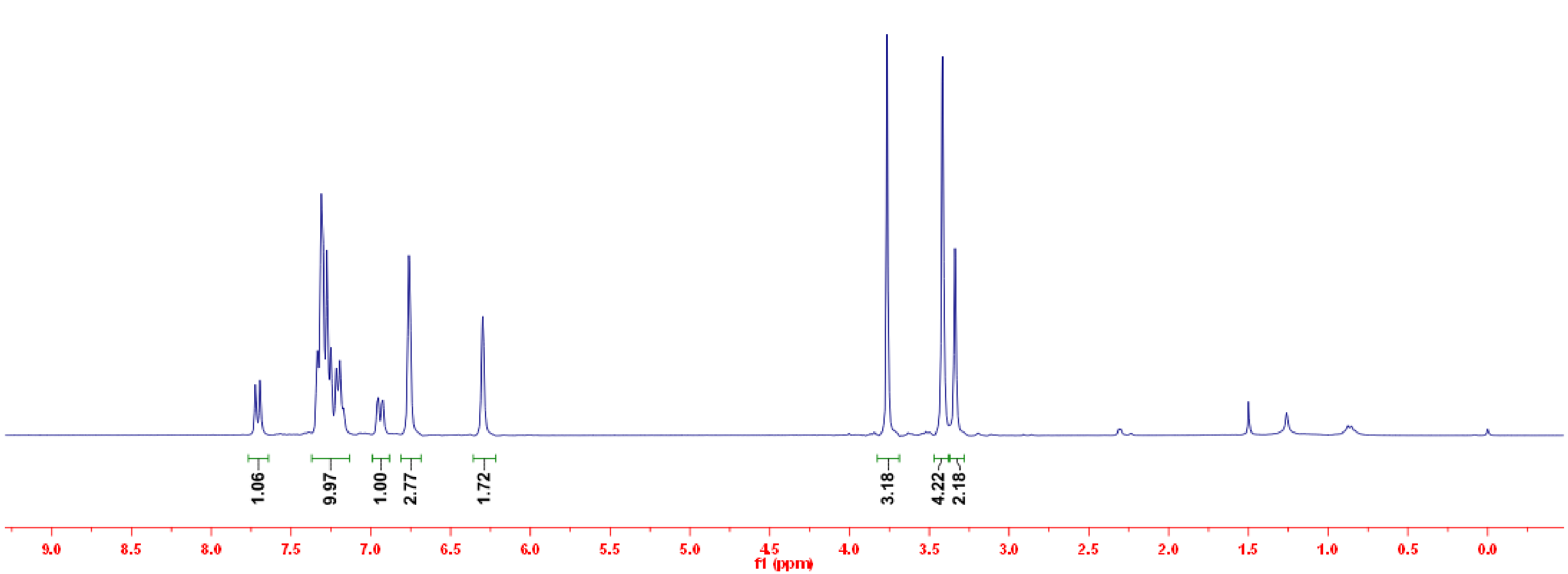
7.7239
7.6952
7.2752
7.2164
7.1717
6.9243
6.7610
6.2991
3.7650
3.4167
3.3388
0.0005
1.06
9.97
1.00
2.77
1.72
3.18
4.22
2.18
9.0 8.5 8.0 7.5 7.0 6.5 6.0 5.5 5.0 4.5 4.0 3.5 3.0 2.5 2.0 1.5 1.0 0.5 0.0
f1 (ppm)

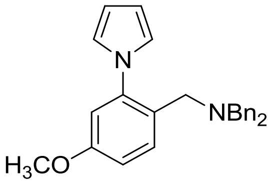
N
NBn2
H3CO

3c

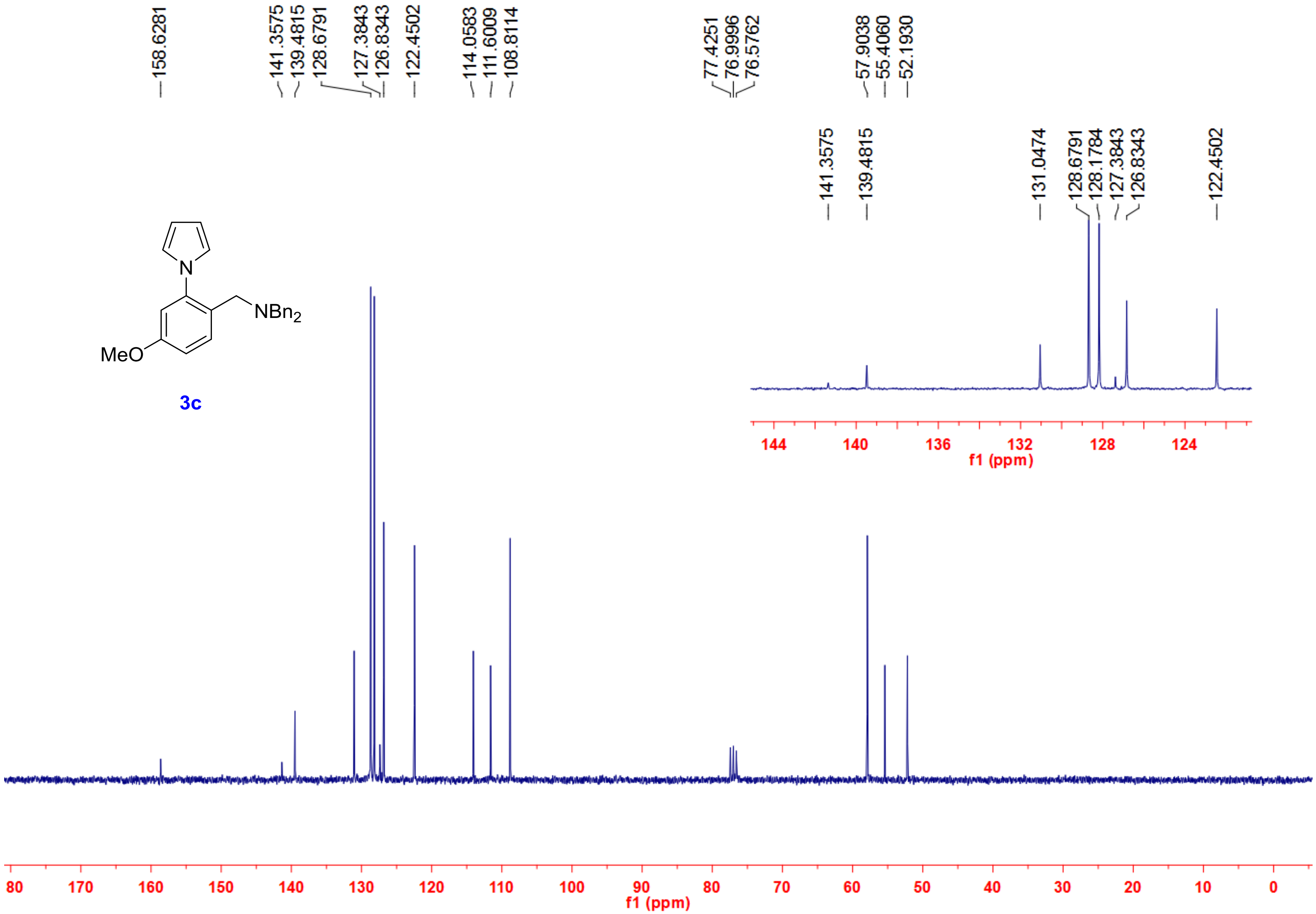

158.6281
141.3575
139.4815
128.6791
127.3843
126.8343
122.4502
114.0583
111.6009
108.8114
77.4251
76.9996
76.5762
57.9038
55.4060
52.1930
141.3575
139.4815
131.0474
128.6791
128.1784
127.3843
126.8343
122.4502
NBn2
MeO
N
3c
144 140 136 132 128 124
f1 (ppm)
80 170 160 150 140 130 120 110 100 90 80 70 60 50 40 30 20 10 0
f1 (ppm)

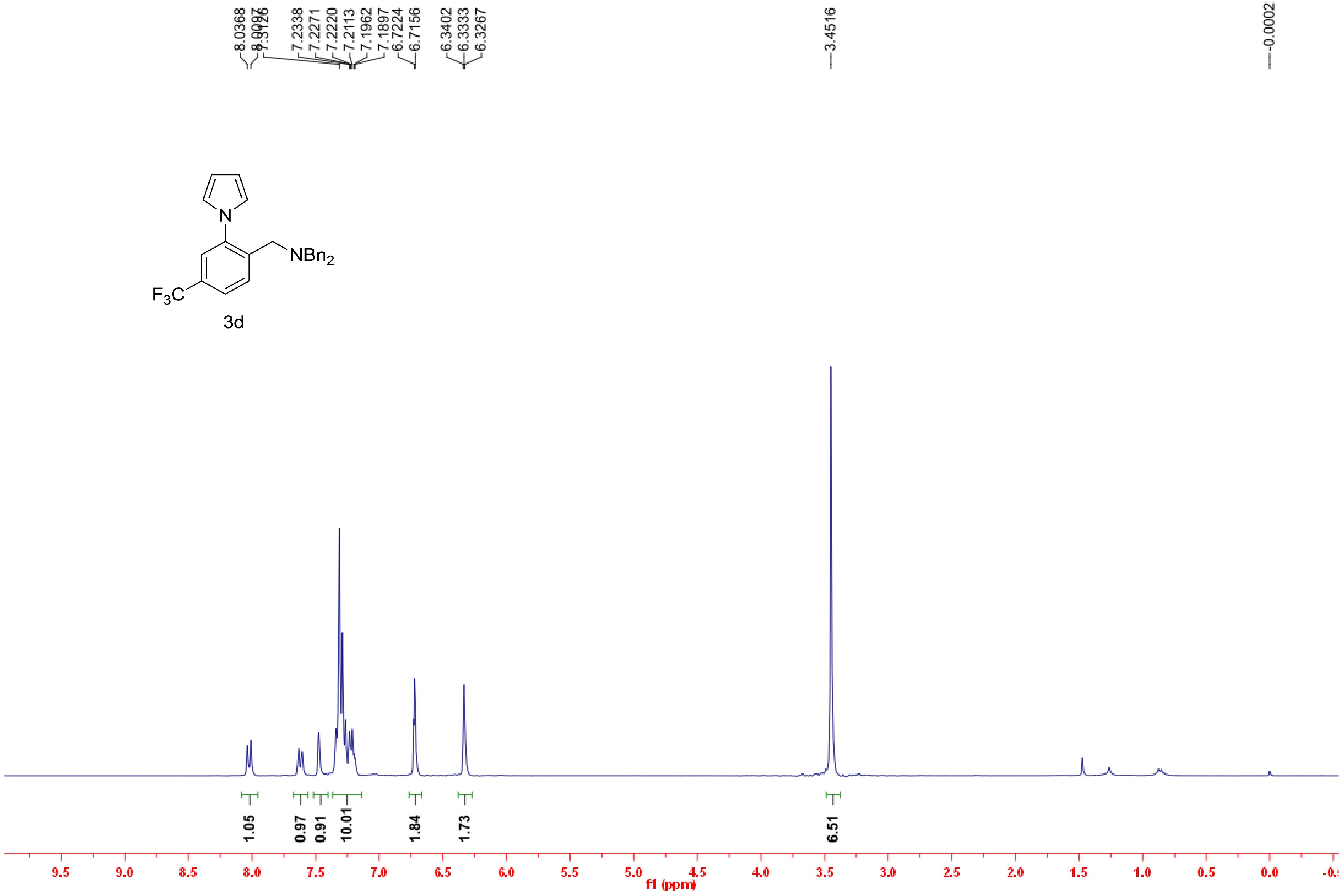

8.0368
7.2338
7.2271
7.2220
7.2113
7.1962
7.1897
6.7224
6.7156
6.3402
6.3333
6.3267
3.4516
-0.0002
NBn2
F3C
N
3d
1.05
0.97
0.91
10.01
1.84
1.73
6.51
9.5
9.0
8.5
8.0
7.5
7.0
6.5
6.0
5.5
5.0
4.5
4.0
3.5
3.0
2.5
2.0
1.5
1.0
0.5
0.0
f1 (ppm)

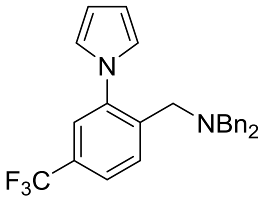
N
NBn2
F3C

3d

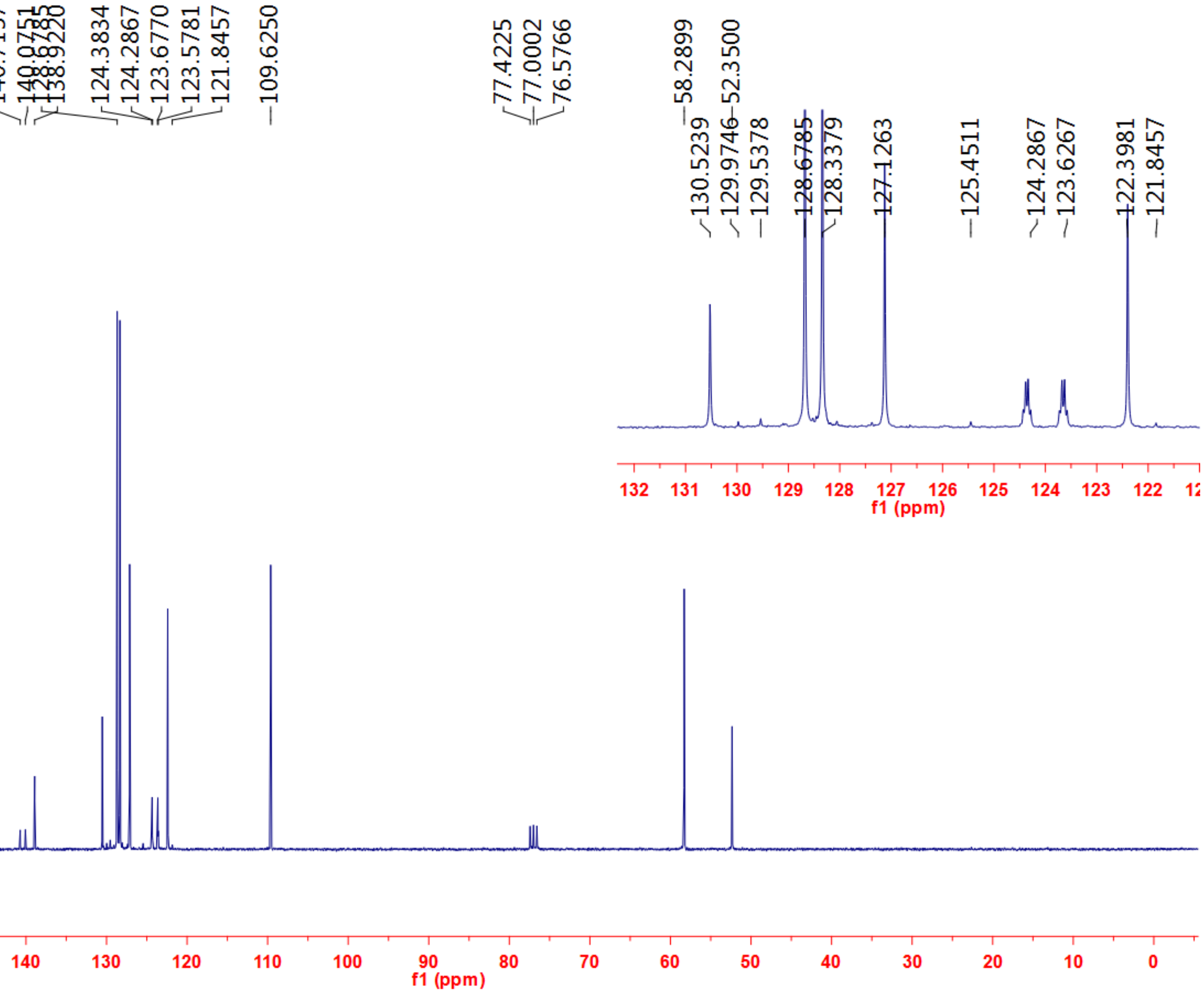
140.7157
140.0751
138.9220
124.3834
124.2867
123.6770
123.5781
121.8457
109.6250
77.4225
77.0002
76.5766
58.2899
52.3500
130.5239
129.9746
129.5378
128.6785
128.3379
127.1263
125.4511
124.2867
123.6267
122.3981
121.8457
f1 (ppm)
f1 (ppm)

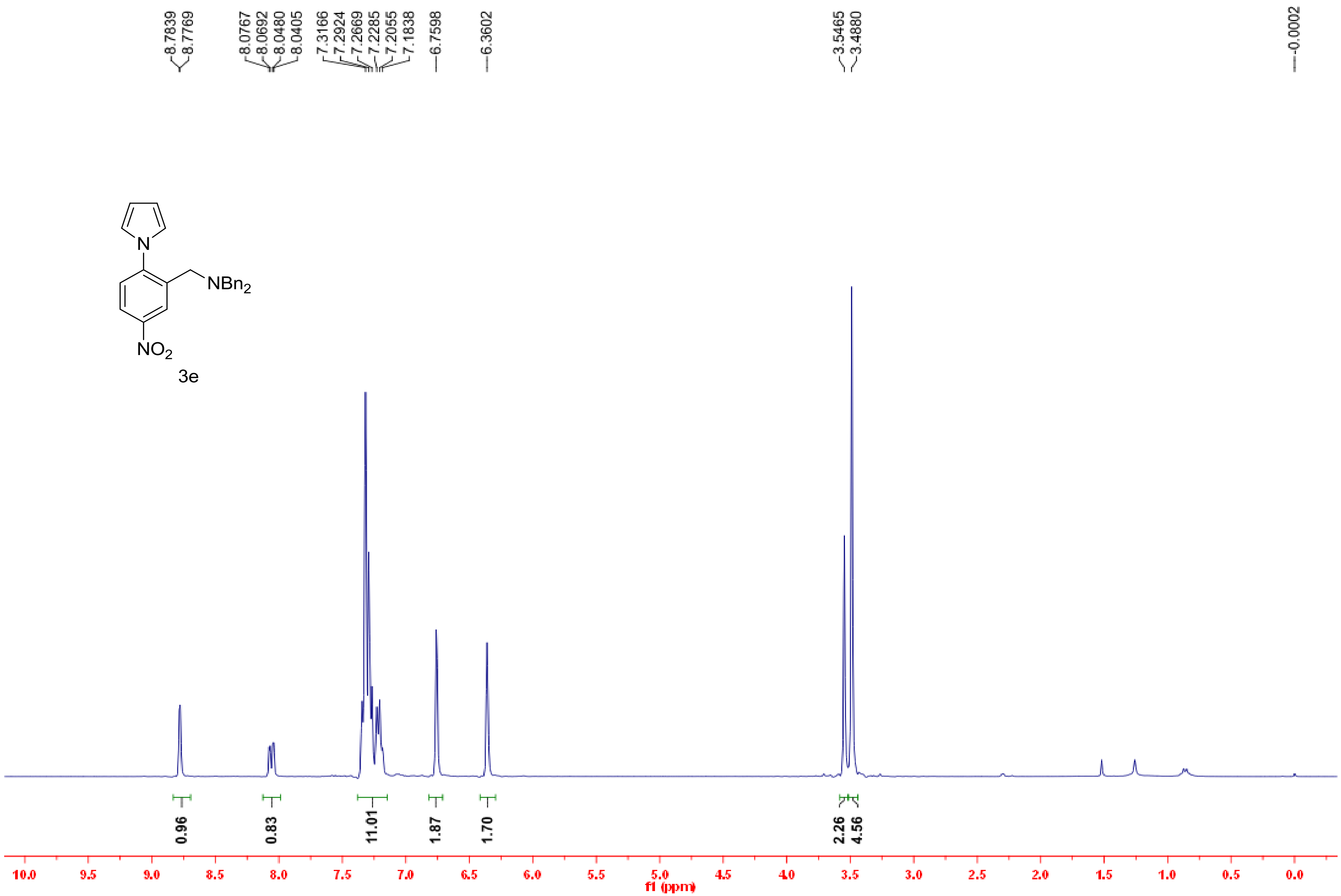

8.7839
8.7769
8.0767
8.0692
8.0480
8.0405
7.3166
7.2924
7.2669
7.2285
7.2055
7.1838
6.7598
6.3602
3.5465
3.4880
-0.0002
NBn2
NO2
3e
0.96
0.83
11.01
1.87
1.70
2.26
4.56
10.0 9.5 9.0 8.5 8.0 7.5 7.0 6.5 6.0 5.5 5.0 4.5 4.0 3.5 3.0 2.5 2.0 1.5 1.0 0.5 0.0
f1 (ppm)

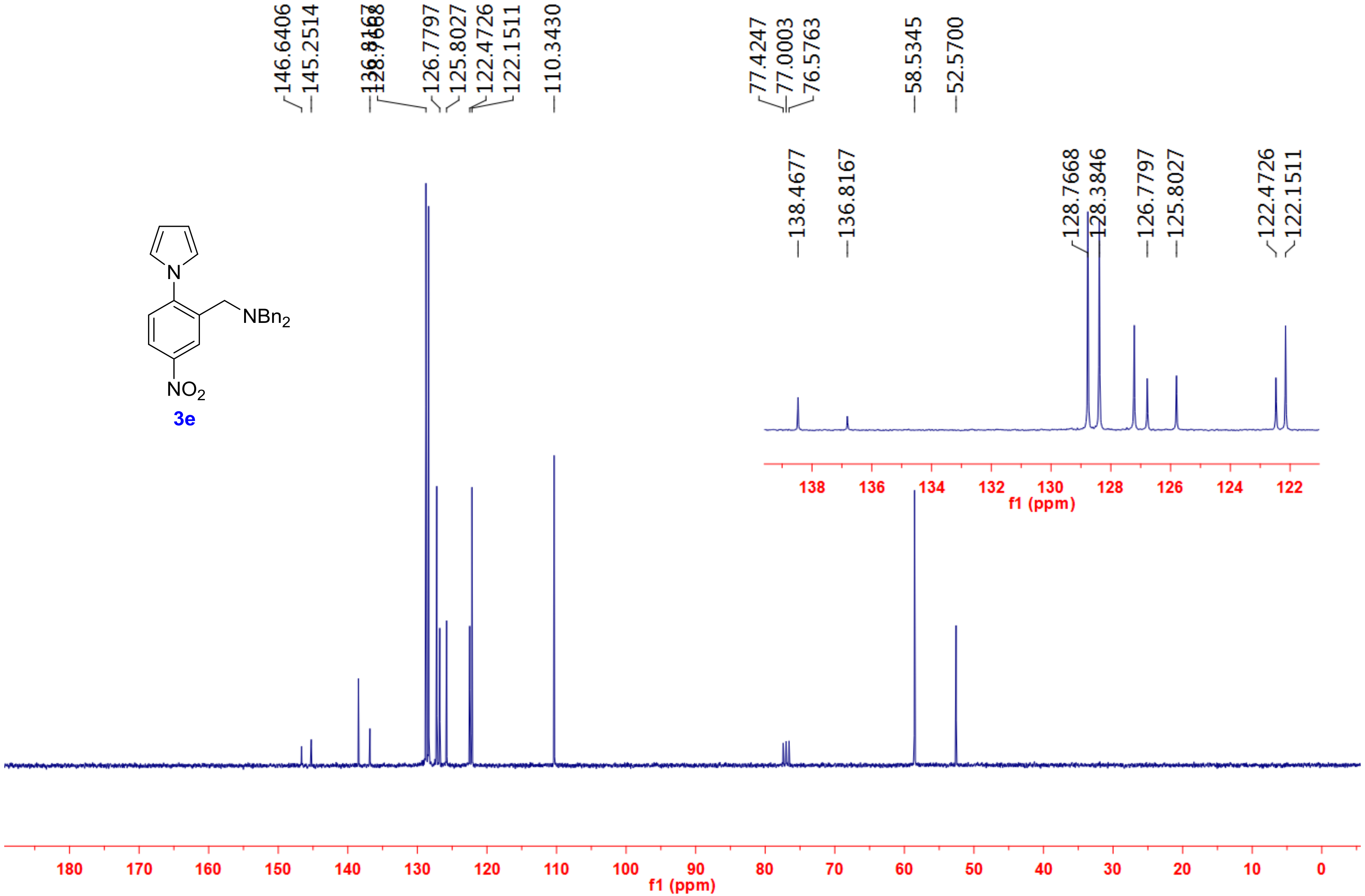
146.6406
145.2514
138.4677
136.8167
128.7668
128.3846
126.7797
125.8027
122.4726
122.1511
110.3430
77.4247
77.0003
76.5763
58.5345
52.5700
f1 (ppm)
NBn2
N
NO2

3e

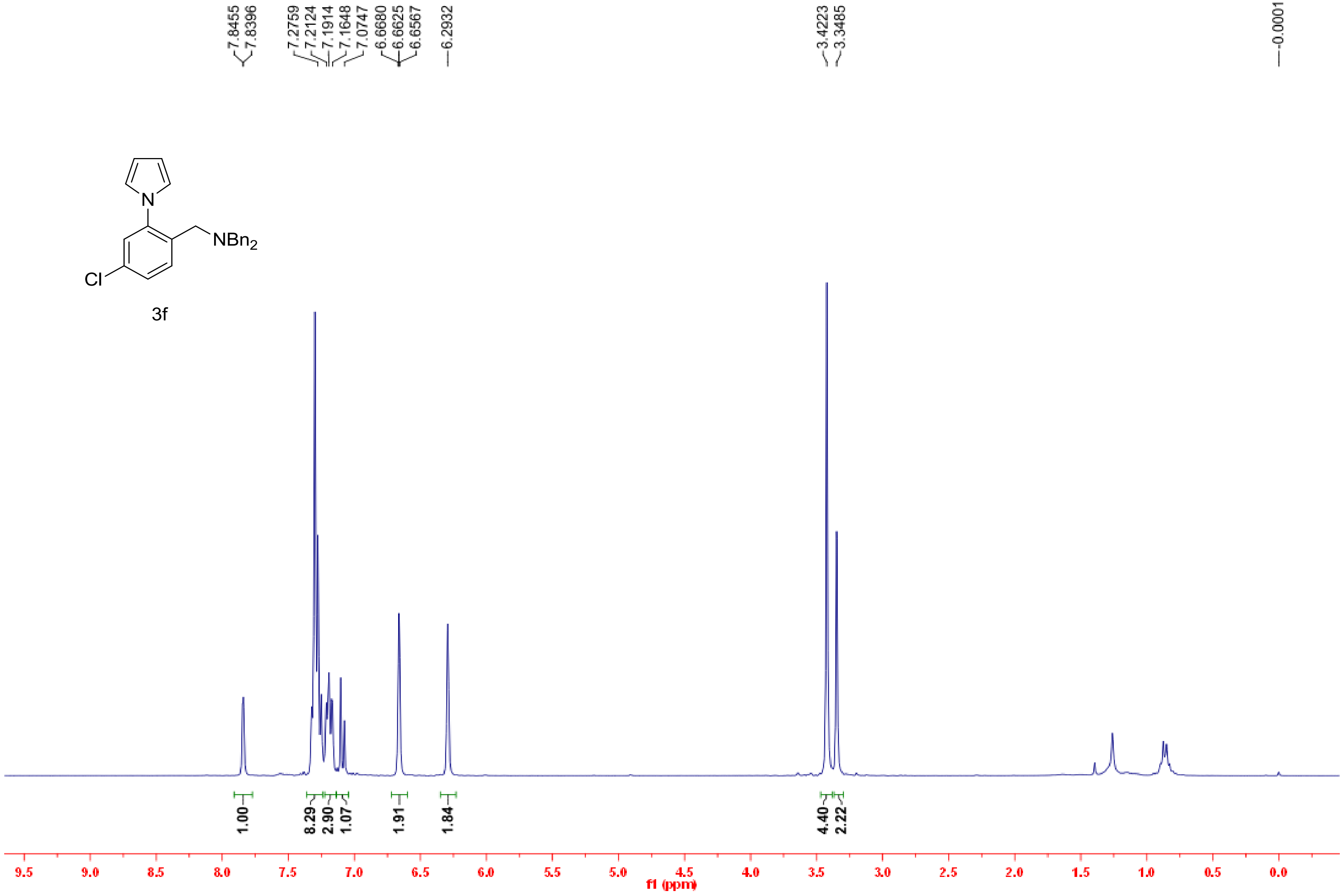
7.8455
7.8396
7.2759
7.2124
7.1914
7.1648
7.0747
6.6680
6.6625
6.6567
6.2932
3.4223
3.3485
-0.0001
N
NBn2
Cl
3f
1.00
8.29
2.90
1.07
1.91
1.84
4.40
2.22
9.5
9.0
8.5
8.0
7.5
7.0
6.5
6.0
5.5
5.0
4.5
4.0
3.5
3.0
2.5
2.0
1.5
1.0
0.5
0.0
f1 (ppm)

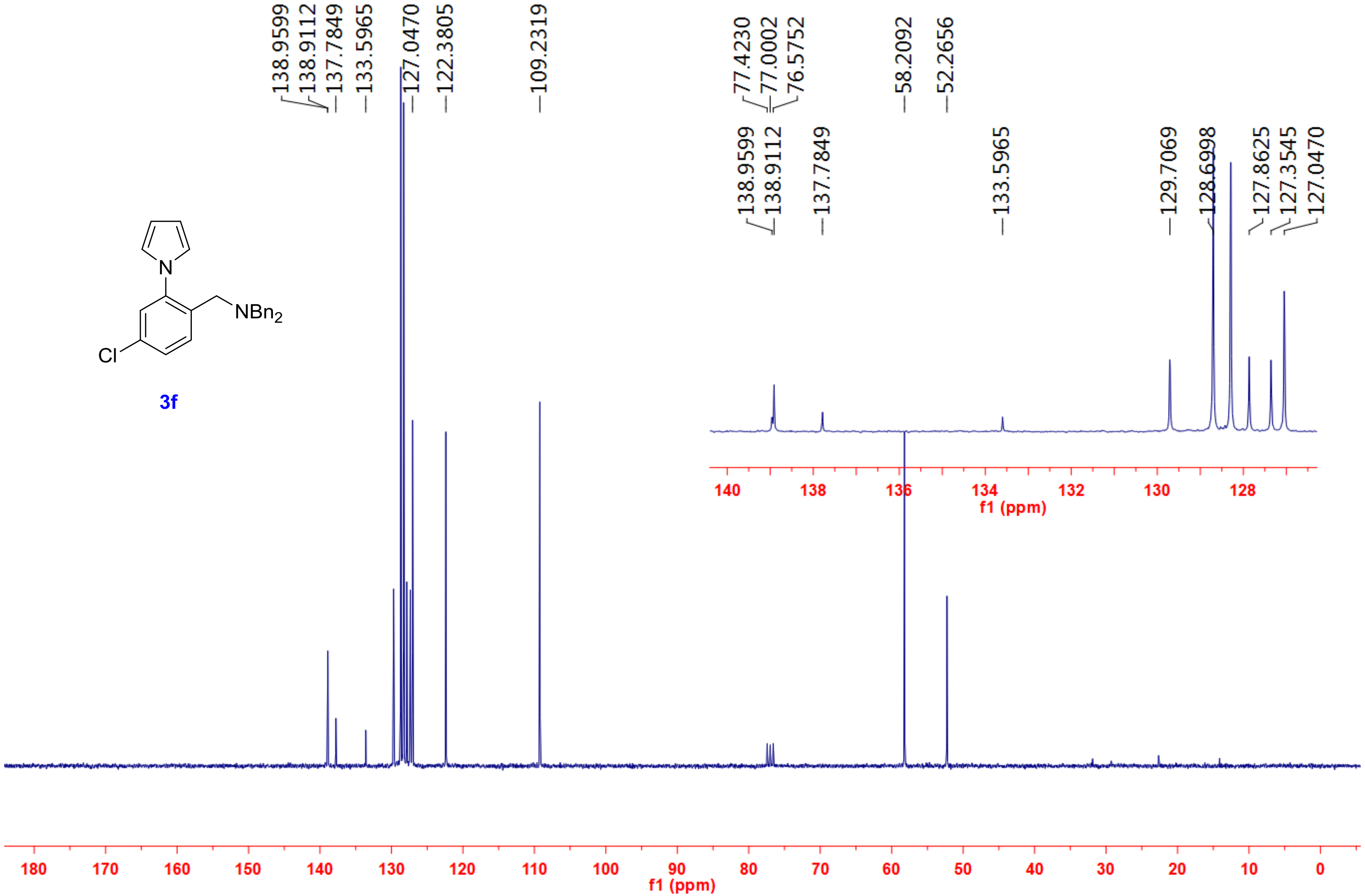

138.9599
138.9112
137.7849
133.5965
127.0470
122.3805
109.2319
77.4230
77.0002
76.5752
58.2092
52.2656
138.9599
138.9112
137.7849
133.5965
129.7069
128.6998
127.8625
127.3545
127.0470
140
138
136
134
132
130
128
f1 (ppm)
N
NBn2
Cl
3f
180
170
160
150
140
130
120
110
100
90
80
70
60
50
40
30
20
10
0
f1 (ppm)

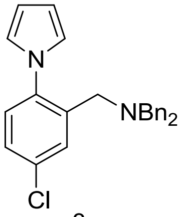
N
NBn2
Cl

3g

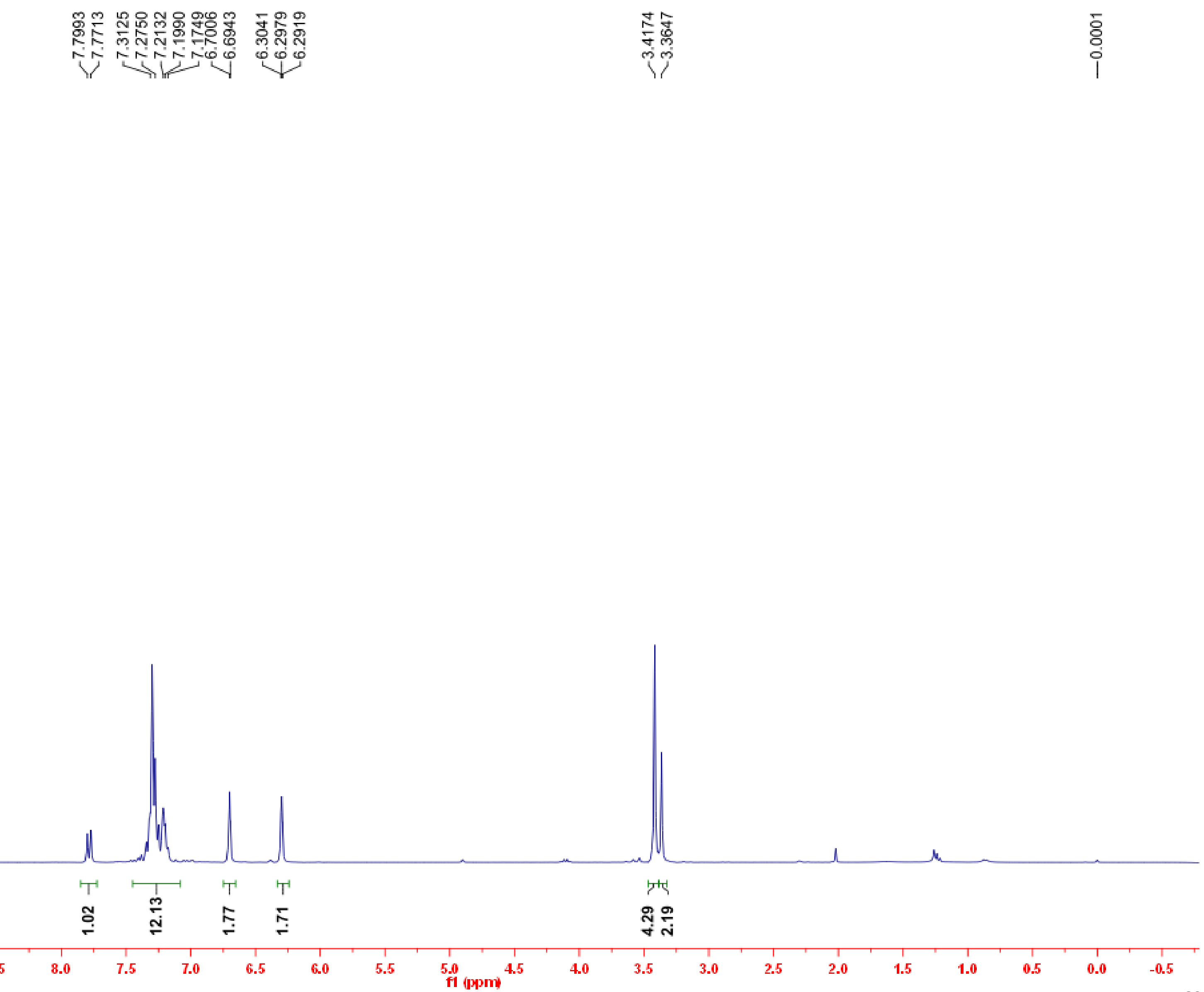
7.7993
7.7713
7.3125
7.2750
7.2132
7.1990
7.1749
6.7006
6.6943
6.3041
6.2979
6.2919
3.4174
3.3647
0.0001
1.02
12.13
1.77
1.71
4.29
2.19
10.0 9.5 9.0 8.5 8.0 7.5 7.0 6.5 6.0 5.5 5.0 4.5 4.0 3.5 3.0 2.5 2.0 1.5 1.0 0.5 0.0 -0.5
f1 (ppm)

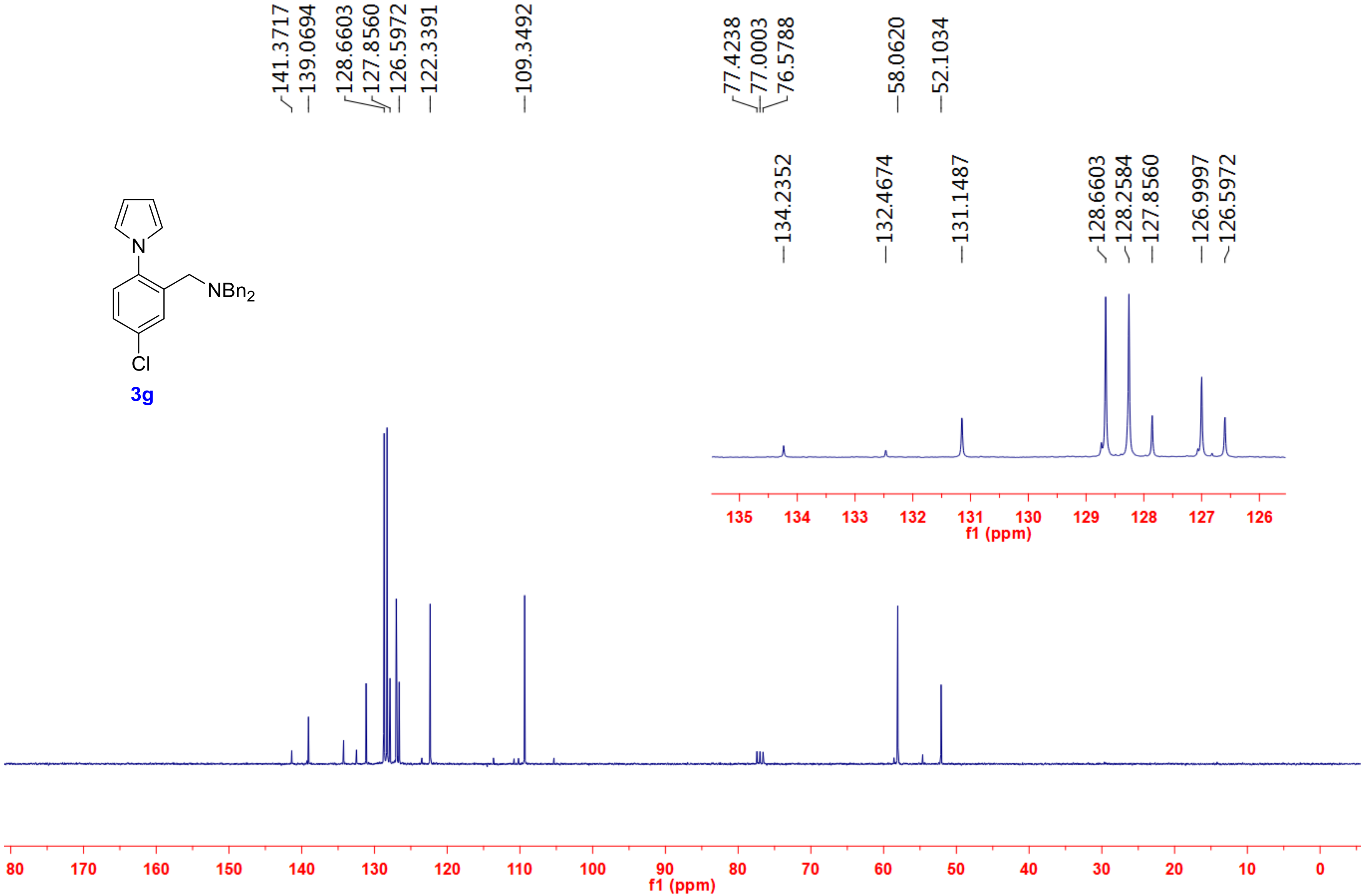
NBn2
Cl
3g
141.3717
139.0694
128.6603
127.8560
126.5972
122.3391
109.3492
77.4238
77.0003
76.5788
58.0620
52.1034
134.2352
132.4674
131.1487
128.6603
128.2584
127.8560
126.9997
126.5972
135
134
133
132
131
130
129
128
127
126
f1 (ppm)
180
170
160
150
140
130
120
110
100
90
80
70
60
50
40
30
20
10
0
f1 (ppm)

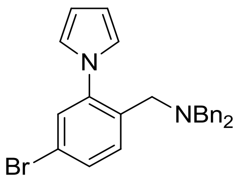
N
NBn2
Br

3h

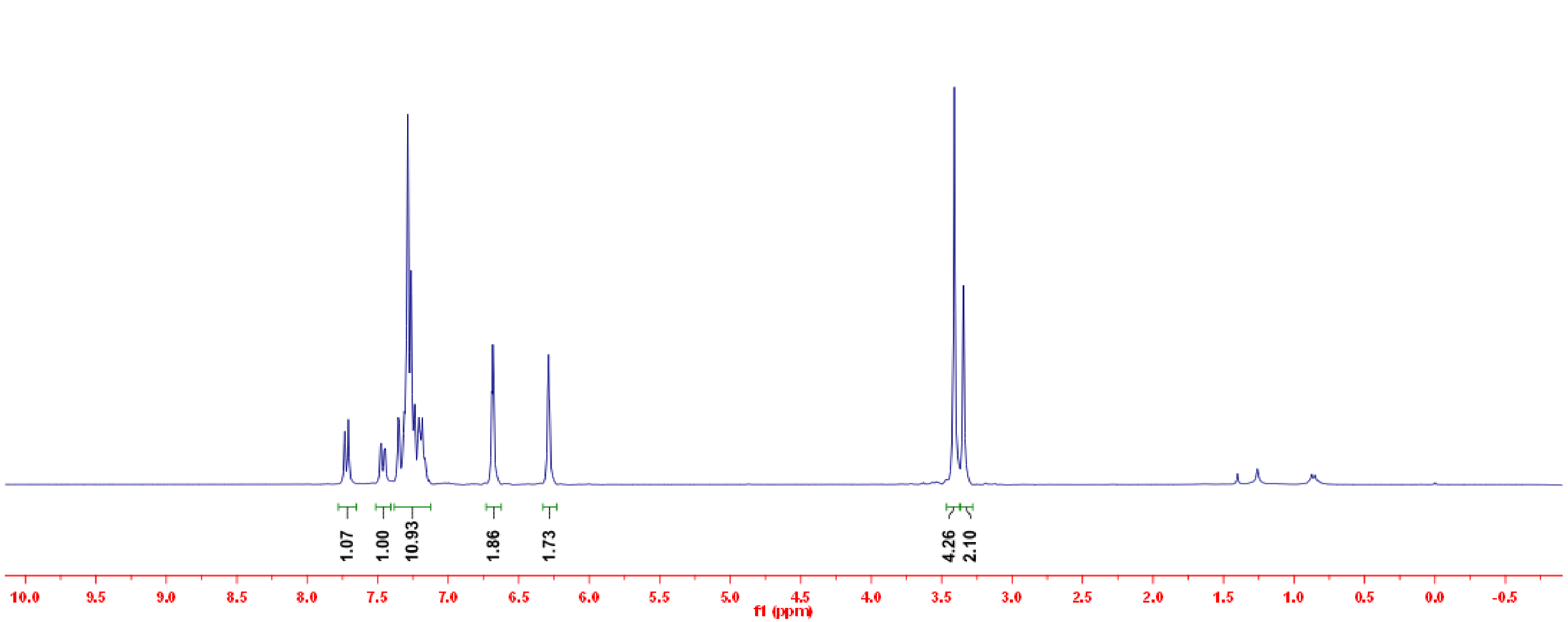
7.7357
7.7078
7.3540
7.2861
7.2076
7.1641
6.6832
6.6764
6.2946
6.2879
6.2813
3.4098
3.3445
-0.0003
1.07
1.00
10.93
1.86
1.73
4.26
2.10
10.0 9.5 9.0 8.5 8.0 7.5 7.0 6.5 6.0 5.5 5.0 4.5 4.0 3.5 3.0 2.5 2.0 1.5 1.0 0.5 0.0 -0.5
f1 (ppm)

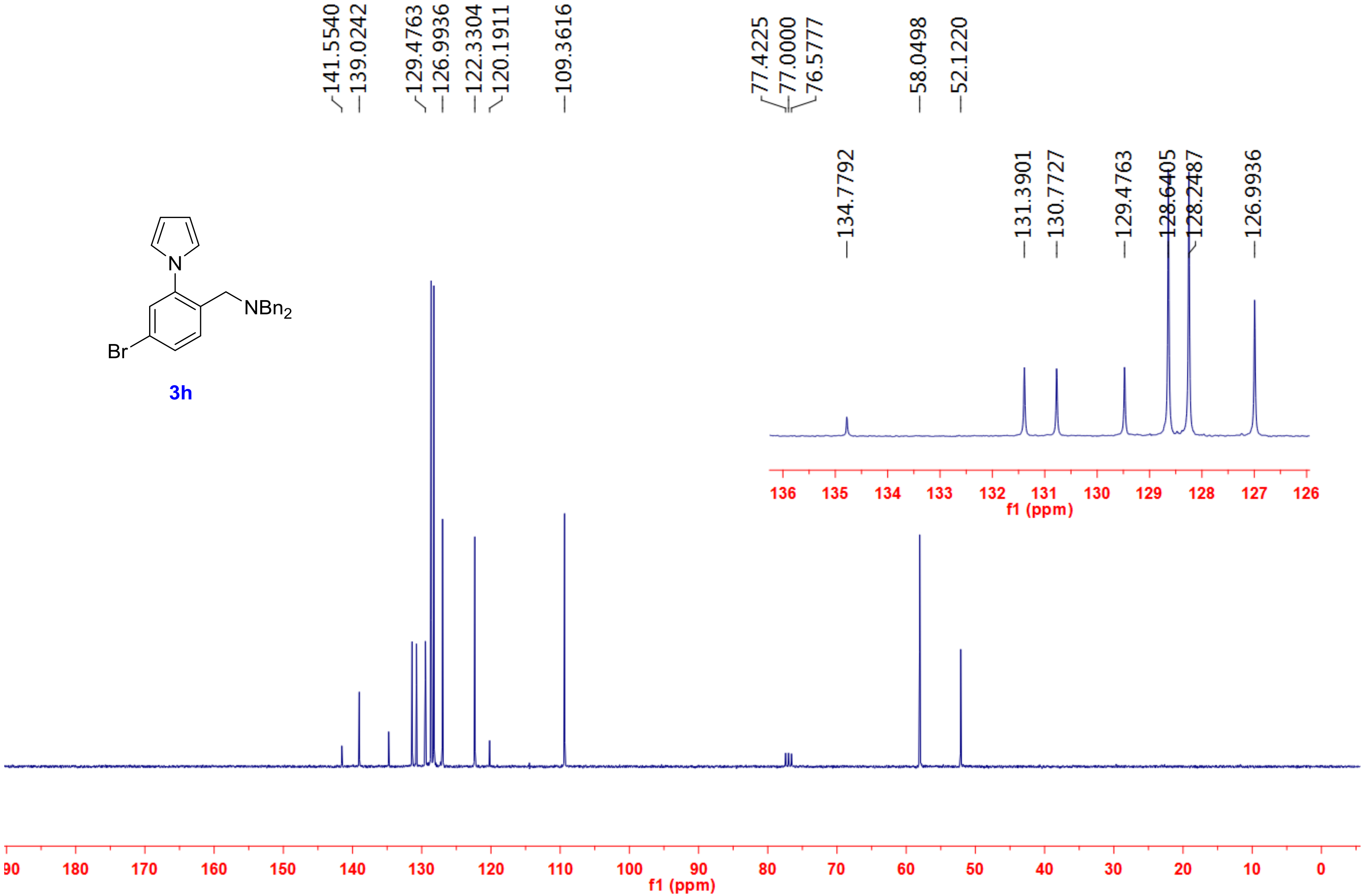
N
NBn2
Br
3h
141.5540
139.0242
129.4763
126.9936
122.3304
120.1911
109.3616
77.4225
77.0000
76.5777
58.0498
52.1220
134.7792
131.3901
130.7727
129.4763
128.6405
128.2487
126.9936
136 135 134 133 132 131 130 129 128 127 126
f1 (ppm)
90 180 170 160 150 140 130 120 110 100 90 80 70 60 50 40 30 20 10 0
f1 (ppm)

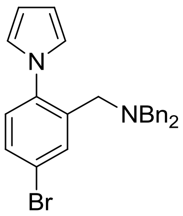

3i

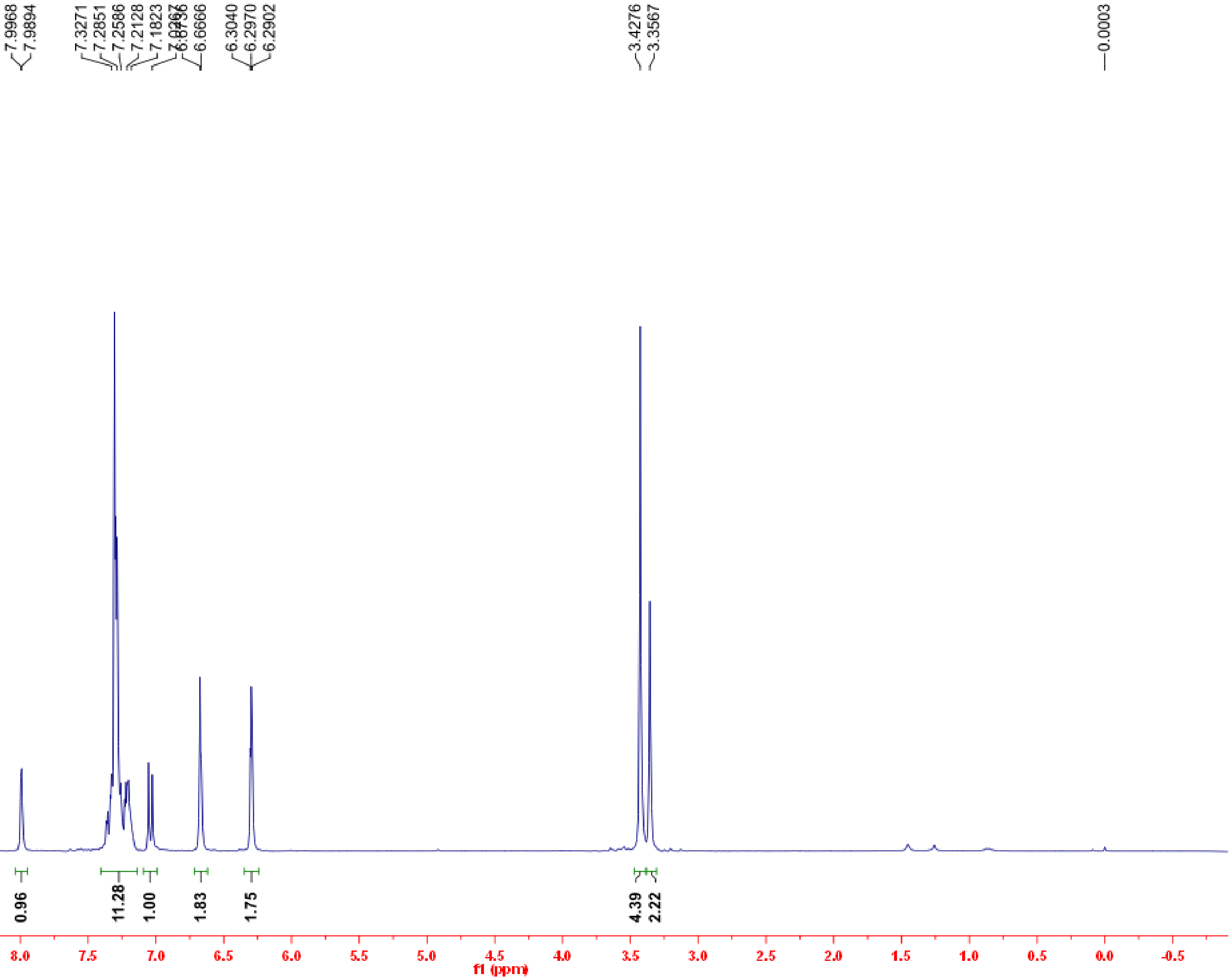
7.9968
7.9894
7.3271
7.2851
7.2586
7.2128
7.1823
6.6666
6.3040
6.2970
6.2902
3.4276
3.3567
0.0003
0.96
11.28
1.00
1.83
1.75
4.39
2.22
9.5 9.0 8.5 8.0 7.5 7.0 6.5 6.0 5.5 5.0 4.5 4.0 3.5 3.0 2.5 2.0 1.5 1.0 0.5 0.0 -0.5
f1 (ppm)

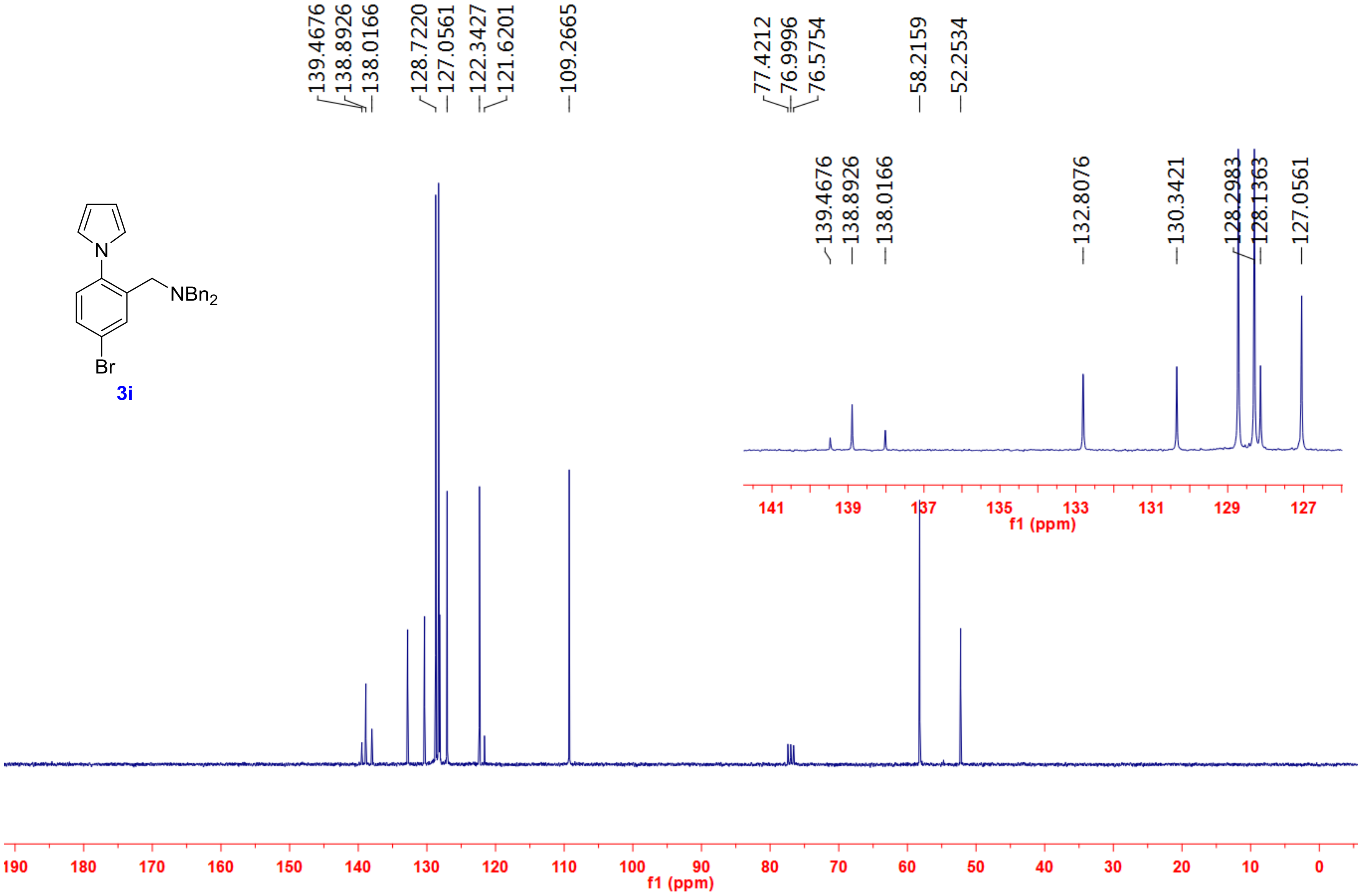
NBn2
Br
139.4676
138.8926
138.0166
128.7220
127.0561
122.3427
121.6201
109.2665
77.4212
76.9996
76.5754
58.2159
52.2534
132.8076
130.3421
128.2983
128.1363
f1 (ppm)

3i

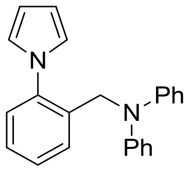

N
Ph
Ph

3j

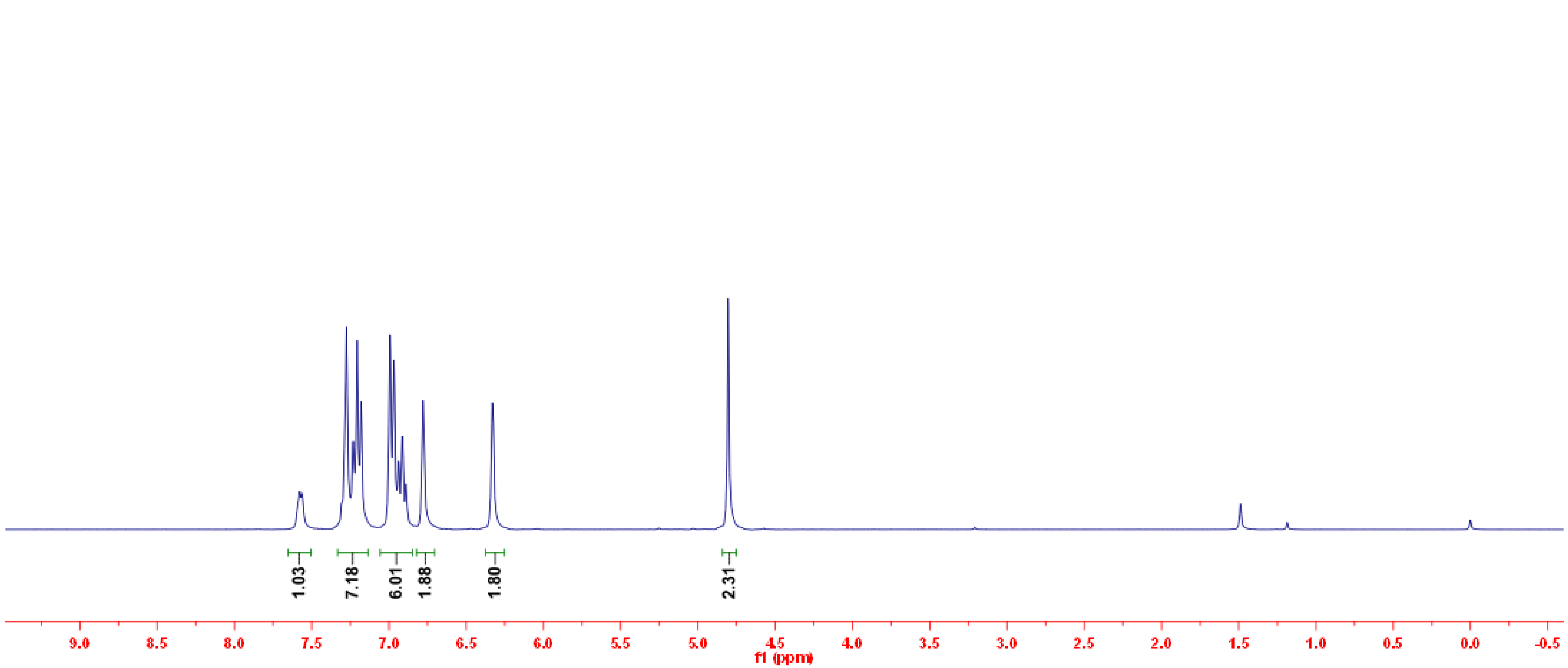

7.5771
7.5627
7.1805
6.9937
6.9134
6.8895
6.7796
6.3305
4.8041
0.0017
-0.0003
1.03
7.18
6.01
1.88
1.80
2.31
9.0
8.5
8.0
7.5
7.0
6.5
6.0
5.5
5.0
4.5
4.0
3.5
3.0
2.5
2.0
1.5
1.0
0.5
0.0
-0.5
f1 (ppm)

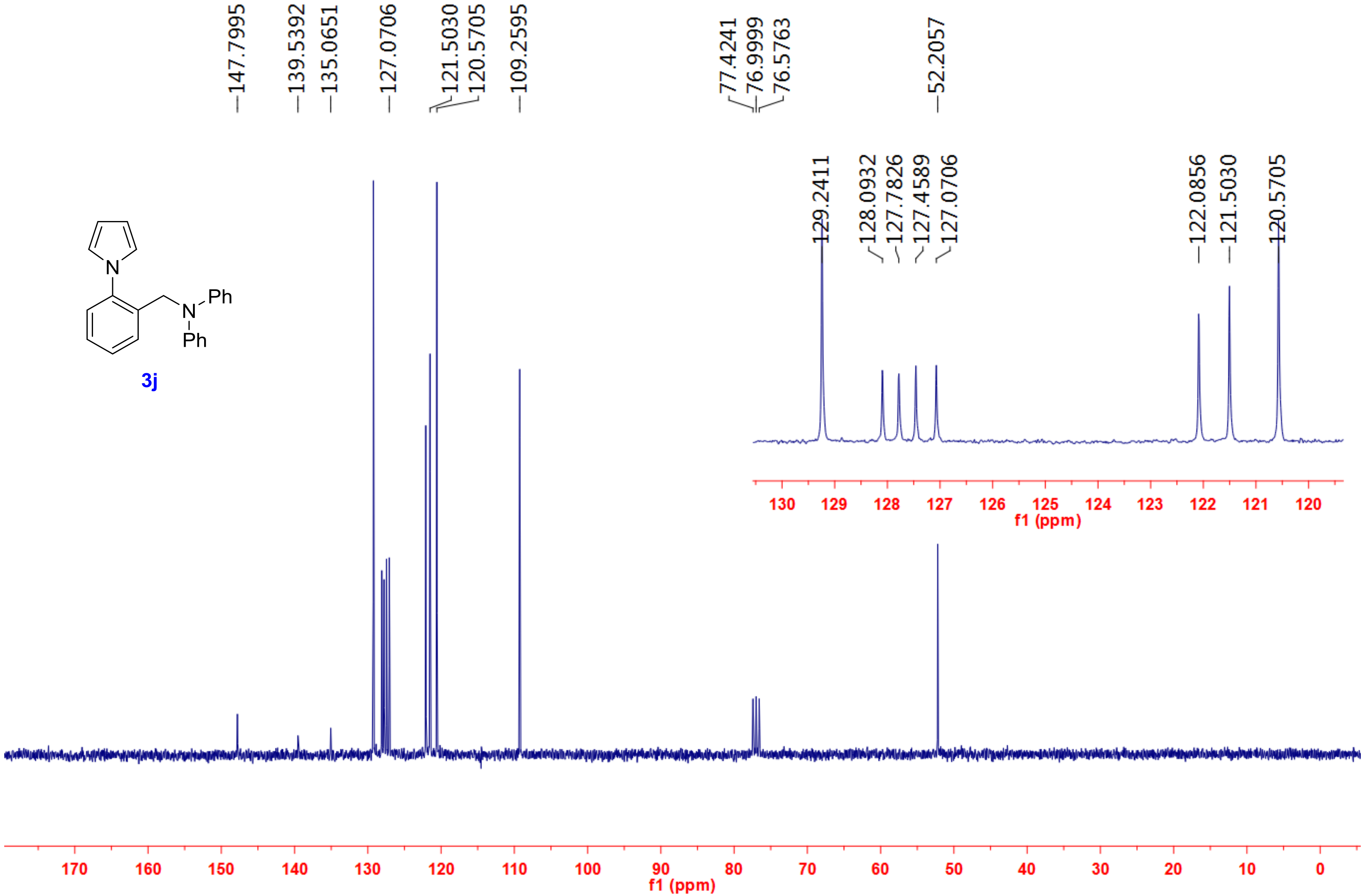
147.7995
139.5392
135.0651
127.0706
121.5030
120.5705
109.2595
77.4241
76.9999
76.5763
52.2057
129.2411
128.0932
127.7826
127.4589
127.0706
122.0856
121.5030
120.5705
N
Ph
Ph
f1 (ppm)
f1 (ppm)
3j

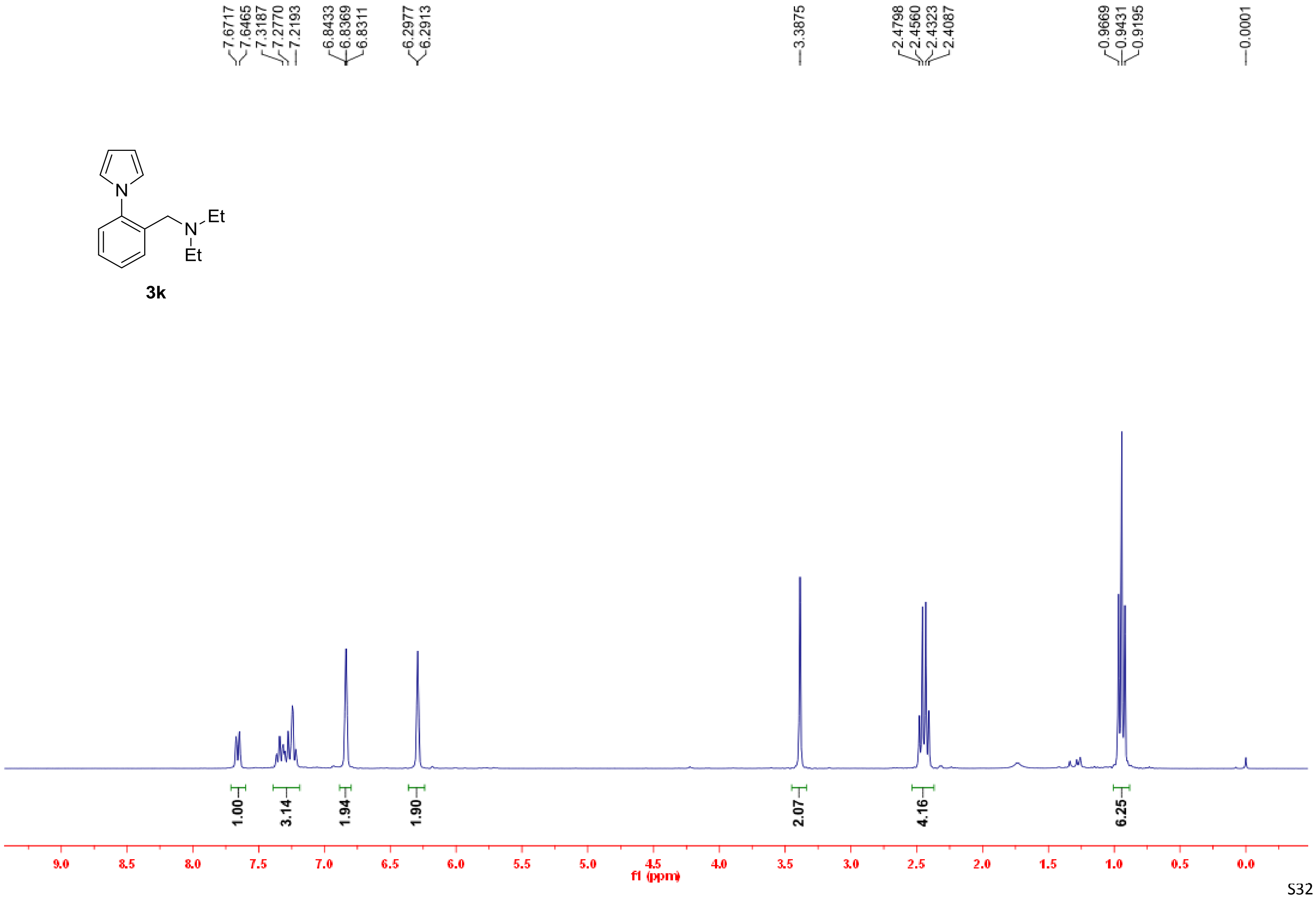

7.6717
7.6465
7.3187
7.2770
7.2193
6.8433
6.8369
6.8311
6.2977
6.2913
3.3875
2.4798
2.4560
2.4323
2.4087
0.9669
0.9431
0.9195
0.0001
N
N
Et
Et
1.00
3.14
1.94
1.90
2.07
4.16
6.25
9.0
8.5
8.0
7.5
7.0
6.5
6.0
5.5
5.0
4.5
4.0
3.5
3.0
2.5
2.0
1.5
1.0
0.5
0.0
f1 (ppm)

3k

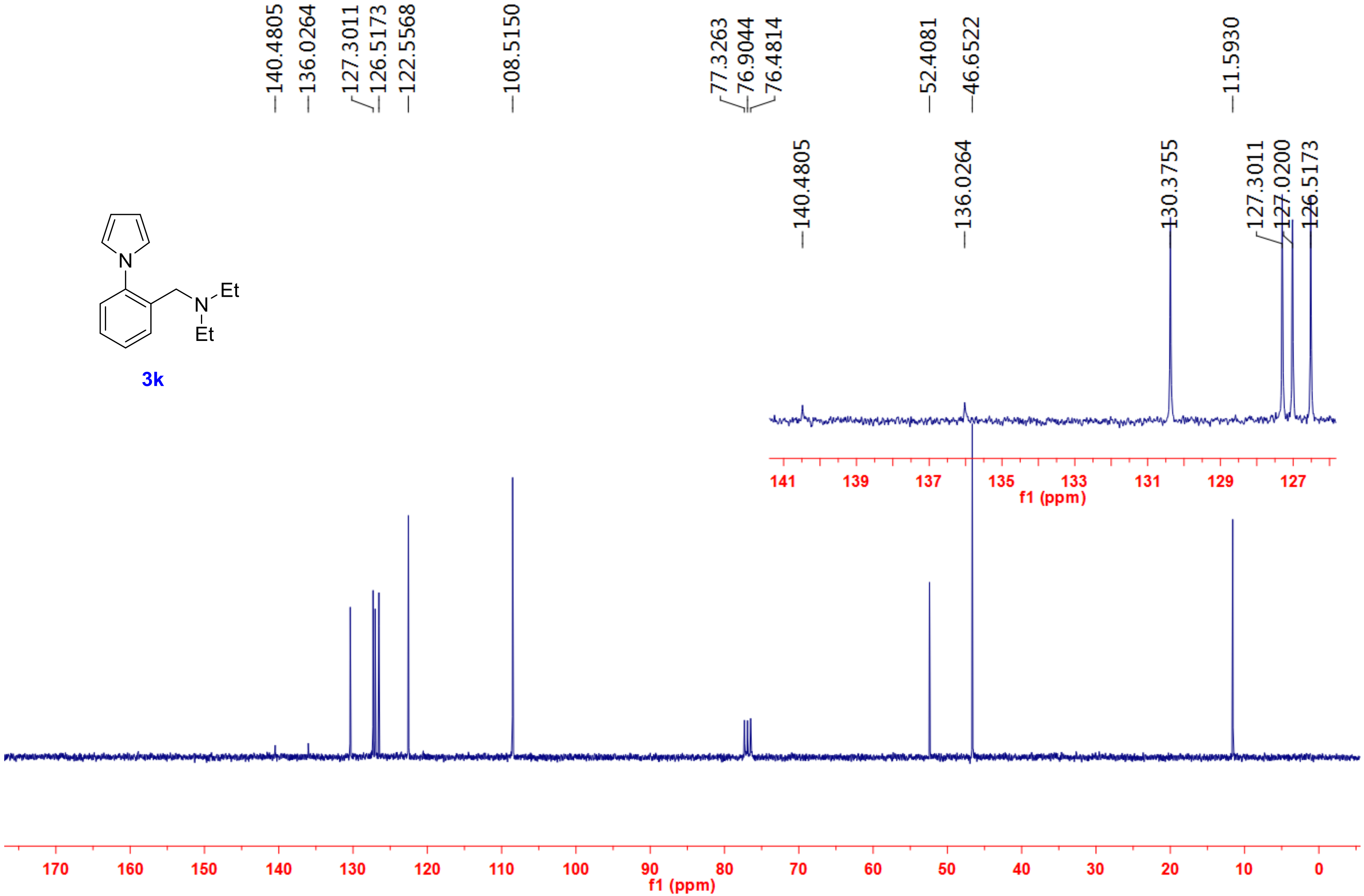
N
N
Et
Et
3k
140.4805
136.0264
127.3011
126.5173
122.5568
108.5150
77.3263
76.9044
76.4814
52.4081
46.6522
11.5930
140.4805
136.0264
130.3755
127.3011
127.0200
126.5173
141 139 137 135 133 131 129 127
f1 (ppm)
170 160 150 140 130 120 110 100 90 80 70 60 50 40 30 20 10 0
f1 (ppm)

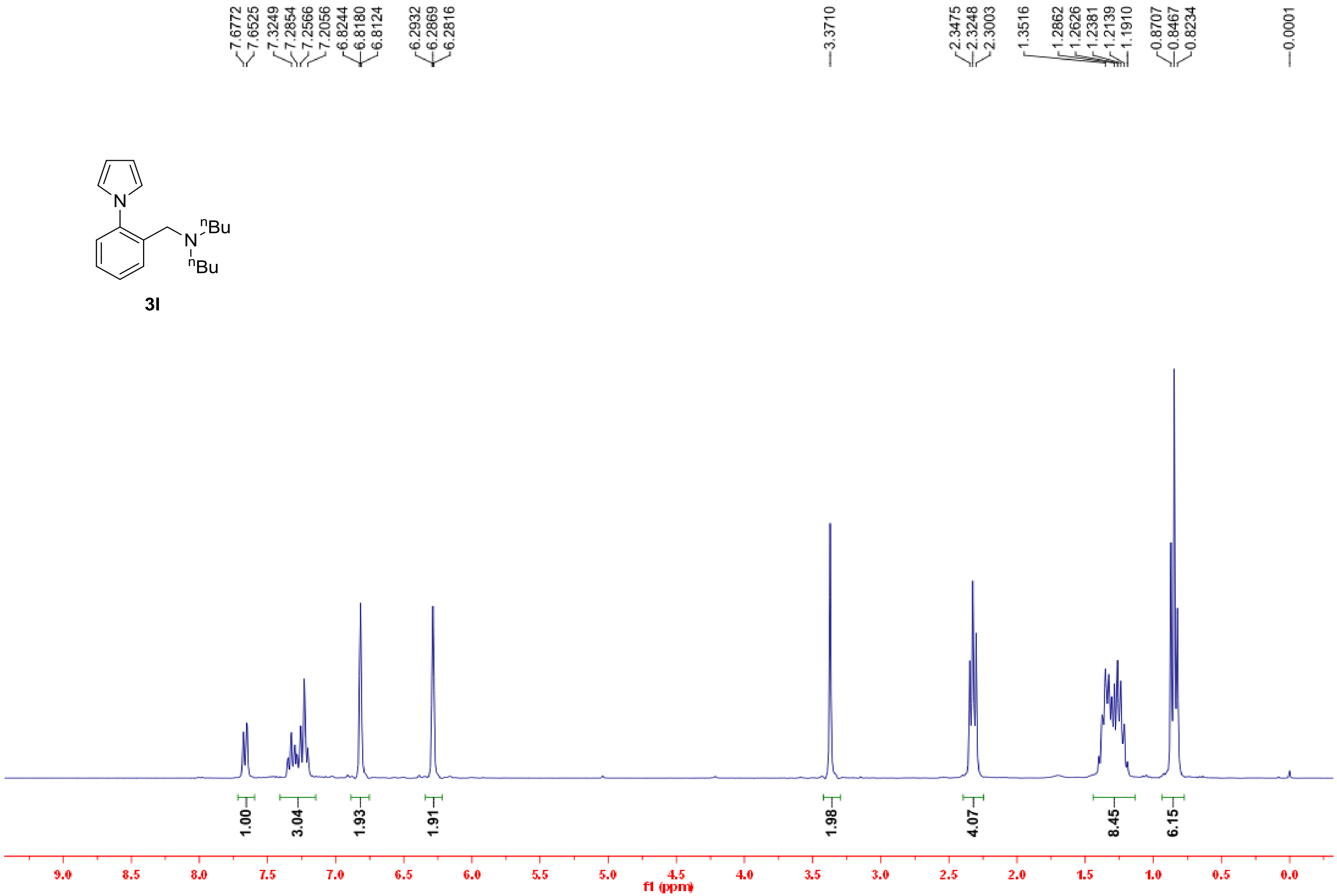

N
nBu
nBu
3l
7.6772
7.6525
7.3249
7.2854
7.2566
7.2056
6.8244
6.8180
6.8124
6.2932
6.2869
6.2816
3.3710
2.3475
2.3248
2.3003
1.3516
1.2862
1.2626
1.2381
1.2139
1.1910
0.8707
0.8467
0.8234
0.0001
1.00
3.04
1.93
1.91
1.98
4.07
8.45
6.15
9.0
8.5
8.0
7.5
7.0
6.5
6.0
5.5
5.0
4.5
4.0
3.5
3.0
2.5
2.0
1.5
1.0
0.5
0.0
f1 (ppm)

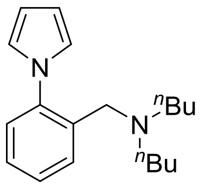

3l

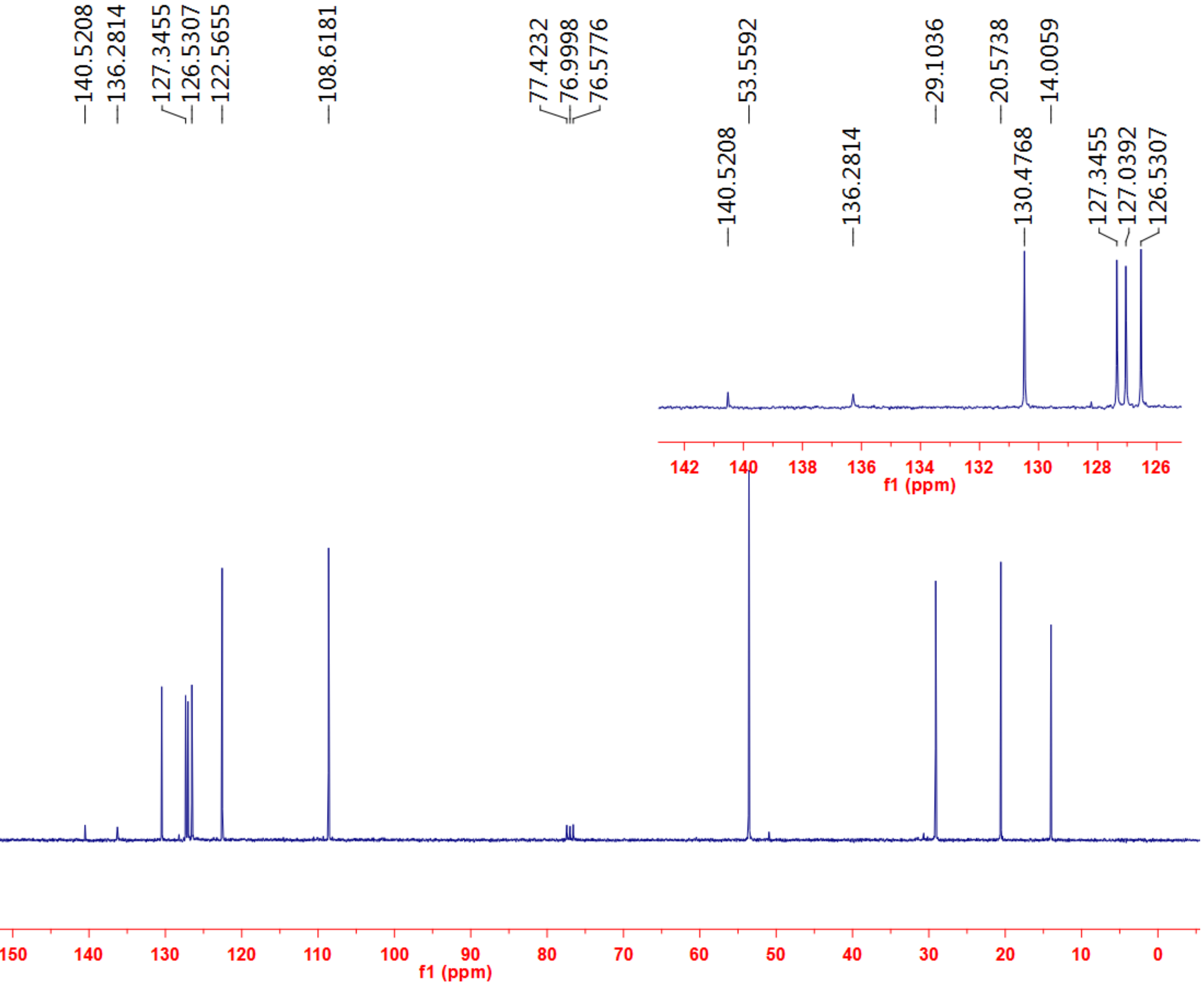

140.5208
136.2814
127.3455
126.5307
122.5655
108.6181
77.4232
76.9998
76.5776
53.5592
29.1036
20.5738
14.0059
140.5208
136.2814
130.4768
127.3455
127.0392
126.5307
142
140
138
136
134
132
130
128
126
f1 (ppm)
180
170
160
150
140
130
120
110
100
90
80
70
60
50
40
30
20
10
0
f1 (ppm)

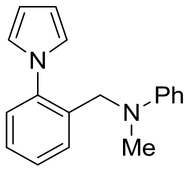

3m

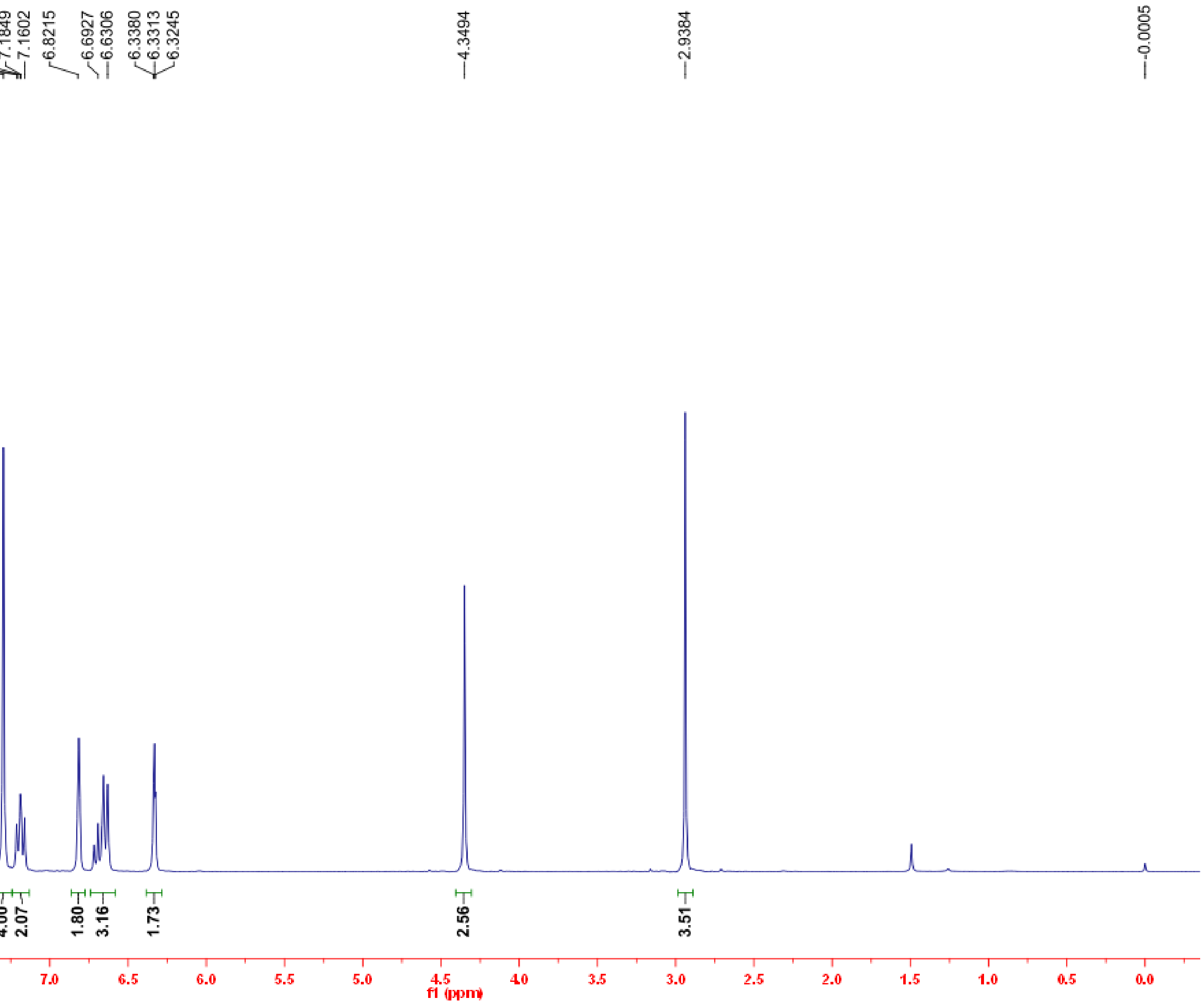
7.2966
7.2135
7.2063
7.1889
7.1849
7.1602
6.8215
6.6927
6.6306
6.3380
6.3313
6.3245
4.3494
2.9384
-0.0005
4.00
2.07
1.80
3.16
1.73
2.56
3.51
9.0
8.5
8.0
7.5
7.0
6.5
6.0
5.5
5.0
4.5
4.0
3.5
3.0
2.5
2.0
1.5
1.0
0.5
0.0
f1 (ppm)

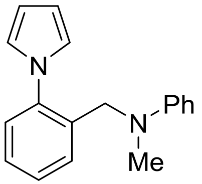
N
N
Ph
Me

3m

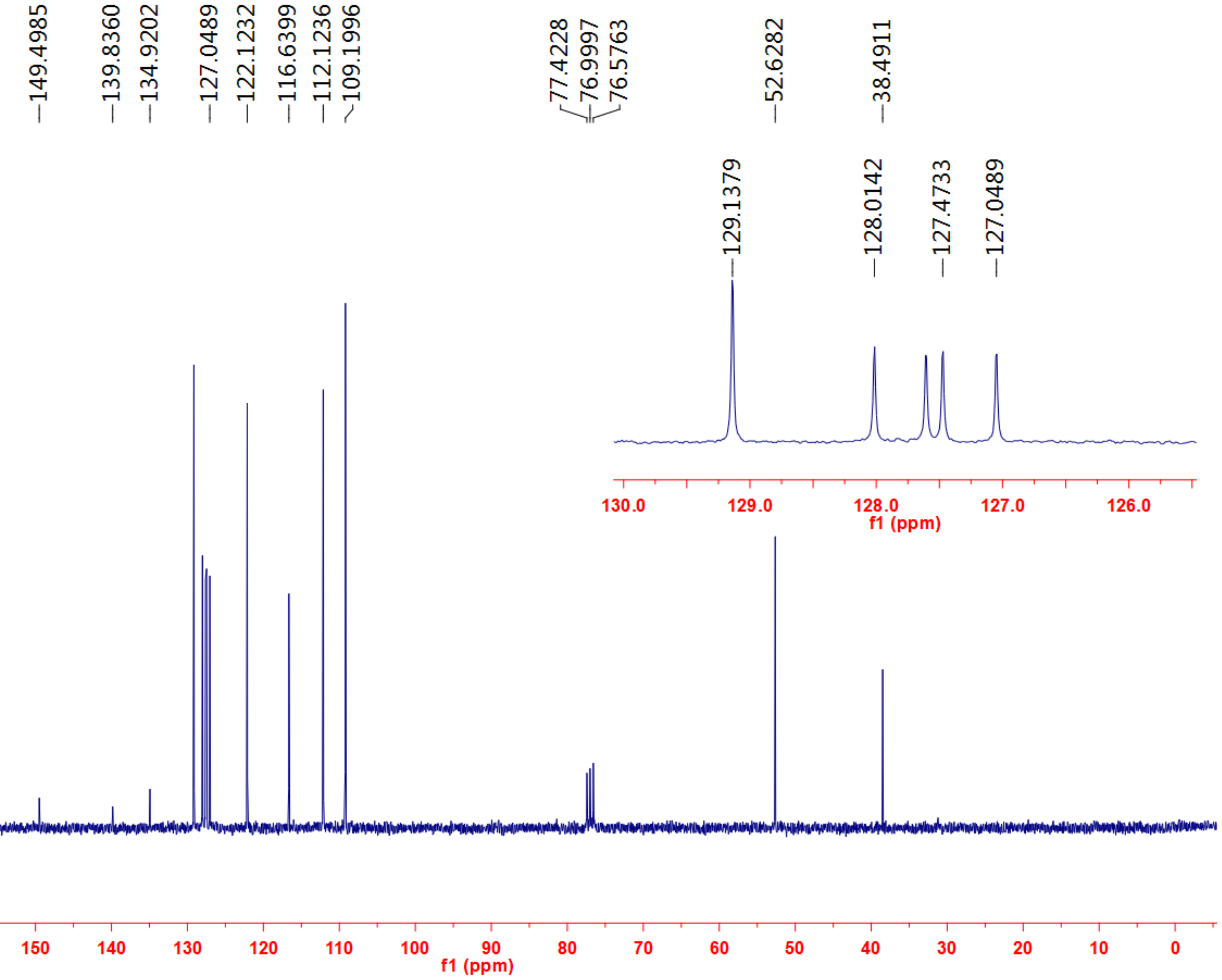
149.4985
139.8360
134.9202
127.0489
122.1232
116.6399
112.1236
109.1996
77.4228
76.9997
76.5763
52.6282
38.4911
129.1379
128.0142
127.4733
127.0489
130.0
129.0
128.0
127.0
126.0
f1 (ppm)
180
170
160
150
140
130
120
110
100
90
80
70
60
50
40
30
20
10
0
f1 (ppm)

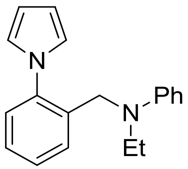
N
Ph
Et

3n

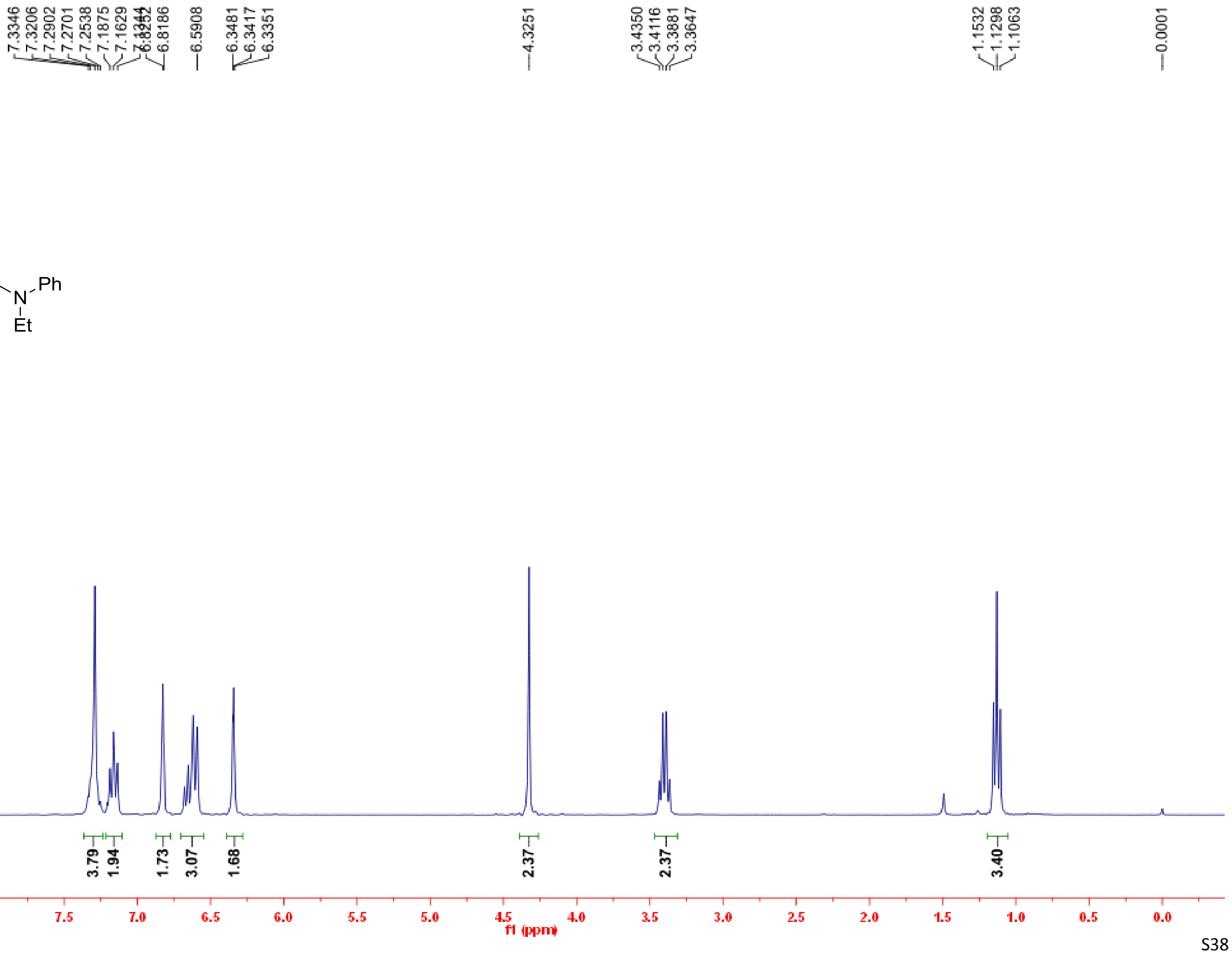
7.3346
7.3206
7.2902
7.2701
7.2538
7.1875
7.1629
6.8186
6.5908
6.3481
6.3417
6.3351
4.3251
3.4350
3.4116
3.3881
3.3647
1.1532
1.1298
1.1063
0.0001
3.79
1.94
1.73
3.07
1.68
2.37
2.37
3.40
8.5
8.0
7.5
7.0
6.5
6.0
5.5
5.0
4.5
4.0
3.5
3.0
2.5
2.0
1.5
1.0
0.5
0.0
f1 (ppm)

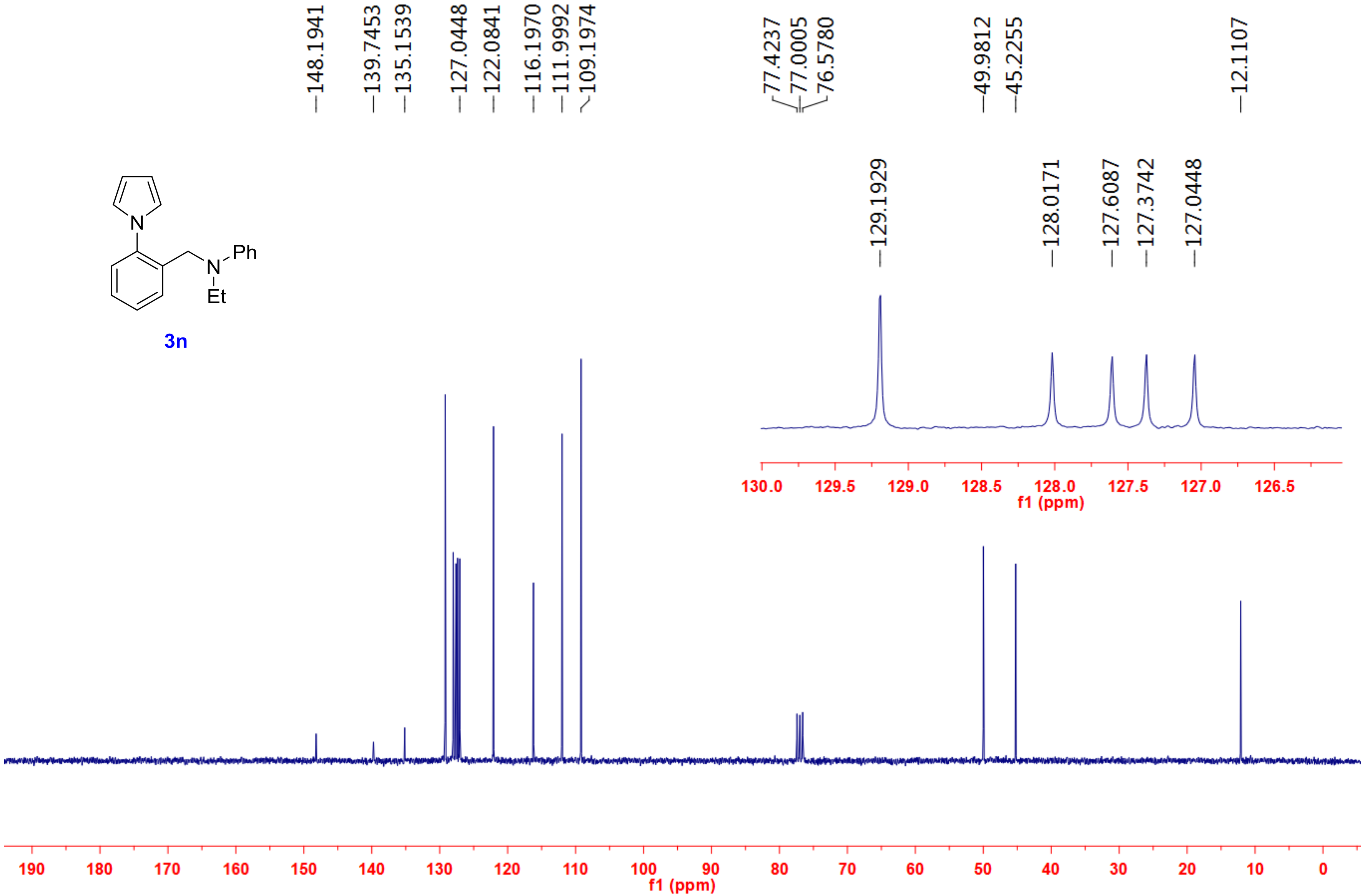
N
Ph
N
Et
3n
148.1941
139.7453
135.1539
127.0448
122.0841
116.1970
111.9992
109.1974
77.4237
77.0005
76.5780
49.9812
45.2255
12.1107
129.1929
128.0171
127.6087
127.3742
127.0448
130.0 129.5 129.0 128.5 128.0 127.5 127.0 126.5
f1 (ppm)
190 180 170 160 150 140 130 120 110 100 90 80 70 60 50 40 30 20 10 0
f1 (ppm)

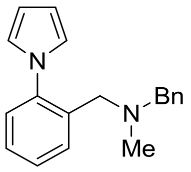
N
N
Bn
Me

3o

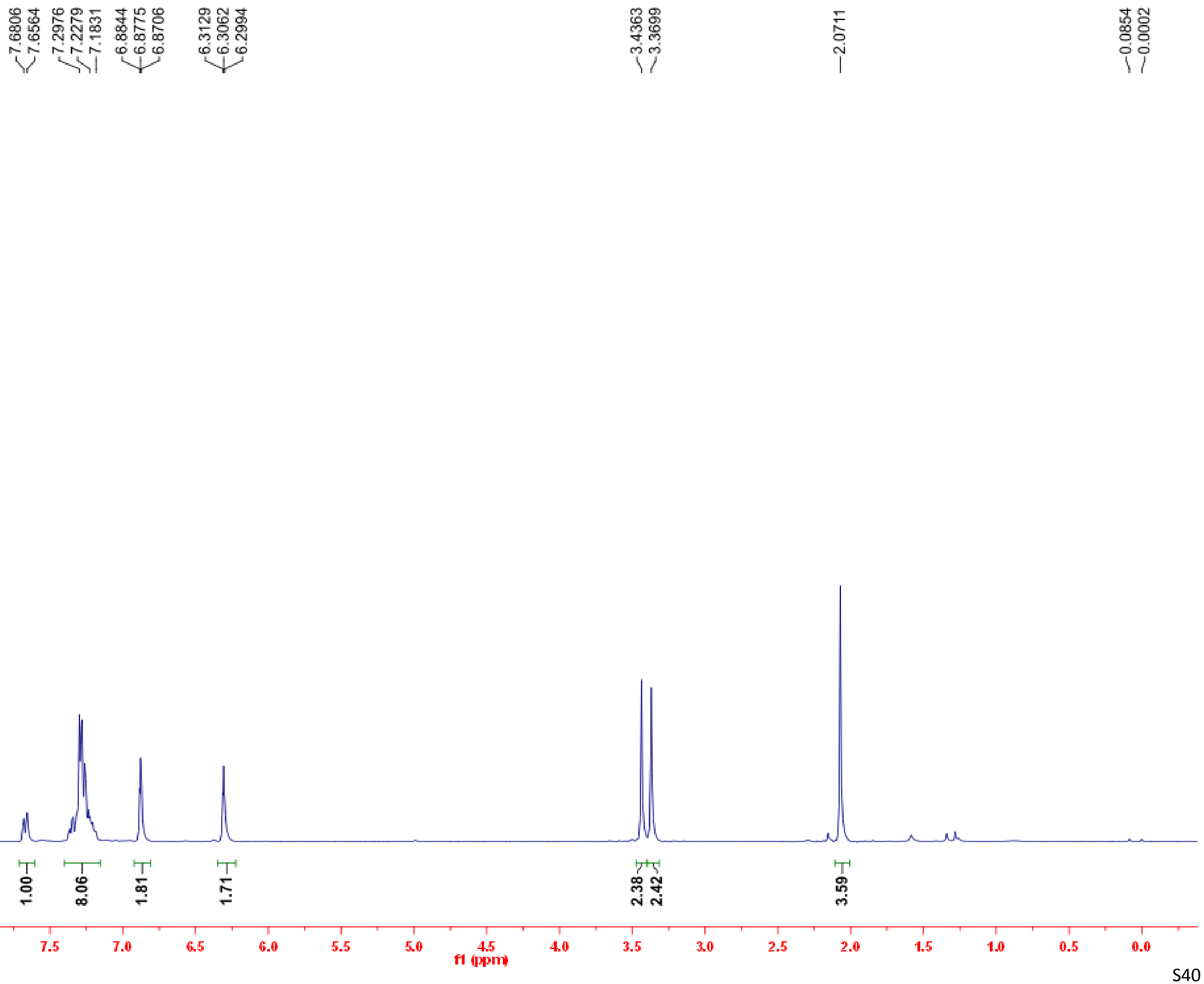
7.6806
7.6564
7.2976
7.2279
7.1831
6.8844
6.8775
6.8706
6.3129
6.3062
6.2994
3.4363
3.3699
2.0711
0.0854
0.0002
1.00
8.06
1.81
1.71
2.38
2.42
3.59
9.0
8.5
8.0
7.5
7.0
6.5
6.0
5.5
5.0
4.5
4.0
3.5
3.0
2.5
2.0
1.5
1.0
0.5
0.0
f1 (ppm)

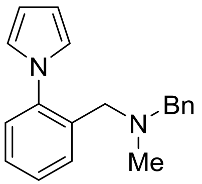
N
Bn
N
Me

3o

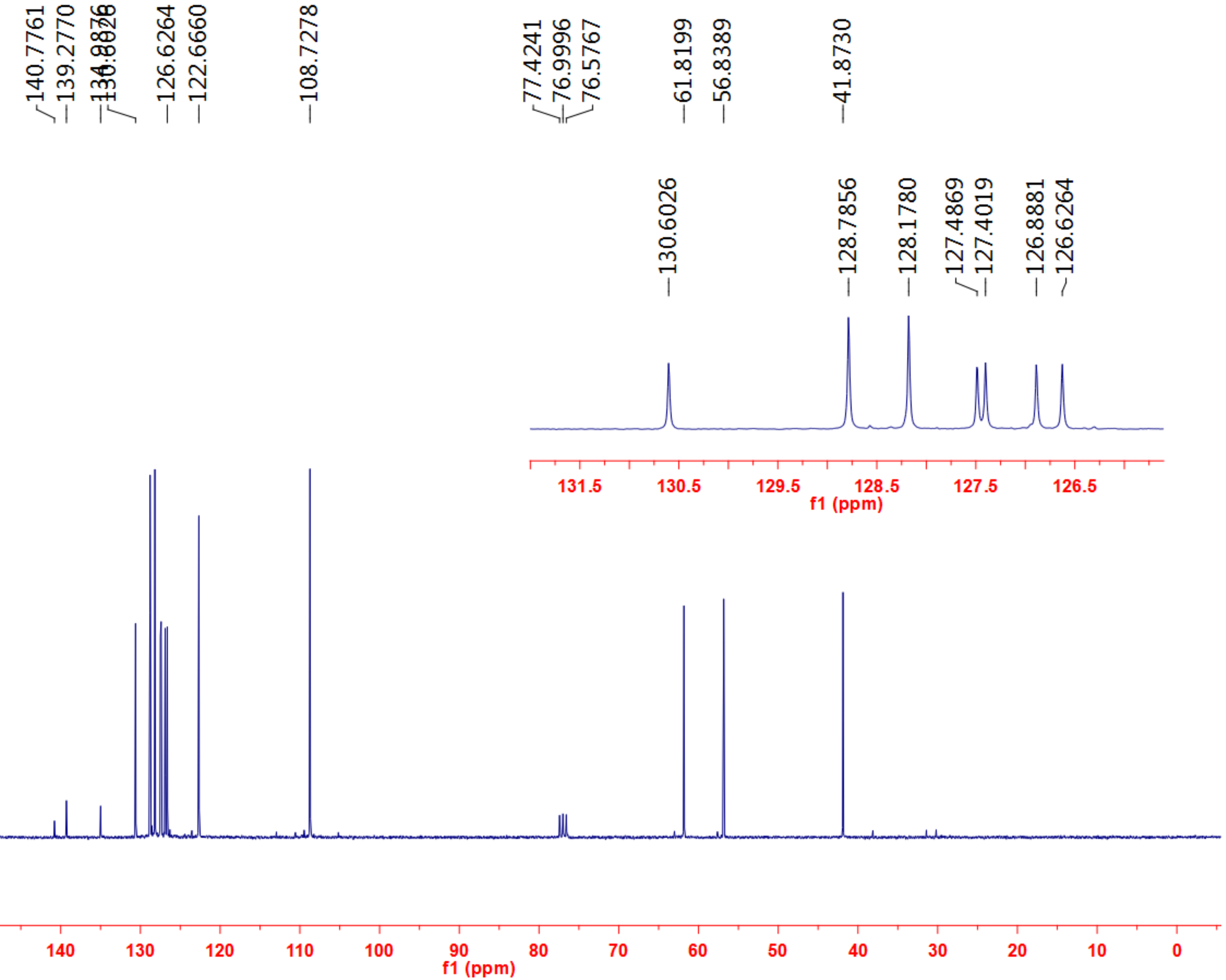
140.7761
139.2770
126.6264
122.6660
108.7278
77.4241
76.9996
76.5767
61.8199
56.8389
41.8730
130.6026
128.7856
128.1780
127.4869
127.4019
126.8881
126.6264
131.5
130.5
129.5
128.5
127.5
126.5
f1 (ppm)
80
170
160
150
140
130
120
110
100
90
80
70
60
50
40
30
20
10
0
f1 (ppm)

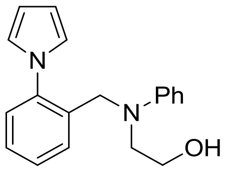
N
Ph
N
OH

3p

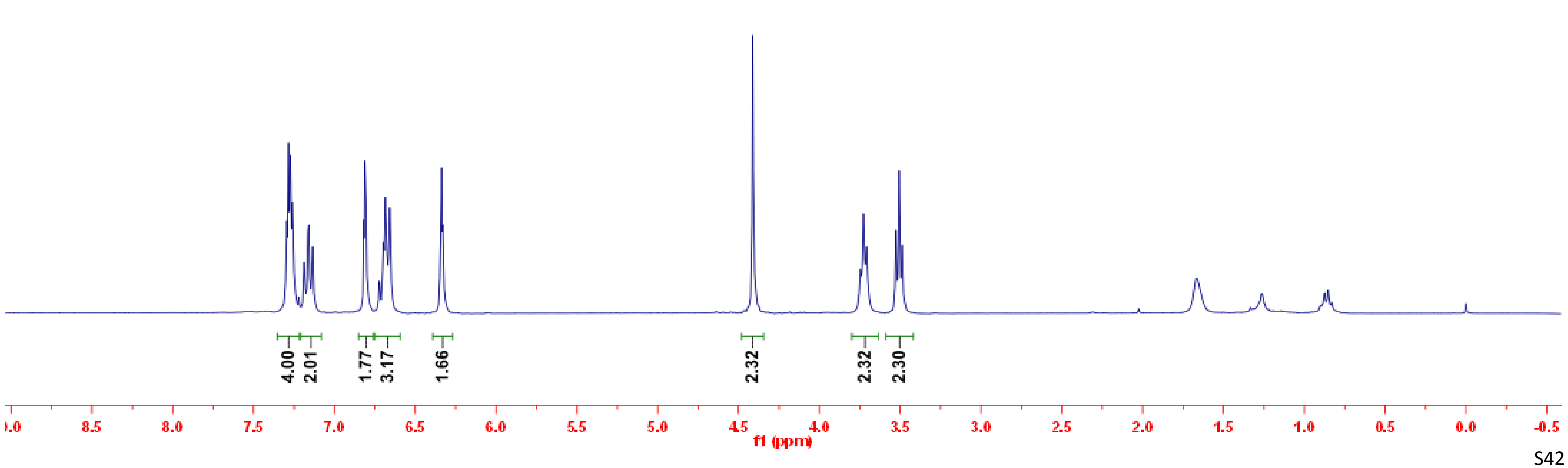
7.2945
7.2849
7.2723
7.2604
7.1877
7.1630
7.1587
7.1343
6.8046
6.6962
6.6586
6.3442
6.3372
6.3304
4.4124
3.7458
3.7267
3.7078
3.5262
3.5069
3.4873
-0.0001
4.00
2.01
1.77
3.17
1.66
2.32
2.32
2.30
8.5
8.0
7.5
7.0
6.5
6.0
5.5
5.0
4.5
4.0
3.5
3.0
2.5
2.0
1.5
1.0
0.5
0.0
-0.5
f1 (ppm)

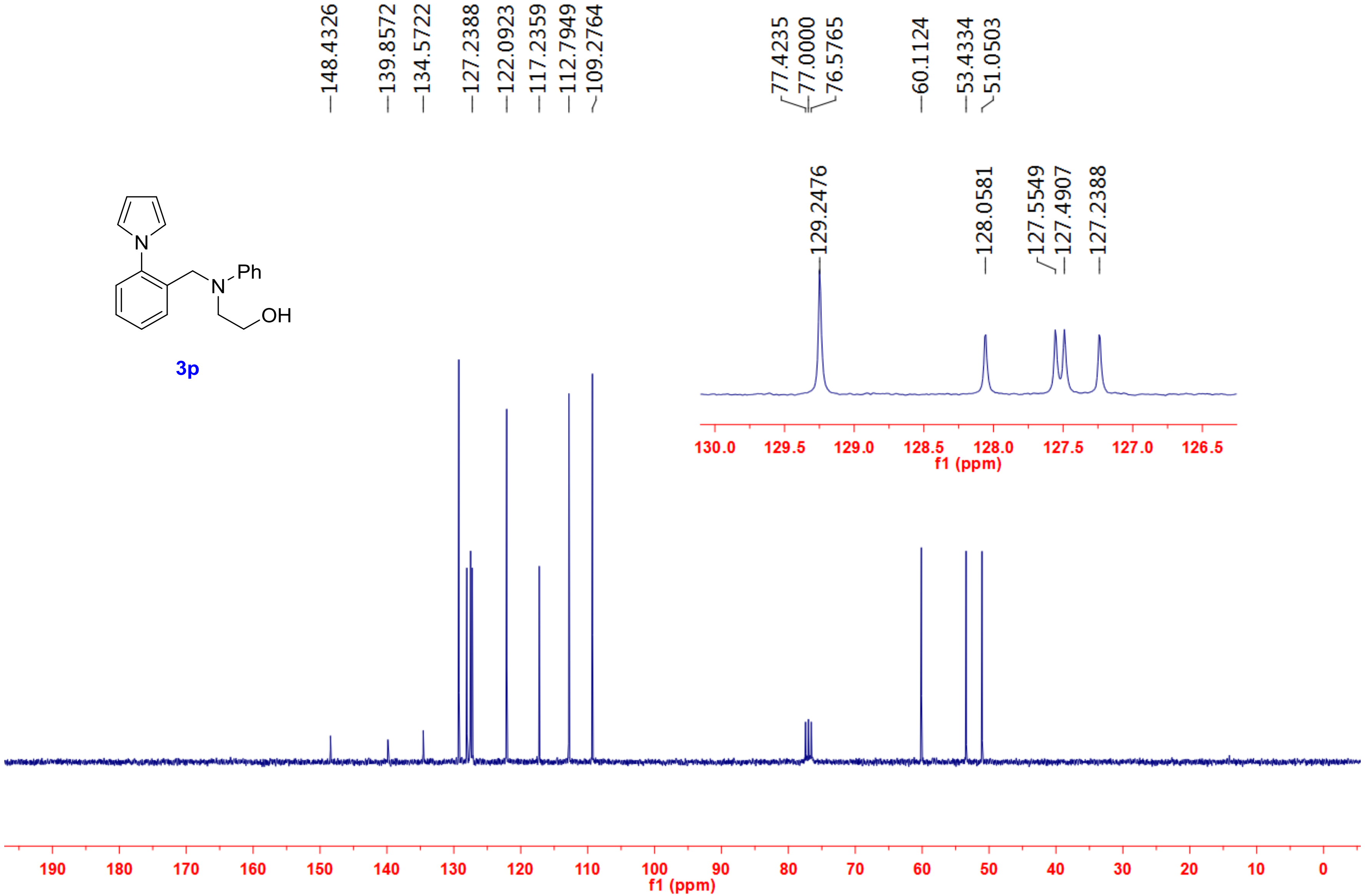
N
N
Ph
OH
3p
148.4326
139.8572
134.5722
127.2388
122.0923
117.2359
112.7949
109.2764
77.4235
77.0000
76.5765
60.1124
53.4334
51.0503
129.2476
128.0581
127.5549
127.4907
127.2388
130.0 129.5 129.0 128.5 128.0 127.5 127.0 126.5
f1 (ppm)
190 180 170 160 150 140 130 120 110 100 90 80 70 60 50 40 30 20 10 0
f1 (ppm)

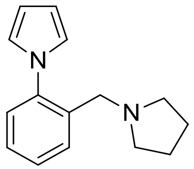

3q

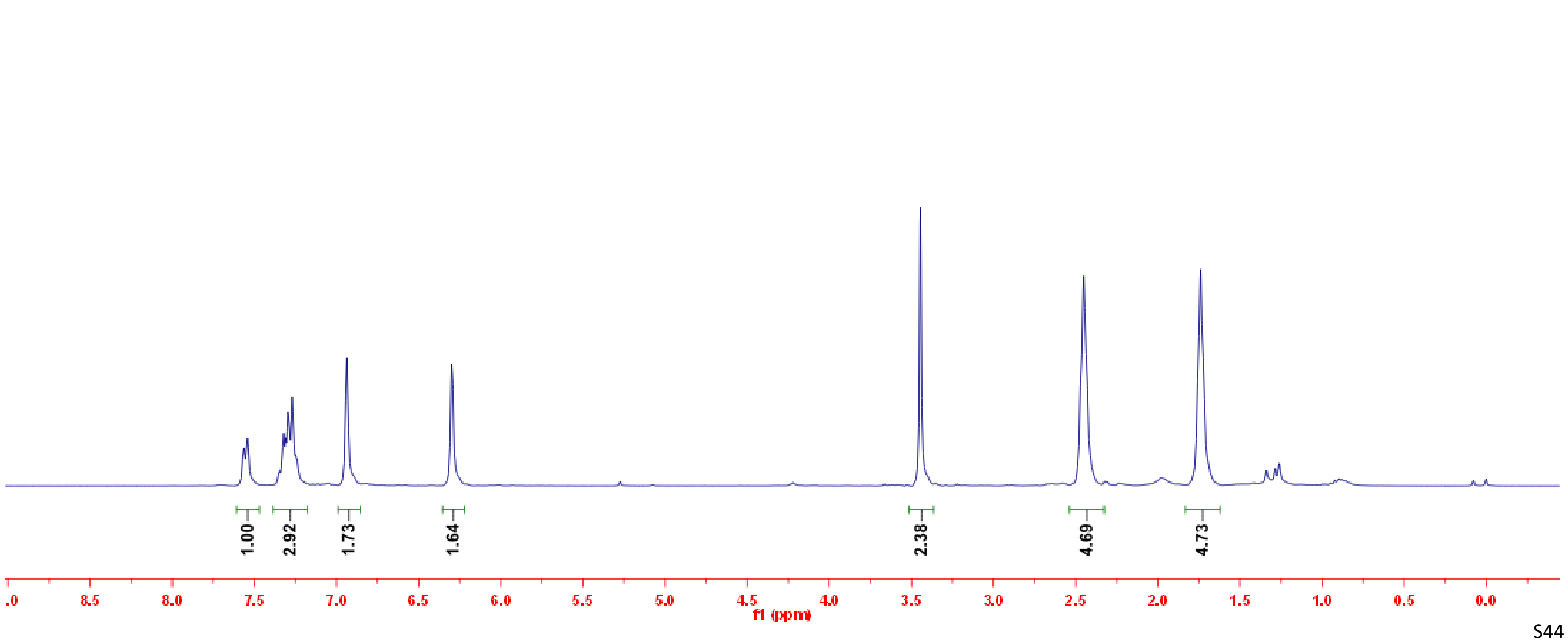

7.5628
7.5406
7.2385
6.9394
6.9366
6.2989
6.2960
3.4454
2.4513
1.7386
0.0772
0.0031
0.0004
1.00
2.92
1.73
1.64
2.38
4.69
4.73
8.5
8.0
7.5
7.0
6.5
6.0
5.5
5.0
4.5
4.0
3.5
3.0
2.5
2.0
1.5
1.0
0.5
0.0
f1 (ppm)

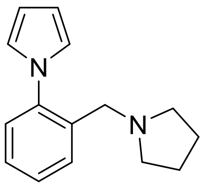

3q

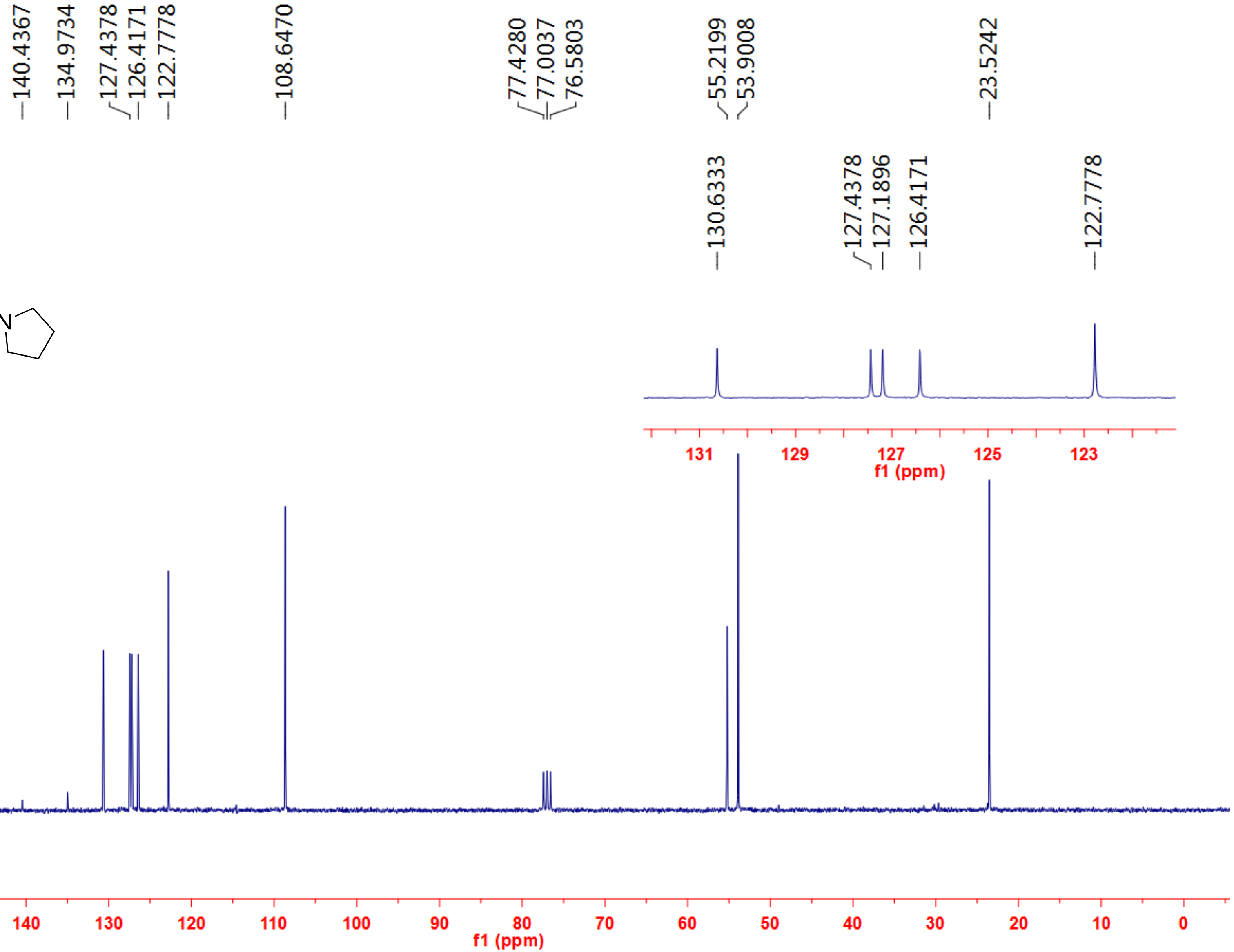
140.4367
134.9734
127.4378
126.4171
122.7778
108.6470
77.4280
77.0037
76.5803
55.2199
53.9008
23.5242
130.6333
127.4378
127.1896
126.4171
122.7778
131
129
127
125
123
f1 (ppm)
160
150
140
130
120
110
100
90
80
70
60
50
40
30
20
10
0
f1 (ppm)

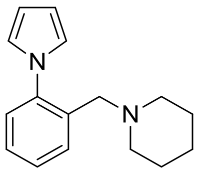

3r

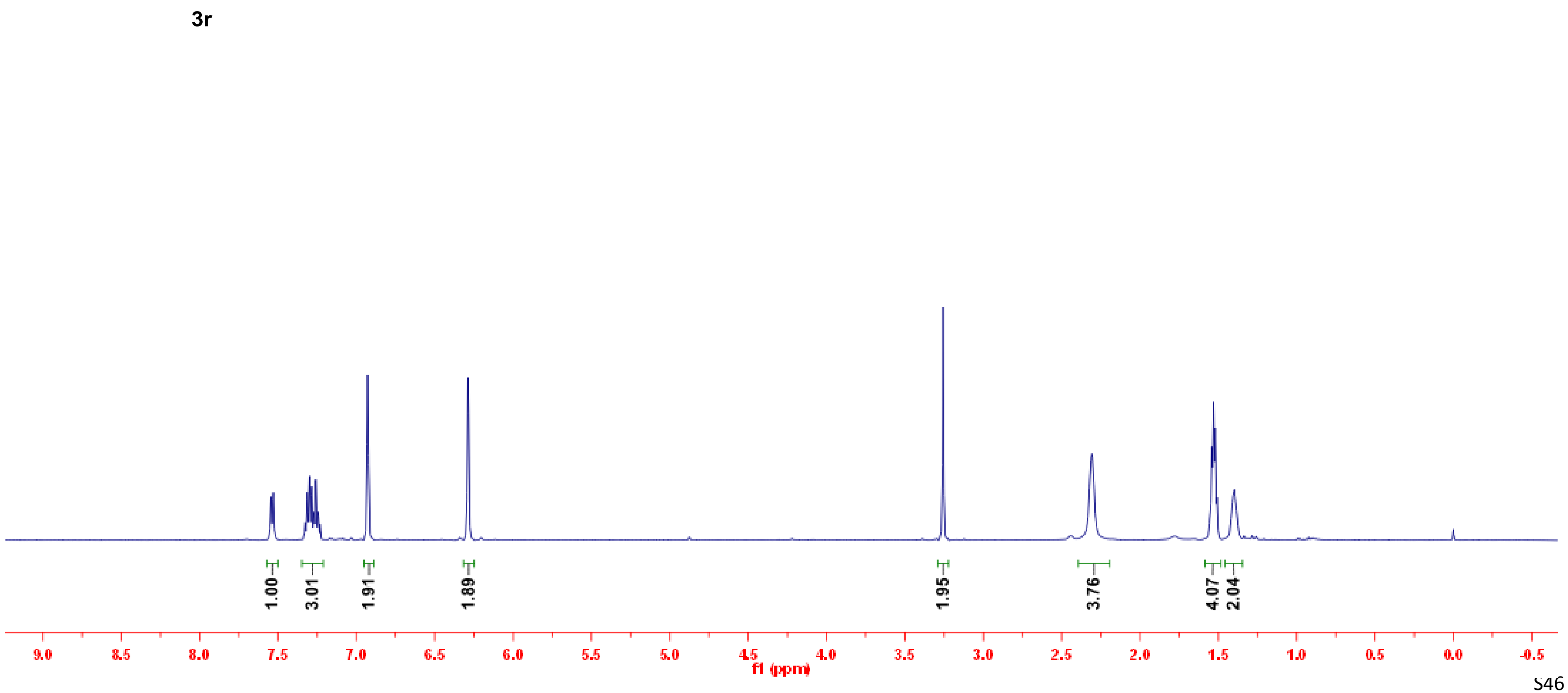
7.5445
7.5302
7.2325
6.9326
6.9286
6.9245
6.2915
6.2875
6.2836
3.2568
2.3071
1.5528
1.5413
1.5301
1.5192
1.5085
1.4053
1.3965
0.0004
1.00
3.01
1.91
1.89
1.95
3.76
4.07
2.04
9.0
8.5
8.0
7.5
7.0
6.5
6.0
5.5
5.0
4.5
4.0
3.5
3.0
2.5
2.0
1.5
1.0
0.5
0.0
-0.5
f1 (ppm)

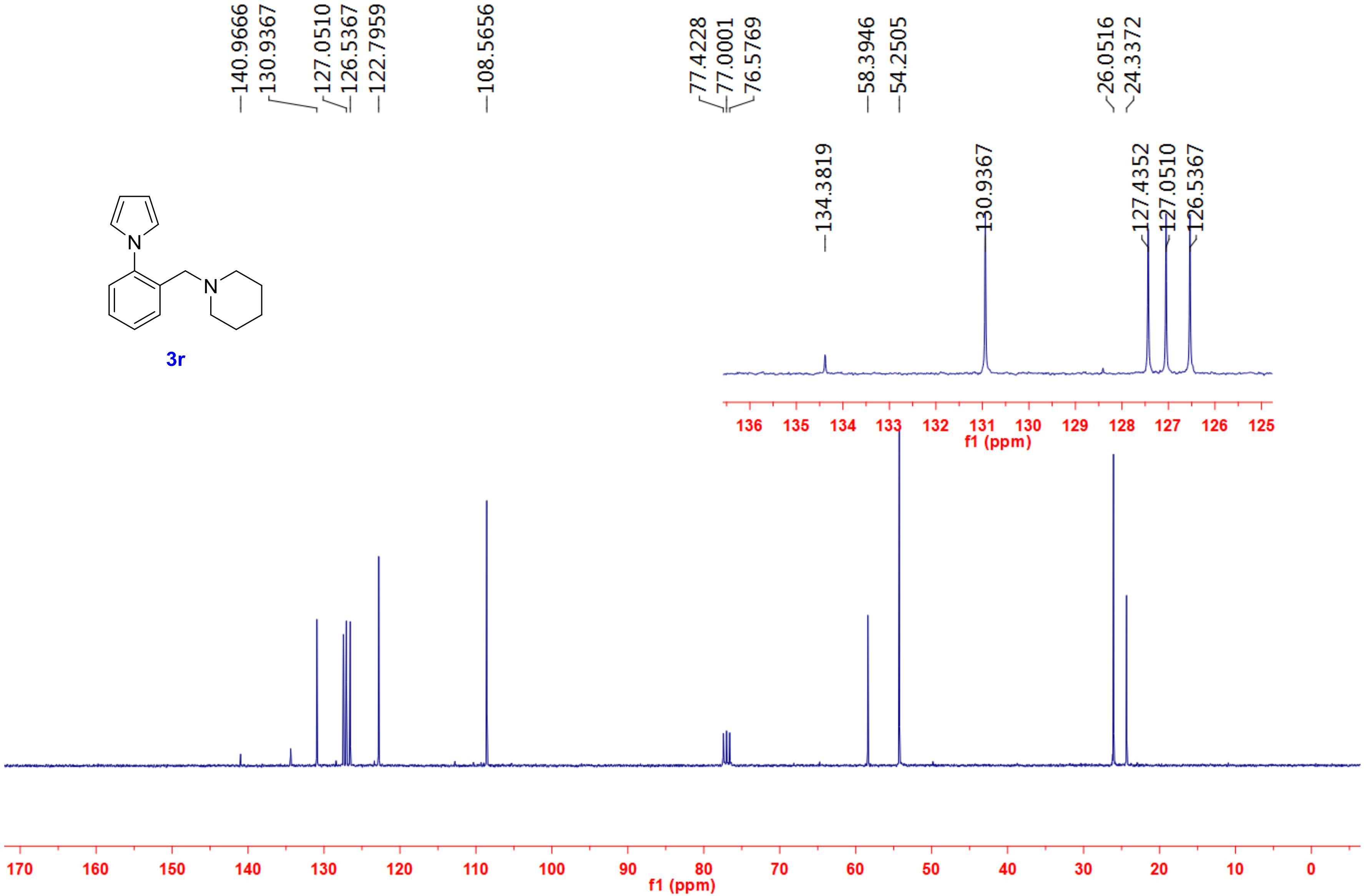
140.9666
130.9367
127.0510
126.5367
122.7959
108.5656
77.4228
77.0001
76.5769
58.3946
54.2505
26.0516
24.3372
N
N
134.3819
130.9367
127.4352
127.0510
126.5367
136 135 134 133 132 131 130 129 128 127 126 125
f1 (ppm)
170 160 150 140 130 120 110 100 90 80 70 60 50 40 30 20 10 0
f1 (ppm)

3r

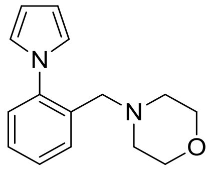

3s

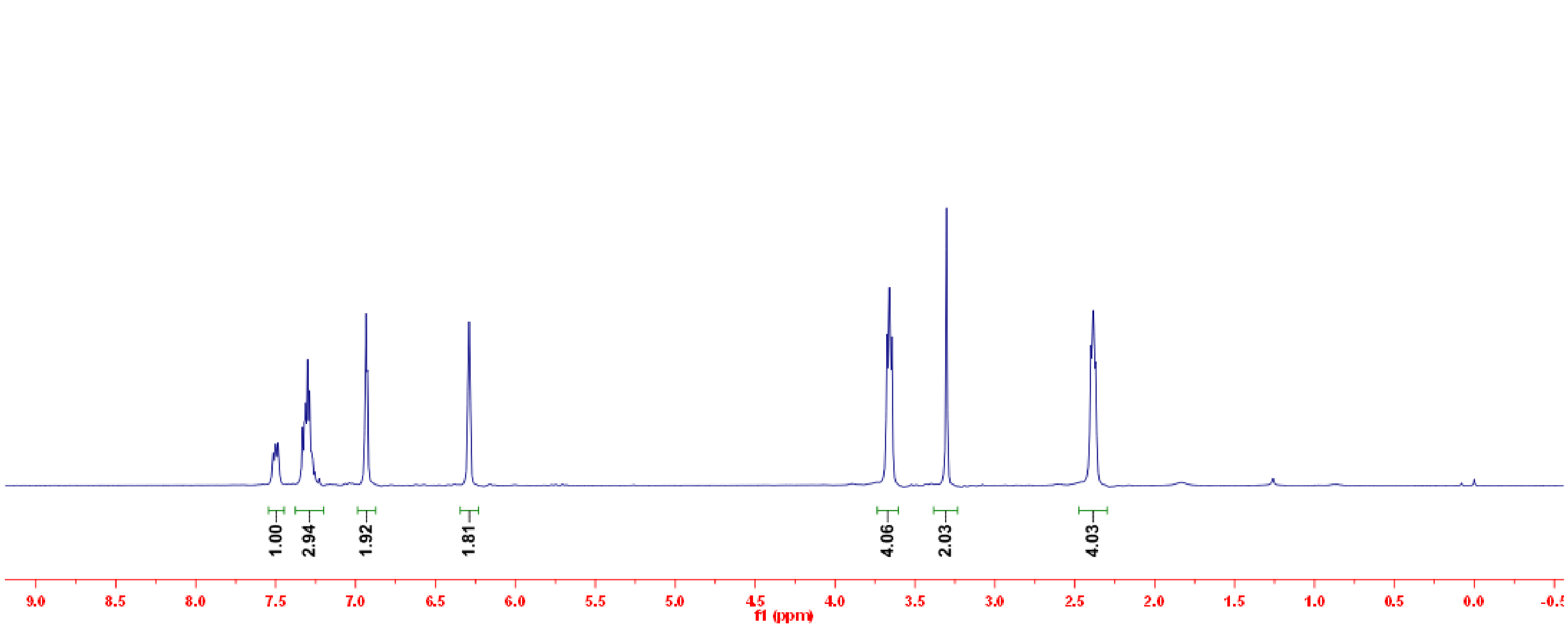
7.5152
7.5025
7.4996
7.4856
7.3115
7.2774
6.9336
6.9269
6.2900
6.2837
3.6747
3.6597
3.6444
3.3020
2.3990
2.3844
2.3698
0.0001
1.00
2.94
1.92
1.81
4.06
2.03
4.03
9.0 8.5 8.0 7.5 7.0 6.5 6.0 5.5 5.0 4.5 4.0 3.5 3.0 2.5 2.0 1.5 1.0 0.5 0.0
f1 (ppm)

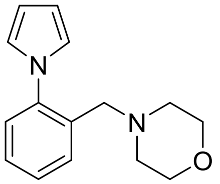
N
N
O

3s

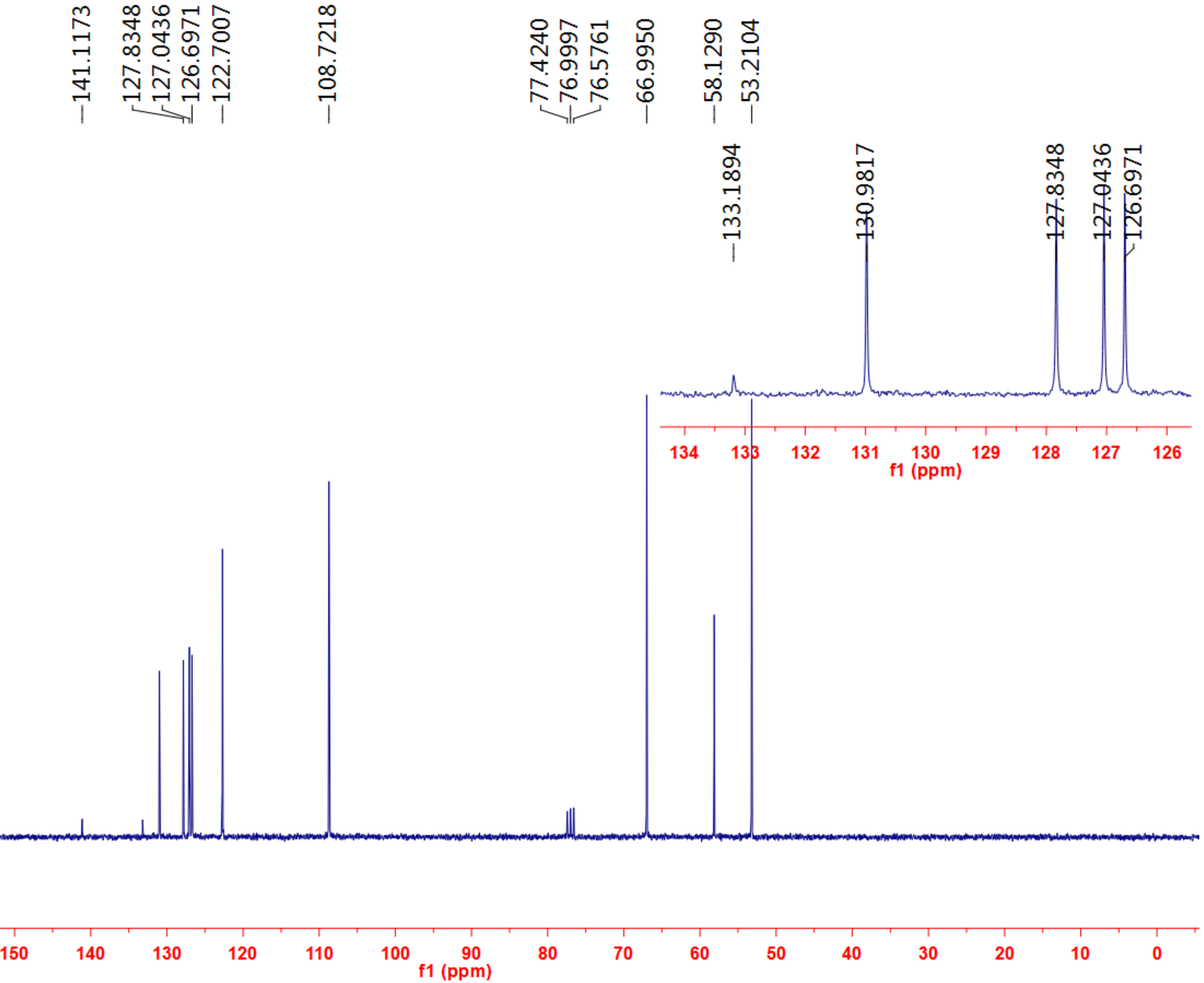
141.1173
127.8348
127.0436
126.6971
122.7007
108.7218
77.4240
76.9997
76.5761
66.9950
58.1290
53.2104
133.1894
130.9817
127.8348
127.0436
126.6971
134 133 132 131 130 129 128 127 126
f1 (ppm)
180 170 160 150 140 130 120 110 100 90 80 70 60 50 40 30 20 10 0
f1 (ppm)

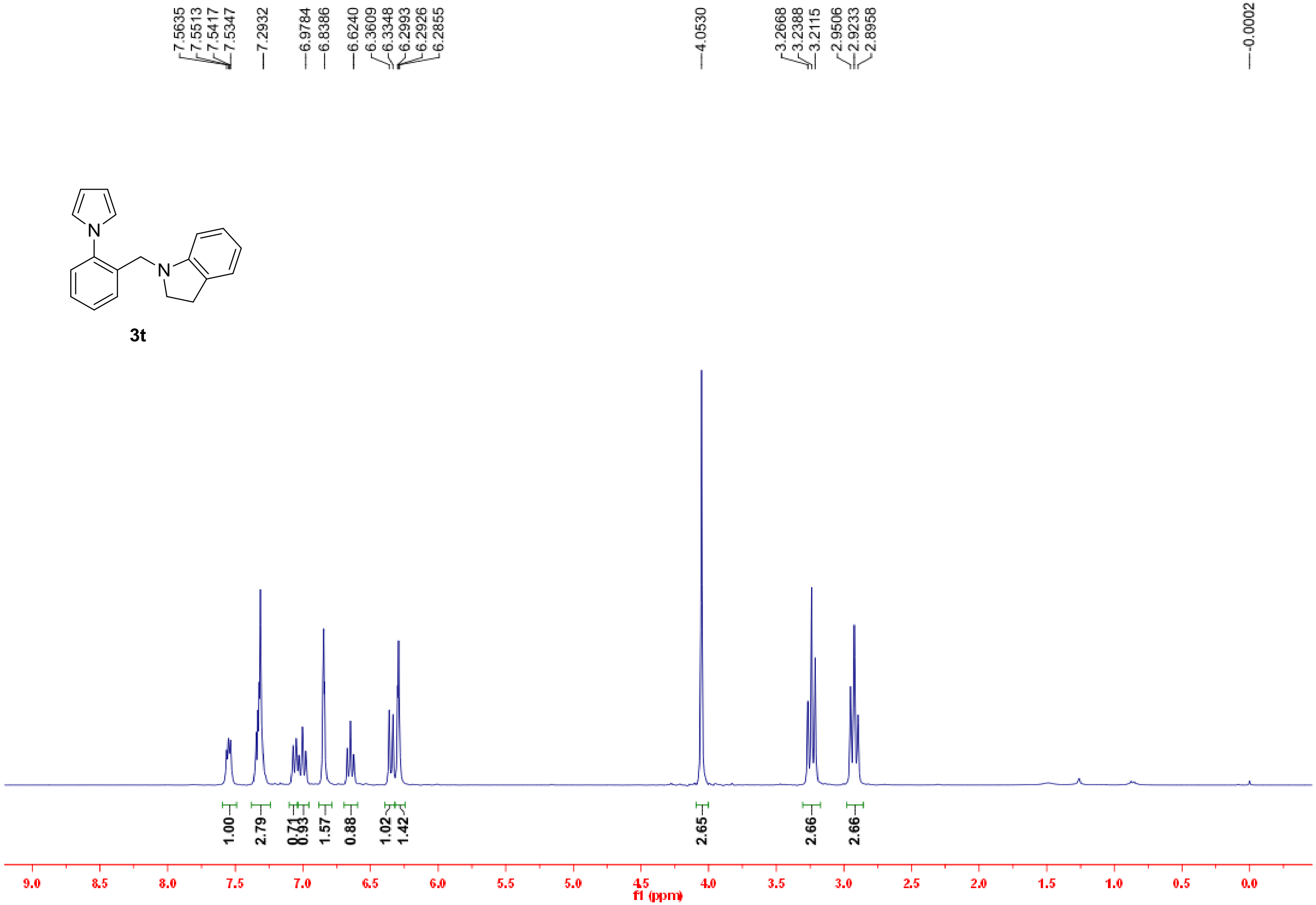
7.5635
7.5513
7.5417
7.5347
7.2932
6.9784
6.8386
6.6240
6.3609
6.3348
6.2993
6.2926
6.2855
4.0530
3.2668
3.2388
3.2115
2.9506
2.9233
2.8958
-0.0002
N
N
3t
1.00
2.79
0.71
0.93
1.57
0.88
1.02
1.42
2.65
2.66
2.66
9.0
8.5
8.0
7.5
7.0
6.5
6.0
5.5
5.0
4.5
4.0
3.5
3.0
2.5
2.0
1.5
1.0
0.5
0.0
f1 (ppm)

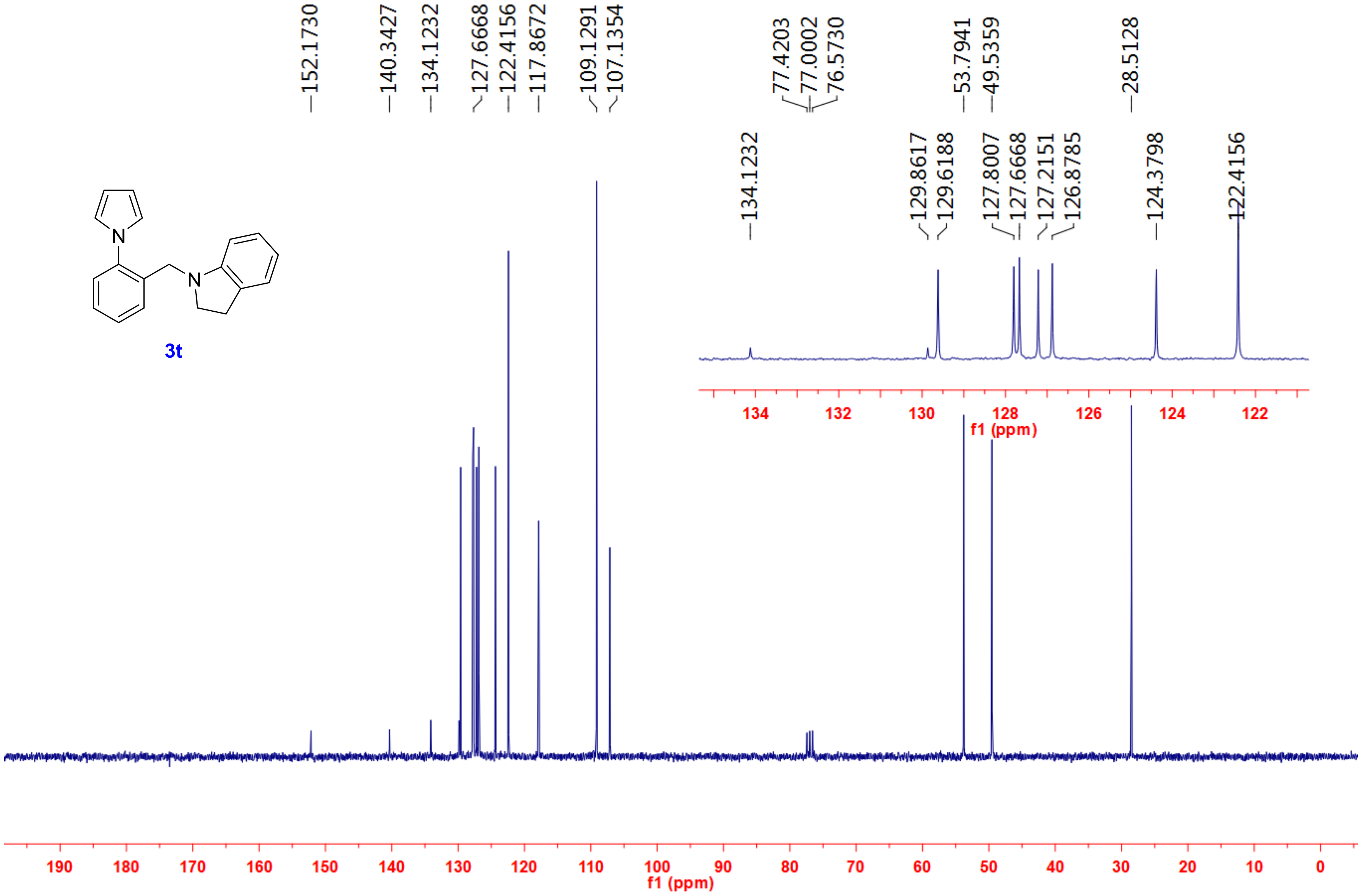
152.1730
140.3427
134.1232
127.6668
122.4156
117.8672
109.1291
107.1354
77.4203
77.0002
76.5730
53.7941
49.5359
28.5128
134.1232
129.8617
129.6188
127.8007
127.6668
127.2151
126.8785
124.3798
122.4156
3t
f1 (ppm)
f1 (ppm)

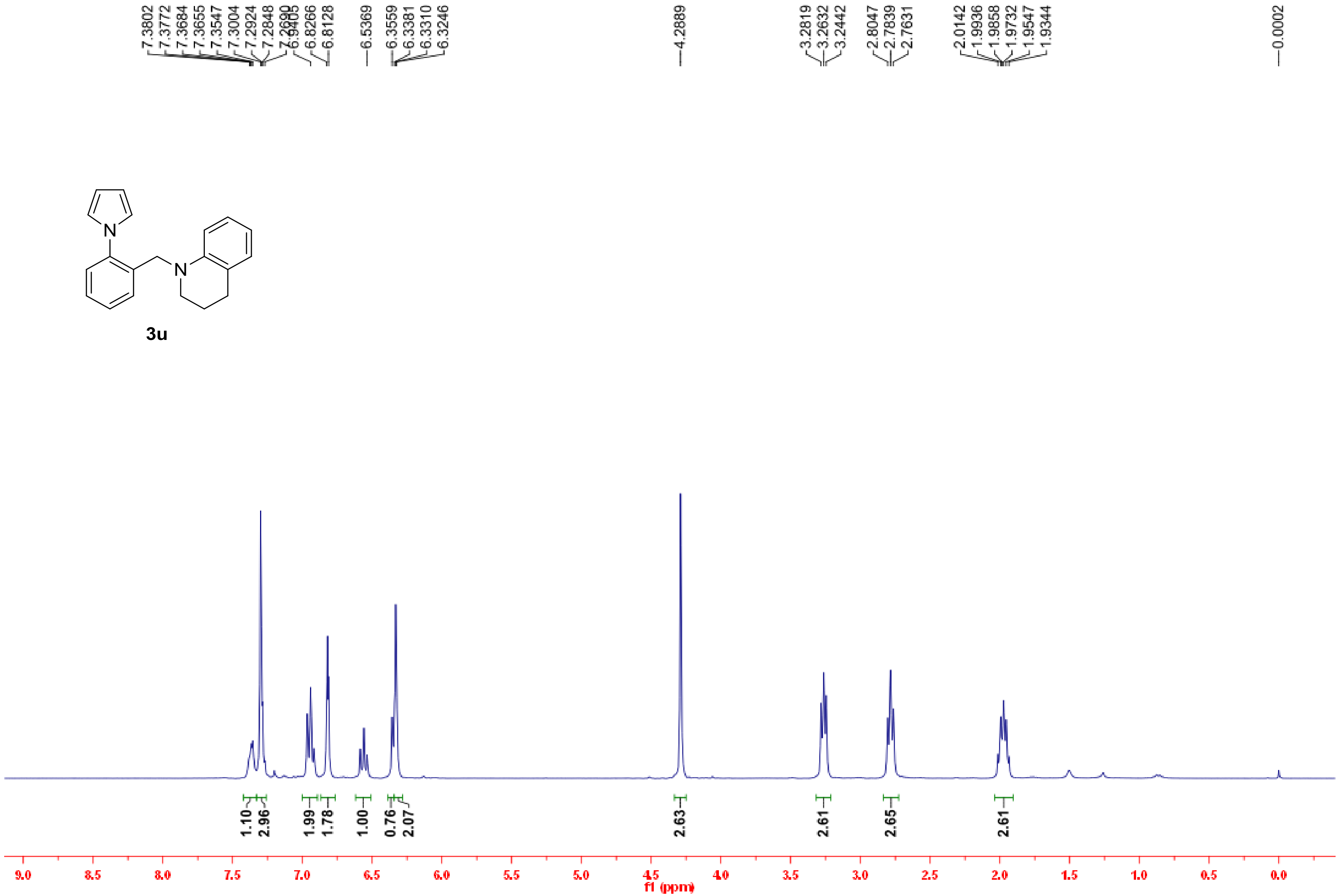
N
N
3u
7.3802
7.3772
7.3684
7.3655
7.3547
7.3004
7.2924
7.2848
7.2690
6.9405
6.8266
6.8128
6.5369
6.3559
6.3381
6.3310
6.3246
4.2889
3.2819
3.2632
3.2442
2.8047
2.7839
2.7631
2.0142
1.9936
1.9858
1.9732
1.9547
1.9344
0.0002
1.10
2.96
1.99
1.78
1.00
0.76
2.07
2.63
2.61
2.65
2.61
9.0
8.5
8.0
7.5
7.0
6.5
6.0
5.5
5.0
4.5
4.0
3.5
3.0
2.5
2.0
1.5
1.0
0.5
0.0
f1 (ppm)

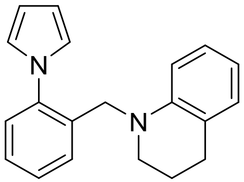

3u

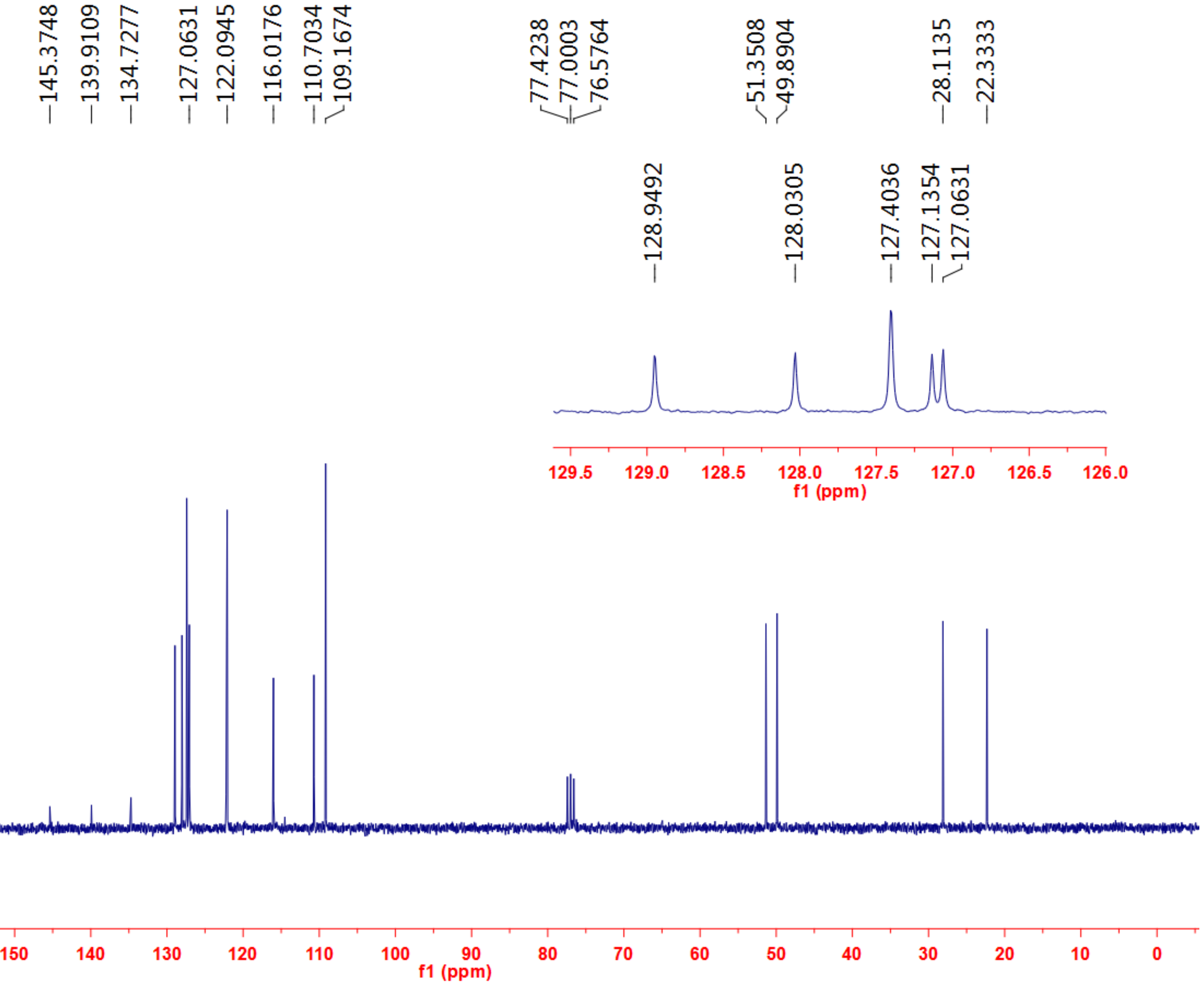

145.3748
139.9109
134.7277
127.0631
122.0945
116.0176
110.7034
109.1674
77.4238
77.0003
76.5764
51.3508
49.8904
28.1135
22.3333
128.9492
128.0305
127.4036
127.1354
127.0631
129.5 129.0 128.5 128.0 127.5 127.0 126.5 126.0
f1 (ppm)
180 170 160 150 140 130 120 110 100 90 80 70 60 50 40 30 20 10 0
f1 (ppm)

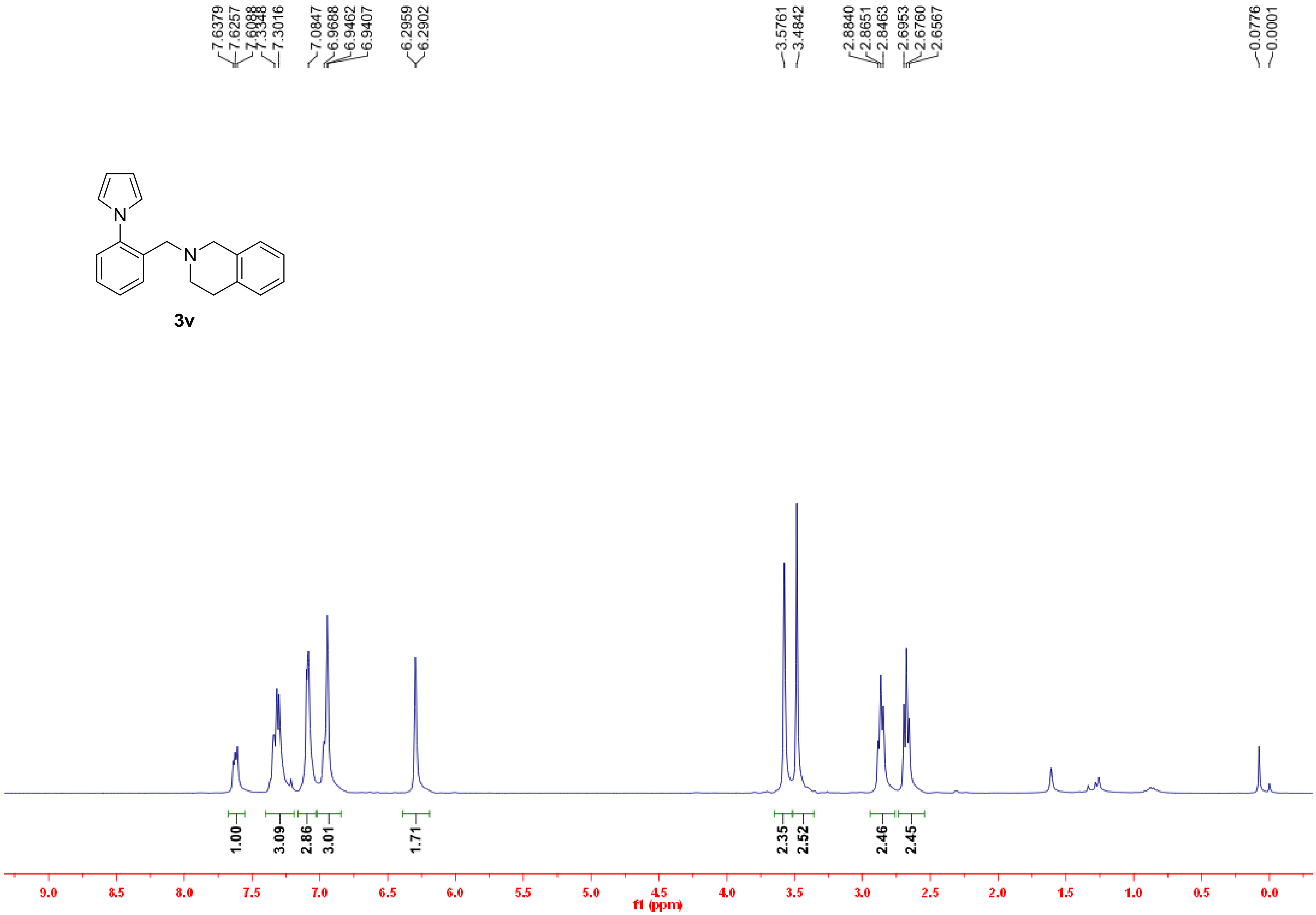
7.6379
7.6257
7.6098
7.3348
7.3016
7.0847
6.9688
6.9462
6.9407
6.2959
6.2902
3.5761
3.4842
2.8840
2.8651
2.8463
2.6953
2.6760
2.6567
0.0776
0.0001
N
N
3v
1.00
3.09
2.86
3.01
1.71
2.35
2.52
2.46
2.45
9.0 8.5 8.0 7.5 7.0 6.5 6.0 5.5 5.0 4.5 4.0 3.5 3.0 2.5 2.0 1.5 1.0 0.5 0.0
f1 (ppm)

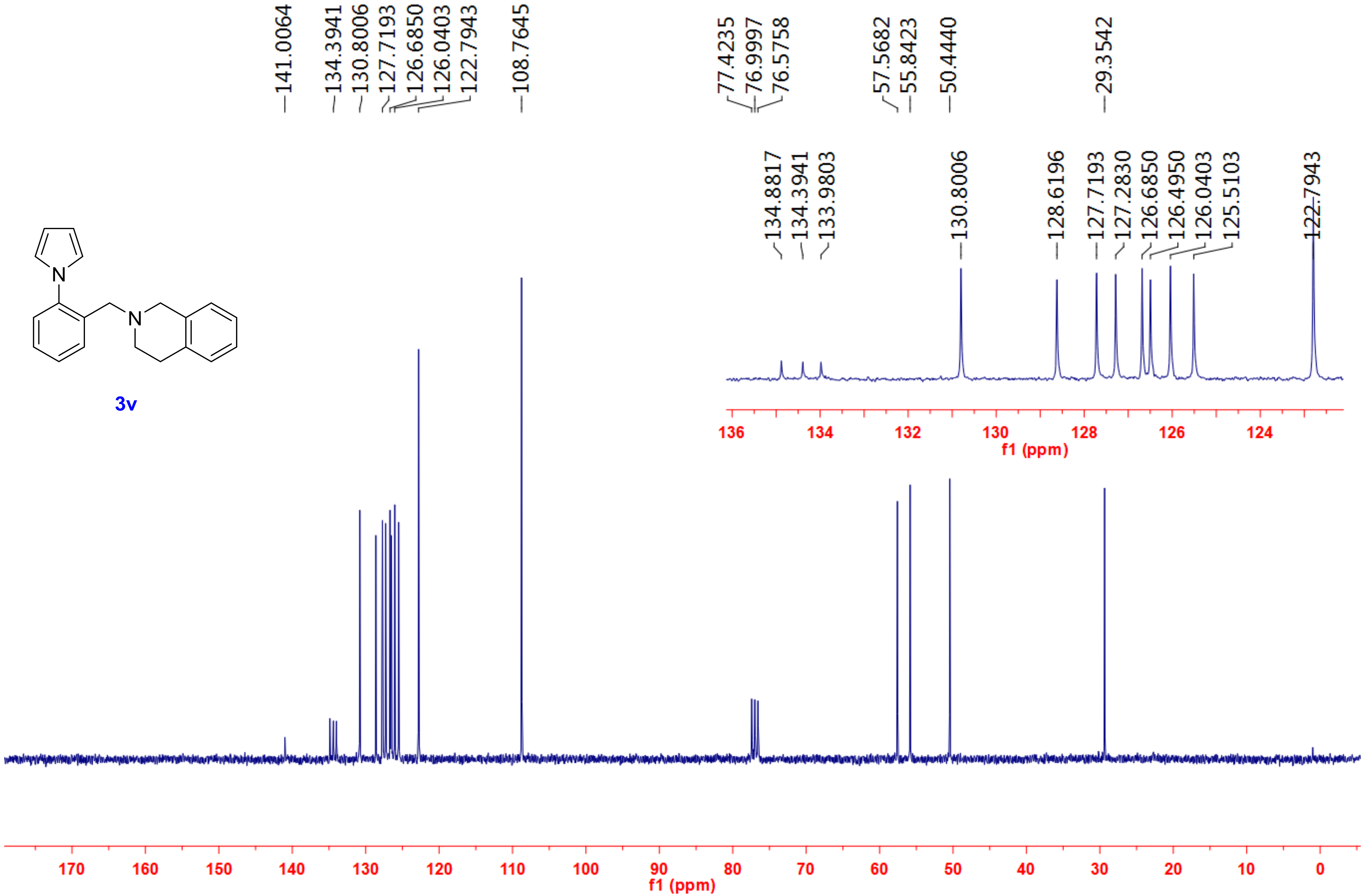
N
N
141.0064
134.3941
130.8006
127.7193
126.6850
126.0403
122.7943
108.7645
77.4235
76.9997
76.5758
57.5682
55.8423
50.4440
29.3542
134.8817
134.3941
133.9803
130.8006
128.6196
127.7193
127.2830
126.6850
126.4950
126.0403
125.5103
122.7943
136
134
132
130
128
126
124
f1 (ppm)
170
160
150
140
130
120
110
100
90
80
70
60
50
40
30
20
10
0
f1 (ppm)

3v

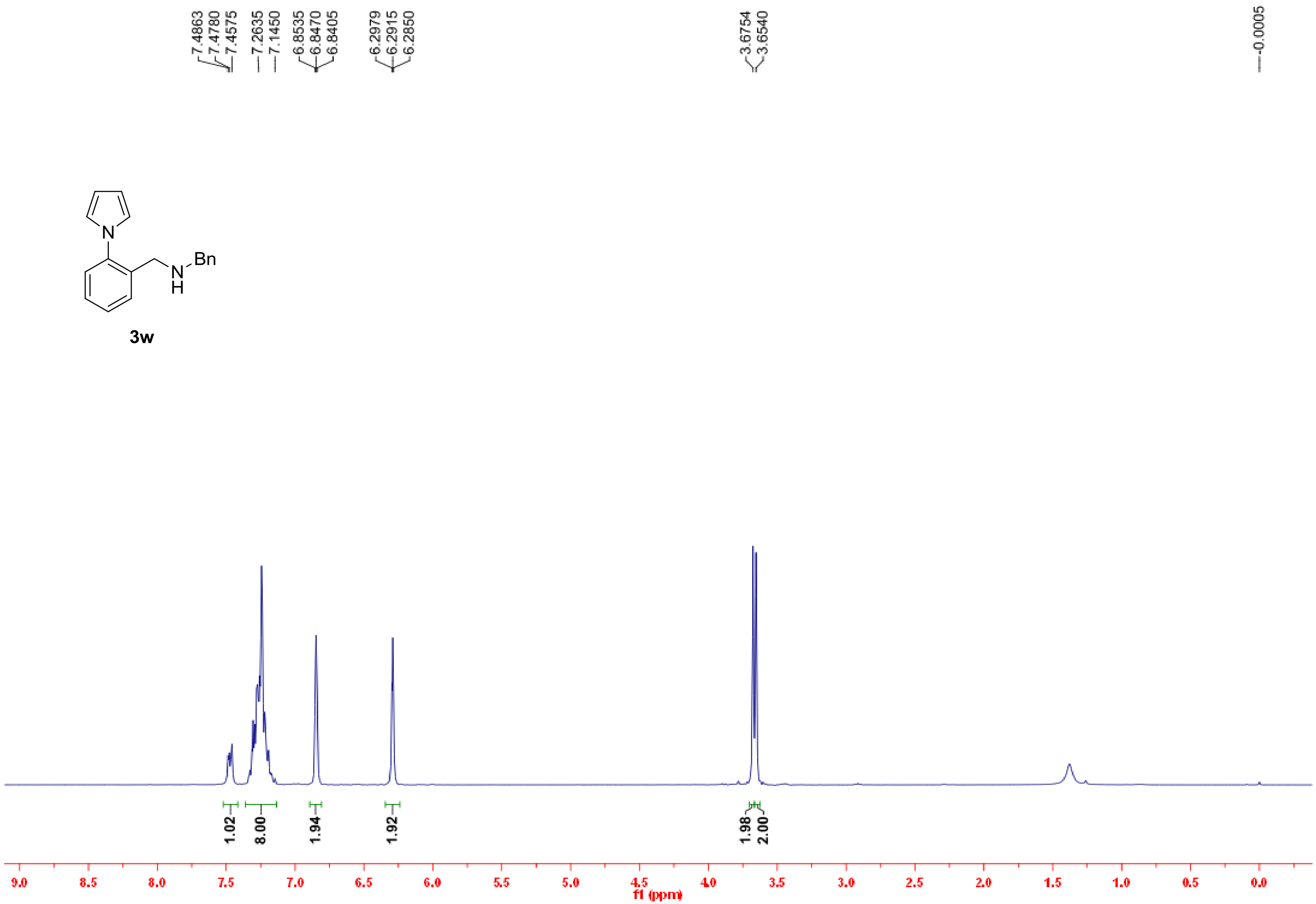
7.4863
7.4780
7.4575
7.2635
7.1450
6.8535
6.8470
6.8405
6.2979
6.2915
6.2850
3.6754
3.6540
-0.0005
N
N
H
Bn
3w
1.02
8.00
1.94
1.92
1.98
2.00
9.0 8.5 8.0 7.5 7.0 6.5 6.0 5.5 5.0 4.5 4.0 3.5 3.0 2.5 2.0 1.5 1.0 0.5 0.0
f1 (ppm)

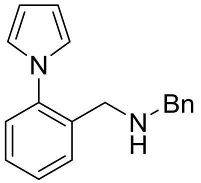
N
N
H
Bn

3w

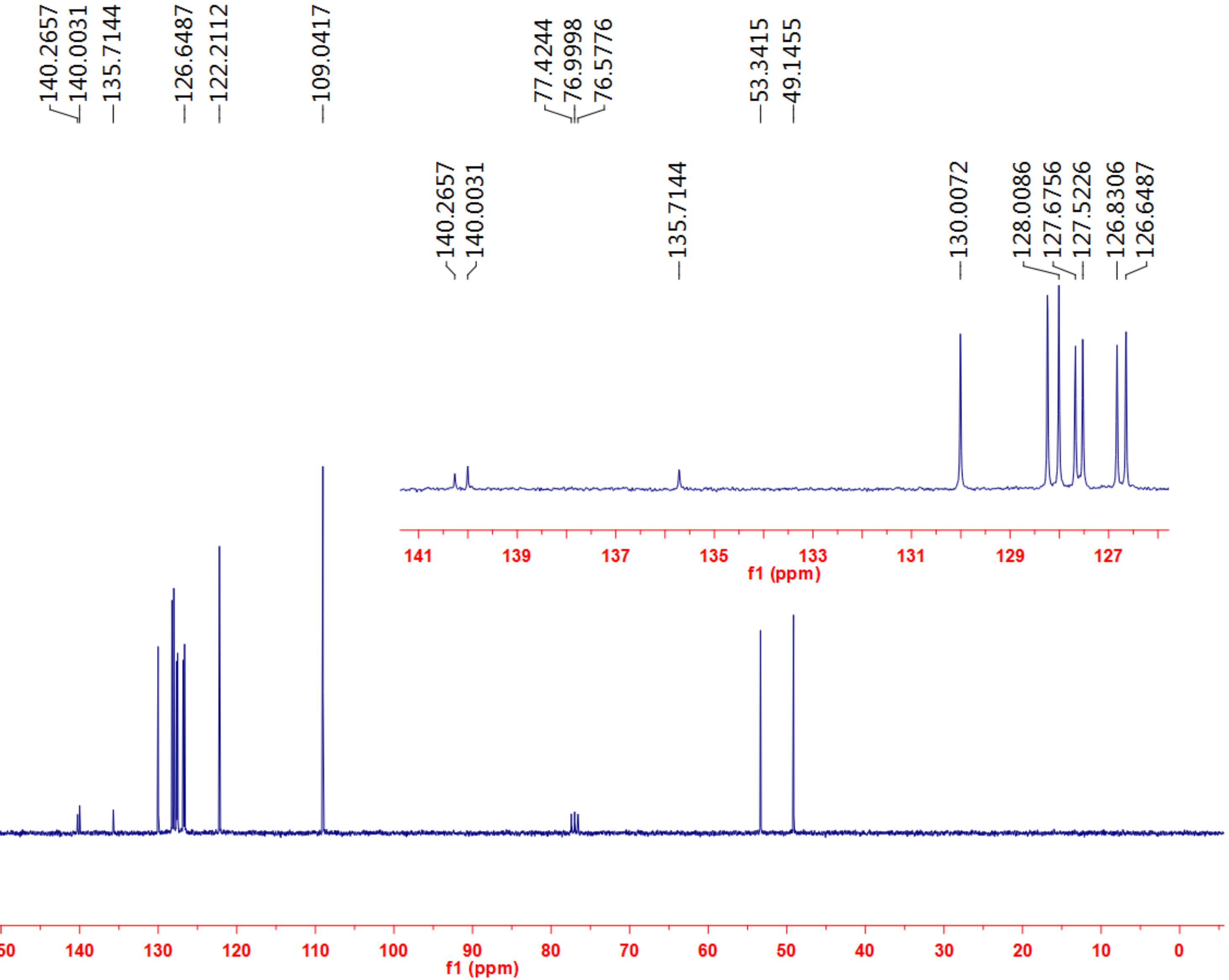
140.2657
140.0031
135.7144
126.6487
122.2112
109.0417
77.4244
76.9998
76.5776
53.3415
49.1455
140.2657
140.0031
135.7144
130.0072
128.0086
127.6756
127.5226
126.8306
126.6487
141
139
137
135
133
131
129
127
f1 (ppm)
180
170
160
150
140
130
120
110
100
90
80
70
60
50
40
30
20
10
0
f1 (ppm)

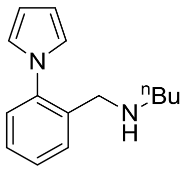
N
nBu
N
H

3x

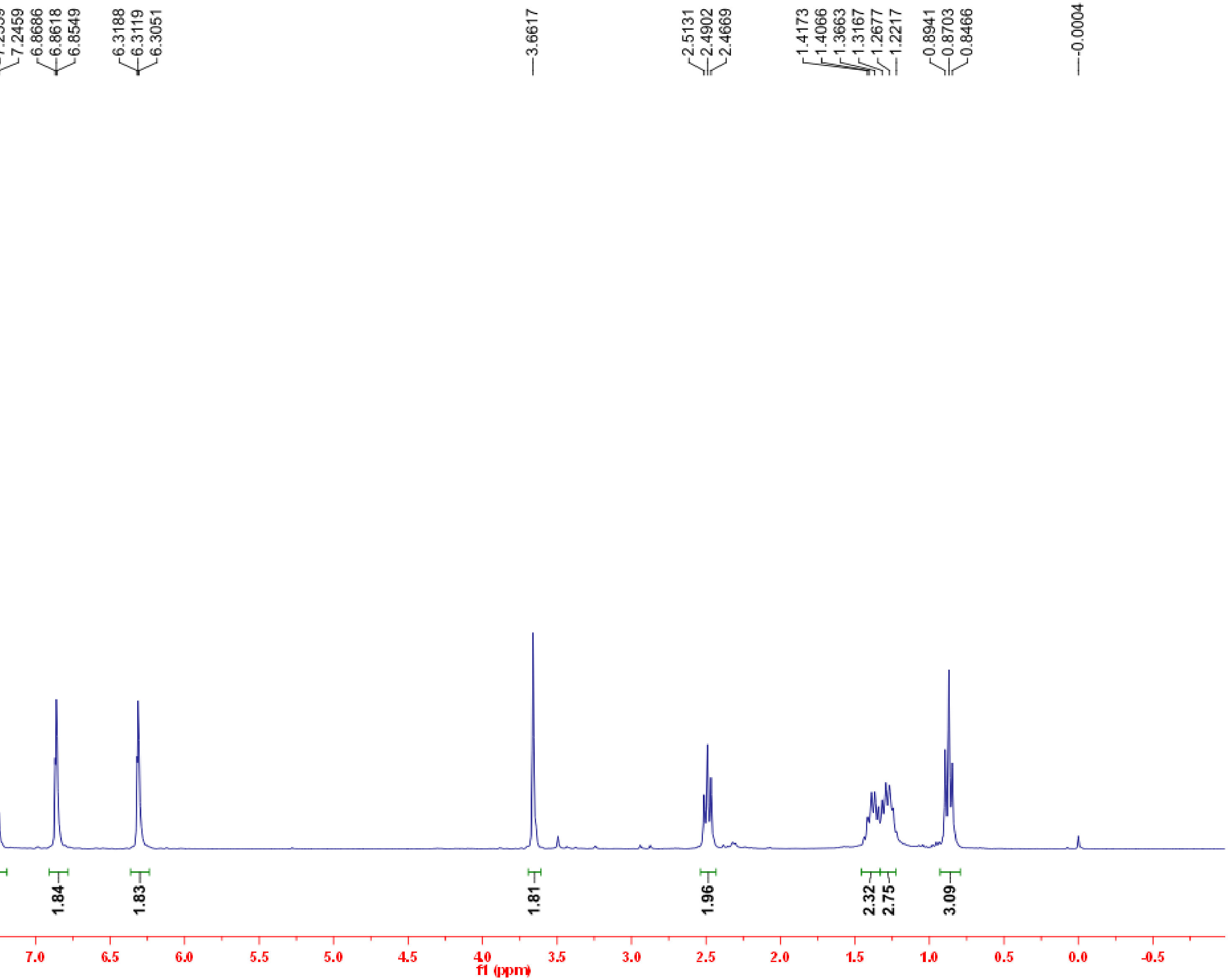
7.4615
7.3609
7.3445
7.3373
7.3341
7.3240
7.3200
7.3133
7.3090
7.3026
7.2790
7.2559
7.2459
6.8686
6.8618
6.8549
6.3188
6.3119
6.3051
3.6617
2.5131
2.4902
2.4669
1.4173
1.4066
1.3663
1.3167
1.2677
1.2217
0.8941
0.8703
0.8466
-0.0004
0.90
3.00
1.84
1.83
1.81
1.96
2.32
2.75
3.09
8.5
8.0
7.5
7.0
6.5
6.0
5.5
5.0
4.5
4.0
3.5
3.0
2.5
2.0
1.5
1.0
0.5
0.0
-0.5
f1 (ppm)

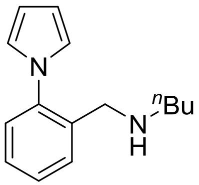
N
N
H
nBu

3x

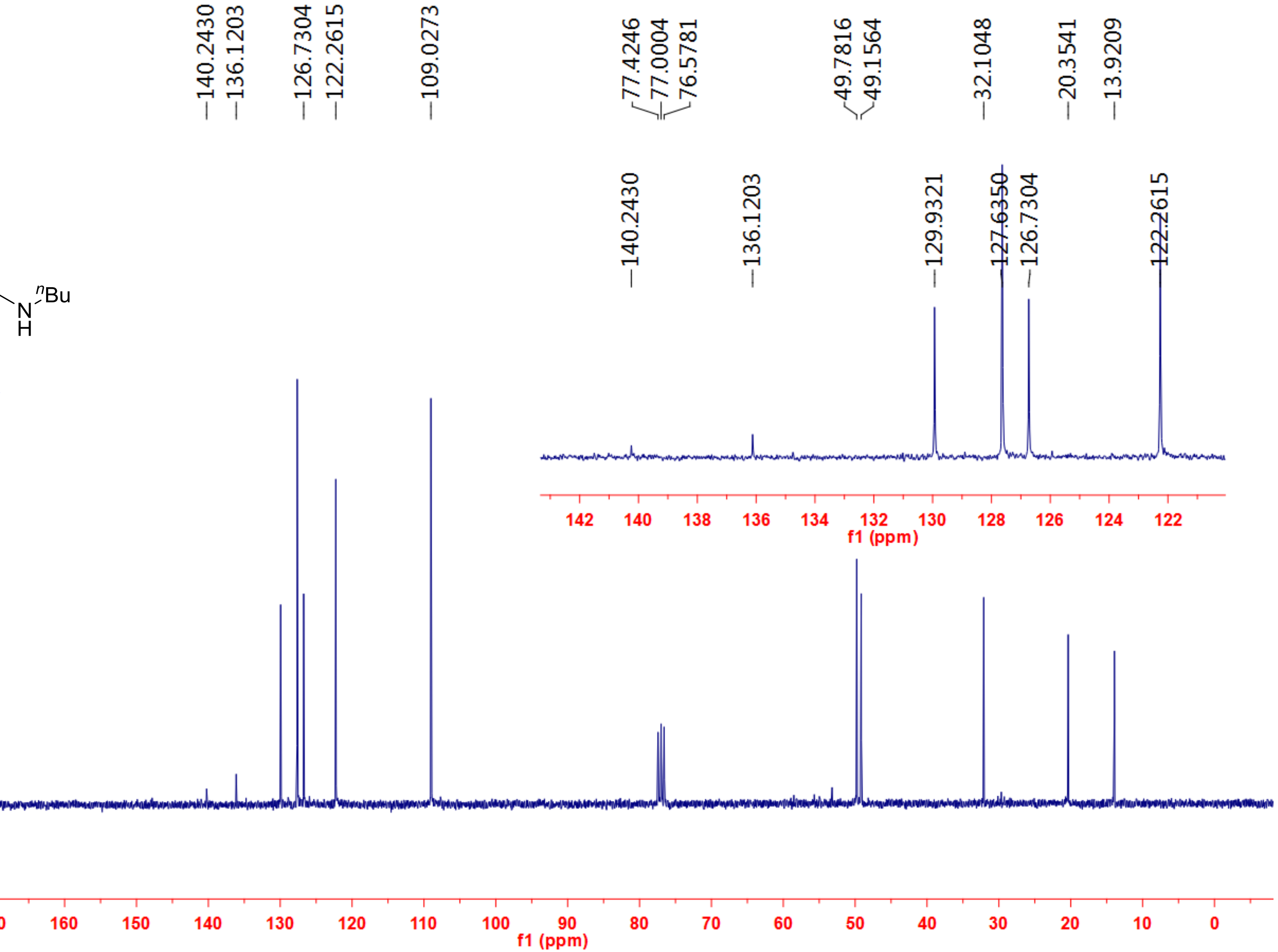
140.2430
136.1203
126.7304
122.2615
109.0273
77.4246
77.0004
76.5781
49.7816
49.1564
32.1048
20.3541
13.9209
140.2430
136.1203
129.9321
127.6350
126.7304
122.2615
142 140 138 136 134 132 130 128 126 124 122
f1 (ppm)
190 180 170 160 150 140 130 120 110 100 90 80 70 60 50 40 30 20 10 0
f1 (ppm)

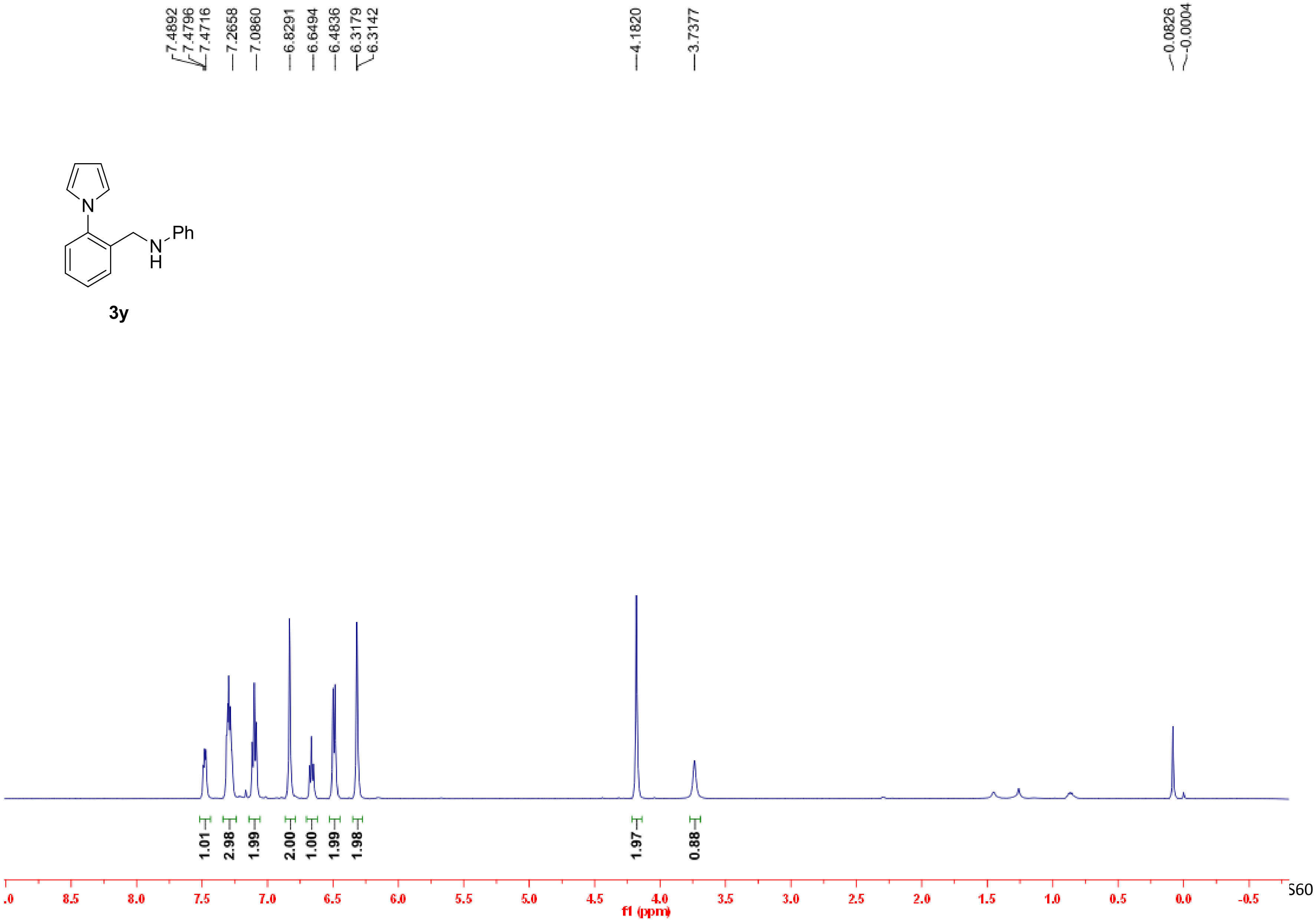
7.4892
7.4796
7.4716
7.2658
7.0860
6.8291
6.6494
6.4836
6.3179
6.3142
4.1820
3.7377
0.0826
-0.0004
N
N
H
Ph
3y
1.01
2.98
1.99
2.00
1.00
1.99
1.98
1.97
0.88
.0
8.5
8.0
7.5
7.0
6.5
6.0
5.5
5.0
4.5
4.0
3.5
3.0
2.5
2.0
1.5
1.0
0.5
0.0
-0.5
f1 (ppm)

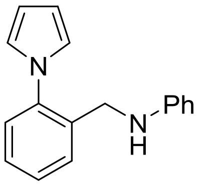
N
N
H
Ph

3y

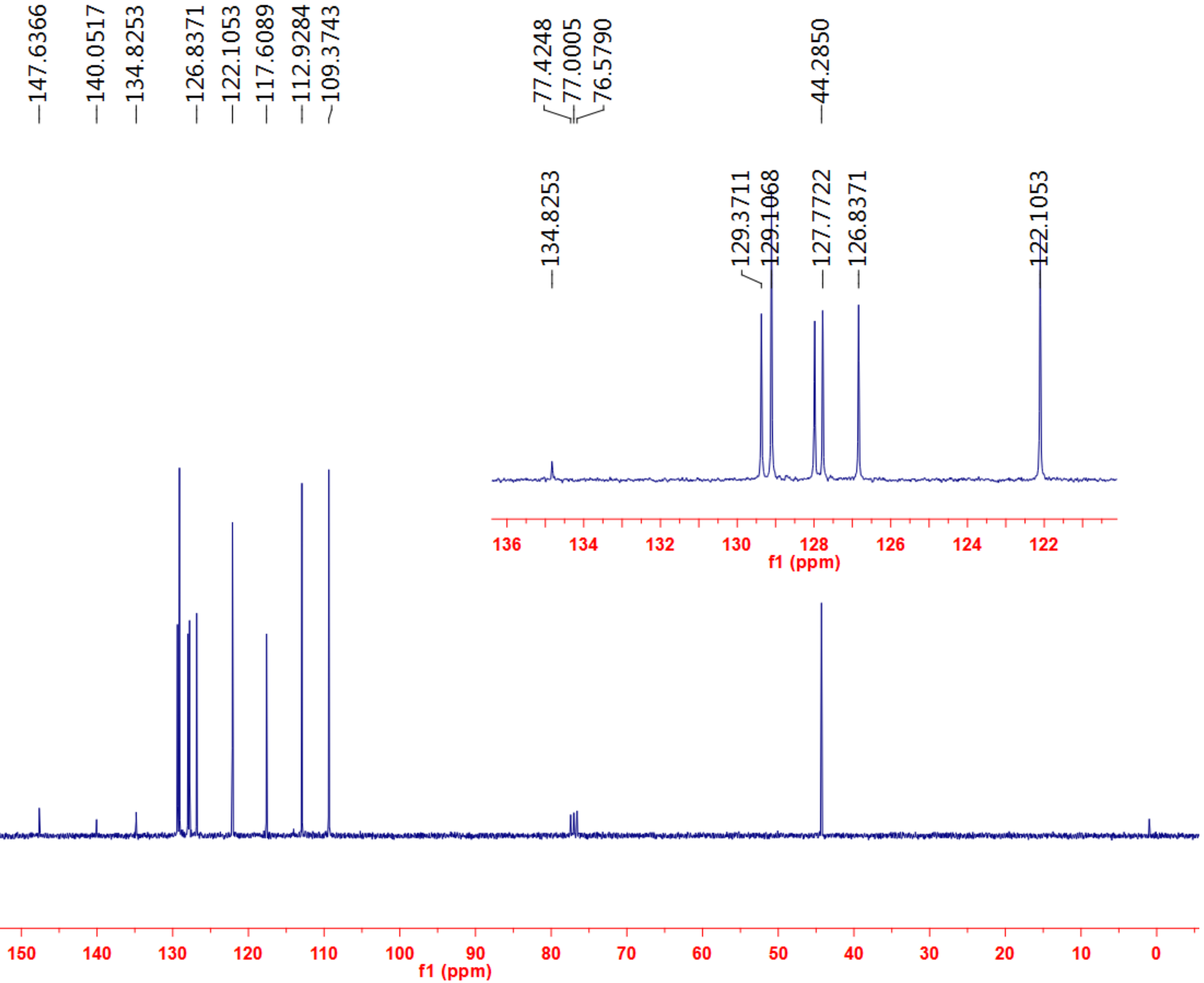
147.6366
140.0517
134.8253
126.8371
122.1053
117.6089
112.9284
109.3743
77.4248
77.0005
76.5790
44.2850
134.8253
129.3711
129.1068
127.7722
126.8371
122.1053
136 134 132 130 128 126 124 122
f1 (ppm)
90 180 170 160 150 140 130 120 110 100 90 80 70 60 50 40 30 20 10 0
f1 (ppm)
